# Organization of the inputs and outputs of the mouse superior colliculus

**DOI:** 10.1038/s41467-021-24241-2

**Published:** 2021-06-28

**Authors:** Nora L. Benavidez, Michael S. Bienkowski, Muye Zhu, Luis H. Garcia, Marina Fayzullina, Lei Gao, Ian Bowman, Lin Gou, Neda Khanjani, Kaelan R. Cotter, Laura Korobkova, Marlene Becerra, Chunru Cao, Monica Y. Song, Bin Zhang, Seita Yamashita, Amanda J. Tugangui, Brian Zingg, Kasey Rose, Darrick Lo, Nicholas N. Foster, Tyler Boesen, Hyun-Seung Mun, Sarvia Aquino, Ian R. Wickersham, Giorgio A. Ascoli, Houri Hintiryan, Hong-Wei Dong

**Affiliations:** 1 Neuroscience Graduate Program, University of Southern California, Los Angeles, CA USA; 2 Stevens Neuroimaging and Informatics Institute, Laboratory of Neuro Imaging, Keck School of Medicine, University of Southern California, Los Angeles, CA USA; 3 McGovern Institute for Brain Research, Massachusetts Institute of Technology, Cambridge, MA USA; 4 Krasnow Institute for Advanced Study, George Mason University, Fairfax, VA USA; 5 Present Address: UCLA Brain Research & Artificial Intelligence Nexus, Department of Neurobiology, David Geffen School of Medicine, University of California Los Angeles, Los Angeles, CA USA

**Keywords:** Network models, Neural circuits

## Abstract

The superior colliculus (SC) receives diverse and robust cortical inputs to drive a range of cognitive and sensorimotor behaviors. However, it remains unclear how descending cortical input arising from higher-order associative areas coordinate with SC sensorimotor networks to influence its outputs. Here, we construct a comprehensive map of all cortico-tectal projections and identify four collicular zones with differential cortical inputs: medial (SC.m), centromedial (SC.cm), centrolateral (SC.cl) and lateral (SC.l). Further, we delineate the distinctive brain-wide input/output organization of each collicular zone, assemble multiple parallel cortico-tecto-thalamic subnetworks, and identify the somatotopic map in the SC that displays distinguishable spatial properties from the somatotopic maps in the neocortex and basal ganglia. Finally, we characterize interactions between those cortico-tecto-thalamic and cortico-basal ganglia-thalamic subnetworks. This study provides a structural basis for understanding how SC is involved in integrating different sensory modalities, translating sensory information to motor command, and coordinating different actions in goal-directed behaviors.

## Abstract (web summary)

The superior colliculus (SC) receives diverse cortical inputs to drive many behaviors. Here, based on comprehensive mapping of cortico-tectal projections, the authors refined the superior colliculus into medial, centromedial, centrolateral, and lateral zones, and characterized the input-output connectivity and morphology of neurons in each zone that serve the role of SC in goal-directed behaviors.

## Introduction

Cortico-tectal projections from the cerebral cortex to the superior colliculus (SC) relay information to mediate a range of multimodal and cognitive functions^1–4^. In order for the mammalian SC to coordinate complex behaviors, such as attention, navigation, defense, and decision-making, it requires the alignment and integration of top-down higher-order cortical information with sensorimotor maps. Topographic cortico-tectal projections from primary visual, auditory, and motor areas have been well studied; however, the integration of higher-order association cortical inputs to the SC has not been thoroughly characterized^3,5,6^. Disruptions in cortico-tectal projections have been implicated in attention-deficit and autism spectrum disorders^7–11^; thus, it is important to understand the organizational principles of how the confluence of higher-order cortical information is integrated in the SC and channeled to downstream structures to regulate and coordinate behavioral outputs. An anatomical parcellation of cortico-tectal pathways would offer a resourceful and translational reference map.

At present, the mouse SC is regarded as an emergent structural model for the rodent visual system and the circuit formation that facilitates multisensory integration^12,13^. Although there has been a prolific rise in rodent functional studies, the connectivity of the SC is often reviewed and generalized across multiple species to suggest there is little left to understand about its mesoscale connections^12,14,15^. Nonetheless, to date, there is no complete anatomical map of SC connectivity in mice or any other species^16,17^. Thus, as part of current advancements within the large collaborative effort of the BRAIN Initiative Cell Census Network (BICCN) to identify detailed mouse brain anatomy (https://biccn.org/), we are contributing by mapping the mouse SC connectome using modern neuroanatomic and computational tools. Here, combining state-of-the-art circuit mapping and viral tracing methods with computational neuroanatomical tools, we assembled the first detailed connectivity map of descending cortical subnetworks to the SC, the cortico-tectal projectome, and concurrently constructed a brain-wide wiring diagram for the SC. This comprehensive resource provides a foundational structural model for the mouse SC that supports working hypotheses of regionally defined functions (such as flight vs. freezing or approach vs. aggression), and network- and cell-type-specific functions.

In this study, we expand upon the laminar organization of the SC by further delineating its regional organization. The SC has a distinct composition of seven alternating fibrous and cellular layers, including three superficial (zo, zonal; sg, superficial gray; op, optic), two intermediate (ig, intermediate gray; iw, intermediate white), and two deep layers (dg, deep gray; dw, deep white)^18^. Functional studies often arbitrarily bisect the SC into two medial and lateral halves^19^; however, these broad divisions can be refined to reflect how distinct subnetworks of contiguous or segregated inputs/outputs are organized throughout the SC. To address this, we systematically characterized layer-specific cortical fiber terminations across the entire SC to define subregional zones. Analyses of the distribution of cortical-tectal projections revealed that the SC could be subdivided into four radially distinct zones that extend along the medial-lateral and rostral-caudal axes. Each zone, which is defined by a unique subnetwork of cortical inputs, displays a distinct brain-wide input-output organization. The circuits are correlated with specialized functional subnetworks involved in the integration of visual, auditory, and somatosensory modalities, and are highly conserved topographically^20,21^. These four subnetworks of the SC provide a structural basis for understanding different functional roles of the SC in coordinating movements of eyes, head and neck, whisker, mouth, and limbs in goal-oriented tasks such as attentive orientation, spatial navigation, and exploratory (i.e., prey capture), appetitive/approach, consummatory (i.e., chewing, swallowing, and biting) behaviors.

## Results

### General strategy for delineating the cortico-tectal projectome

**Fig. 1:**
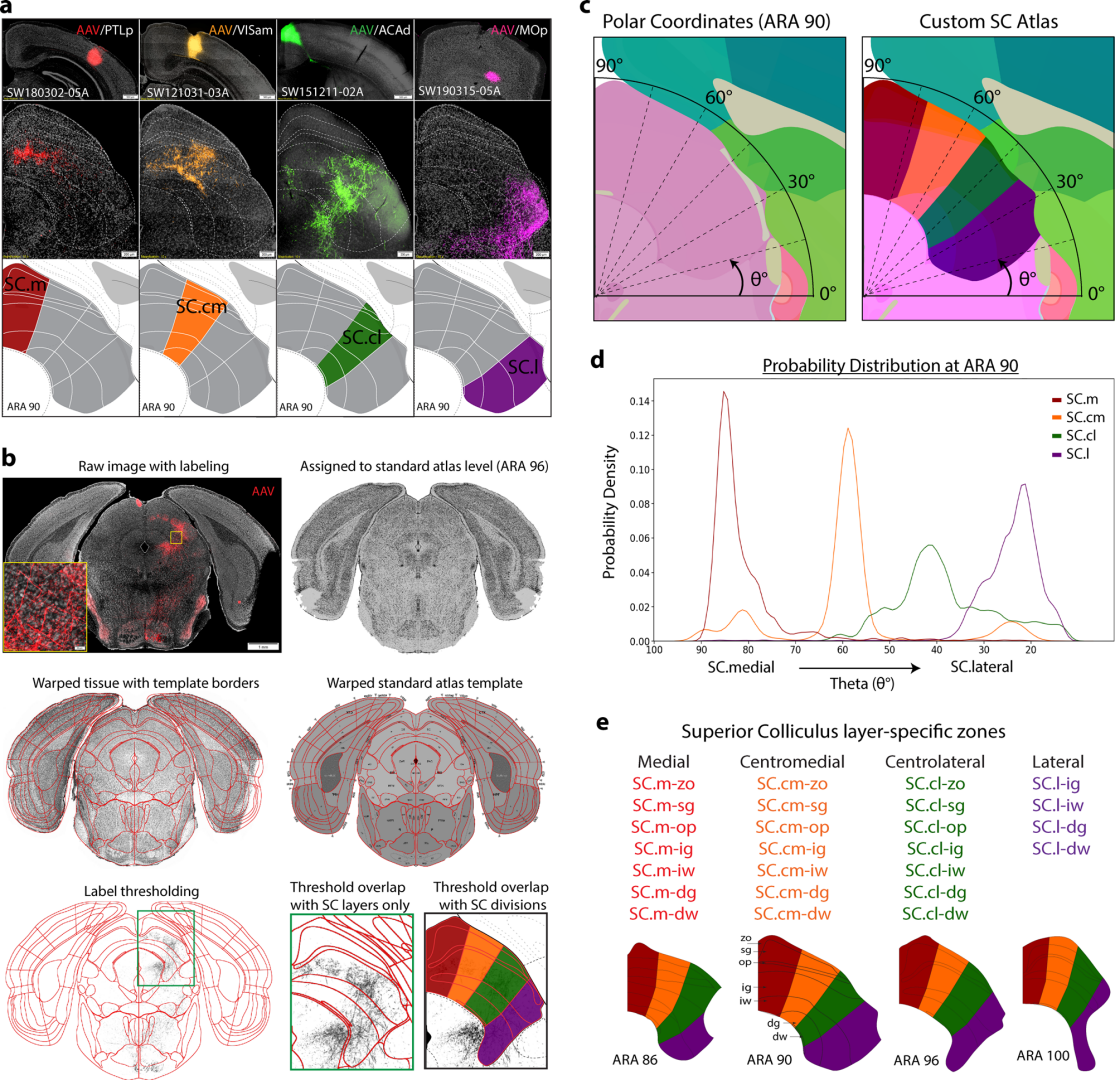
Experimental workflow. **a** Raw data examples of cortico-tectal projections targeting distinct zones within SC layers at ARA 90. Anterograde AAV injections into PTLp→SC.m (red), VISam→SC.cm (orange), ACAd→SC.cl (green), and MOp→SC.l (purple) terminate with different laminar and nonoverlapping patterns. Together, these representative cortico-tectal projections reveal layer-specific terminals distributed across four distinct zones along the medial-lateral axis of SC. Scale bar at injection sites is 500 µm, and 200 µm in lower SC panels. **b** The Connection Lens neuroinformatics workflow: Raw tissue image with anterograde labeling is assigned to the corresponding standard atlas level (this example is ARA 96). The tissue is warped based on template atlas borders and reconstructed using in-house neuroinformatics software. Thresholded images are overlapped onto a custom atlas for zone- and layer-specific registration for pixel quantification. **c** Left: Polar Coordinate analysis method used to quantify angular distribution of thresholded pixel labeling in SC. Angles represented by theta (θ°) values where midline starts at 90° and ranges toward 0° at lateral angles. Right: Custom SC atlas with overlay of angular range shows coarse alignment of manually delineated borders in SC. **d** Example of probability distribution graph for average distributions of zone-specific cortico-tectal cases. Peaks are aligned with custom SC borders at ARA 90. SC.m (red) is SW180522-04A. SC.cm (orange) is SW121221-03A. SC.cl (green) is SW171130-02A. SC.l (purple) is SW170410-04A. **e** Layer- and zone-specific SC nomenclature to facilitate referencing and quantification. Below: zone delineations across representative ARA levels (86, 90, 96, 100) spanning rostral-to-caudal SC. ACAd anterior cingulate cortex dorsal part, ARA Allen reference atlas, AAV Adeno-associated virus, PHAL *Phaseolus vulgaris* leucoagglutinin, PTLp posterior parietal cortex, MOp primary motor cortex, VISam visual cortex anterior medial part, SC superior colliculus, SC Layers: zo zonal, sg superficial gray, op optic, ig intermediate gray, iw intermediate white, dg deep gray, dw deep white.

First, we systematically analyzed and annotated the cortico-tectal projection patterns of 86 anterograde injections placed across the entire cortex in adult mice (*n* = 44) (Supplementary Fig. 1). Single anterograde tracer injections of either *Phaseolus vulgaris* leucoagglutinin (PHAL), or adeno-associated viruses expressing green (AAV-GFP), or red (AAV-tdTomato) fluorescent protein were confined to a single cortical area to produce regional specific projection terminal patterns in the SC. We also carried out double or triple tracer injections with combinations of PHAL, AAV-GFP, and AAV-tdTomato to determine direct spatial correlations of axonal terminals arising from different cortical areas. Notably, though we present a representative sample of cortical regions, the discrete volume of our tracer injections may also render some regions under sampled. To conduct systematic analyses, raw microscopic images with tracer-labeled cortico-tectal projections from each individual experimental case were assigned to their corresponding atlas template levels from the adult mouse brain Allen Reference Atlas (ARA)^22^ (see Methods). Our manual annotations revealed that each cortical area sent unique efferents to distinct layers and regions in the SC. Some examples from representative cases include (a) terminals from the primary motor area  (MOp) preferentially targeting the furthest lateral region in SC; (b) dorsal anterior cingulate area (ACAd) terminals targeted a region more dorsal-medially adjacent to MOp; (c) anteromedial visual area (VISam) was more medially adjacent to ACAd; and (d) posterior parietal association area (PTLp) was more medial to VISam adjacent to the midline (Fig. 1a). Terminals from these cases often spanned multiple SC layers, but displayed distinct layer specificities (Supplementary Tables 3 and 4). These projections reveal four distinctive columns or zones arranged from the midline to the lateral border of the SC. Comparisons across multiple cases (*n* = 44) reveal a consistent distribution pattern which we qualitatively delineated into regional zones of our custom SC atlas: SC medial (SC.m), centromedial (SC.cm), centrolateral (SC.cl), and lateral (SC.l) (Fig. 1a). Although other patterns and organization schemas may exist within SC^19^, this distribution pattern as determined by the cortical inputs suggests that it may be a more refined, compact and anatomically relevant way to analyze the organization of these circuits. The complete list of abbreviations and nomenclature can be found in Supplementary Table 1. Injection sites and tracer details for all cases used throughout this study can be found in Supplementary Table 2.

To quantitatively test the prominence of these four zones from cortico-tectal inputs, we applied computational neuroanatomical methods^20,21^ to analyze the representative cortico-tectal pathways that span the rostral-to-caudal and medial-to-lateral extent of the cerebral cortex and SC. The selected images for analysis were registered to their corresponding atlas templates at ARA levels 86, 90, 96, and 100, representative of the rostral, intermediate and caudal SC levels (Supplementary Fig. 2). Anterograde labels were then thresholded, graphically reconstructed, and overlapped onto the atlas templates using our in-house Connection Lens software^21^ (Fig. 1b). Pixels that were verified as fibers of passage and that did not represent axonal terminals were excluded from quantification analysis. A polar coordinate analysis was designed and applied to visualize how terminal pixel densities are distributed around the radial axis of SC (across angular degrees relative to the midline) (Fig. 1c). The probability distribution for cortico-tectal projections was plotted as a smoothed histogram, illustrating the overlap and similarities of projection profiles for multiple cortical cases within distinct ranges of SC. Probability distribution plots show individual cortical projections that target distinct zones (SC.m (*n* = 7), SC.cm (*n* = 4), SC.cl (*n* = 12), and SC.l (*n* = 20)) (Fig. 1d, Supplementary Fig. 2). Average distributions clearly demonstrate the prominence of four peaks along the medial-to-lateral axis validating the raw data, and the peaks are congruent with the manually delineated zones of our custom SC atlas. The peaks are also generally continuous across the rostral-to-caudal axis, though there may be other methods to parcellate the SC along this axis. We designated zone- and layer-specific nomenclature to facilitate more detailed referencing and quantification of cortical inputs to the SC (Fig. 1e). We hypothesize that these custom SC atlas zones reflect subnetwork communities similar to those previously identified in cortico-cortical and cortico-striatal subnetworks^20,21^.

### Visual, somatosensory, and motor cortex projection maps in SC

**Fig. 2:**
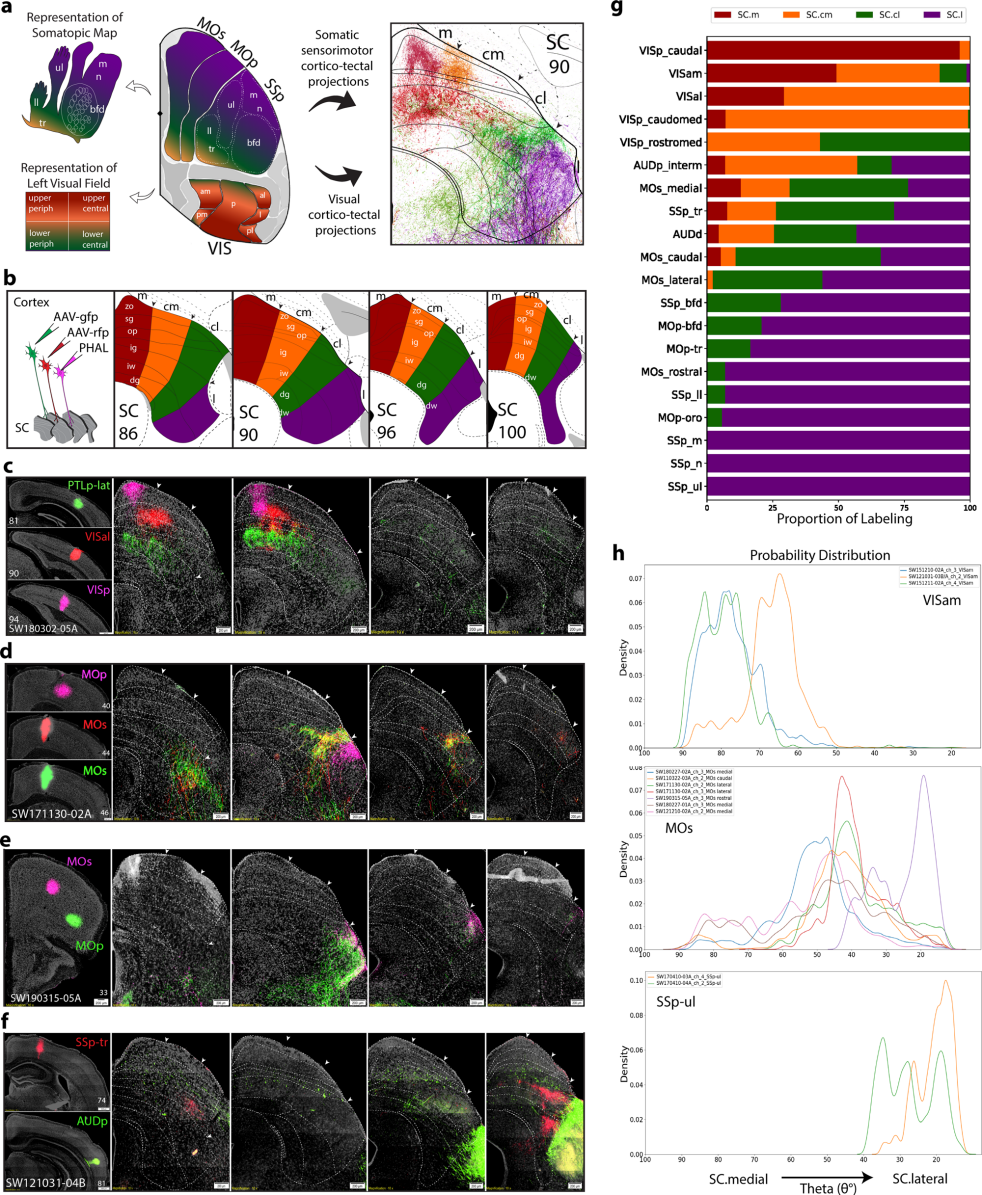
Visual, auditory, and somatic sensorimotor map of cortical projections to SC zones. **a** Color-coded schematic overview of the left visual field and body part topography based on their target zones in right hemisphere of SC at level 90. Reconstructions of cortico-tectal fibers adjacent. **b** Schematic of injection strategy using triple or double anterograde tracers into cortex, and custom SC atlas levels. **c** VISp, VISal, and PTLp-lat projections to SC.m and SC.cm. SC boundaries in all panels were delineated based on Nissl-stained cytoarchitecture. Dashed lines correspond to specific layers in each SC level. Scale bar 200 µm in SC panels. **d** Two neighboring caudal MOs regions and a rostral MOp injection send projections to adjacent, but nonoverlapping zones in the SC.cl and SC.l. **e** Rostral MOs and rostral-ventral MOp projections to the SC.l zone. **f** SSp-tr and AUDp projections target caudal SC.l zones. **g** Stacked bar chart for visualization of proportion of labeling across each SC zone (*x*-axis) from each cortical ROI (*y-*axis) for each selected ROI (*n* = 20 cases). Values represent proportion of pixel density for an individual ROI across each zone (*n* = 1 case per row). See Supplementary Table 3 for zone- and layer-specific values. **h** Probability distribution graphs of thresholded labeling represented by probability density (*y*-axis) across angular ranges (θ°) in SC (*x*-axis) from atlas level 90. VISam cases show distributions in angular ranges that align with SC.m and SC.cm zones (*n* = 3). MOs cases show distributions aligned with SC.cl (*n* = 10). SSp-ul cases show distributions in angular ranges aligned with SC.l (and SC.cl) zones (*n* = 3). Color-code associations: red (SC.m), orange (SC.cm), green (SC.cl), purple (SC.l). ACAd anterior cingulate cortex dorsal part, ARA Allen reference atlas, AAV Adeno-associated virus, AAV-gfp AAV green fluorescent protein, AAV-rfp AAV red fluorescent protein, MOp primary motor cortex, MOs secondary motor cortex, PHAL *Phaseolus vulgaris* leucoagglutinin, PTLp posterior parietal cortex, VISam visual cortex anterior medial part, SC superior colliculus, SC Layers: zo zonal, sg superficial gray, op optic, ig intermediate gray, iw intermediate white, dg deep gray, dw deep white, Somatotopic body parts; bfd barrel field, ll lower limb, m mouth, n nose, tr trunk, ul upper limb, SSp primary somatosensory cortex, VIS visual cortex. Complete list of abbreviations in Supplementary Table 1.

We constructed an anterograde cortico-tectal projection map based on neural inputs arising from visual, auditory, and somatic sensorimotor areas (Fig. 2a, b). The primary visual cortical area (VISp) exclusively targets superficial SC layers, while the secondary visual areas, including the anterolateral (VISal), lateral (VISl), anteromedial (VISam), and posteromedial (VISpm), target both the superficial and intermediate layers (Fig. 2c; Supplementary Fig. 3a). The SC.m and SC.cm receive input from all visual cortices, and represent the upper central and upper peripheral visual field. The SC.cl receives direct inputs from the VISp-rostromedial domain that represents the lower central and lower peripheral field, VISam and auditory cortex. Furthermore, the SC.cl receives dense inputs from the MOs-medial domain that is equivalent to primate frontal-eye field (MOs-fef) (see Zingg et al., 2014).

From the inherent cytoarchitecture of the SC, the three superficial visual layers do not extend into the far lateral SC, thereby distinguishing the SC.l from the other three zones. The SC.l does not receive any visual inputs; instead, it predominantly receives inputs from all somatic sensorimotor cortical areas (Fig. 2d–f). These cortical projections are topographically distributed in a unique somatotopic order. The rostral SC.l dominantly receives denser inputs from the SSp-m (mouth), SSp-n (nose), and SSp-ul (upper limb), and SSp-bfd (barrel field). By contrast, the SSp-ll (lower limb) and SSp-tr (trunk) project more densely to the caudal levels of SC.l with extension into the SC.cl (Supplementary Fig. 3b, c). Similarly, all MOp body domains predominantly project across the SC.l overlapping with their counterpart SSp inputs. These data suggest that the SC.cl is distinguished from the SC.l by receiving convergent visual, auditory, and somatic sensorimotor information.

Quantification, represented as a proportion of labeling, illustrates the relative distribution maps of these sensory cortical areas across each SC zone (Fig. 2g; Supplementary Table 3). Polar coordinate analysis demonstrates a higher probability of pixel densities of MOs and SSp-ul inputs in the lateral angle ranges indicated by sharp peaks, segregated from the sharp peaks of VIS regions in the medial angle ranges (Fig. 2h).

### Higher-order association and prefrontal cortical projections to SC

**Fig. 3:**
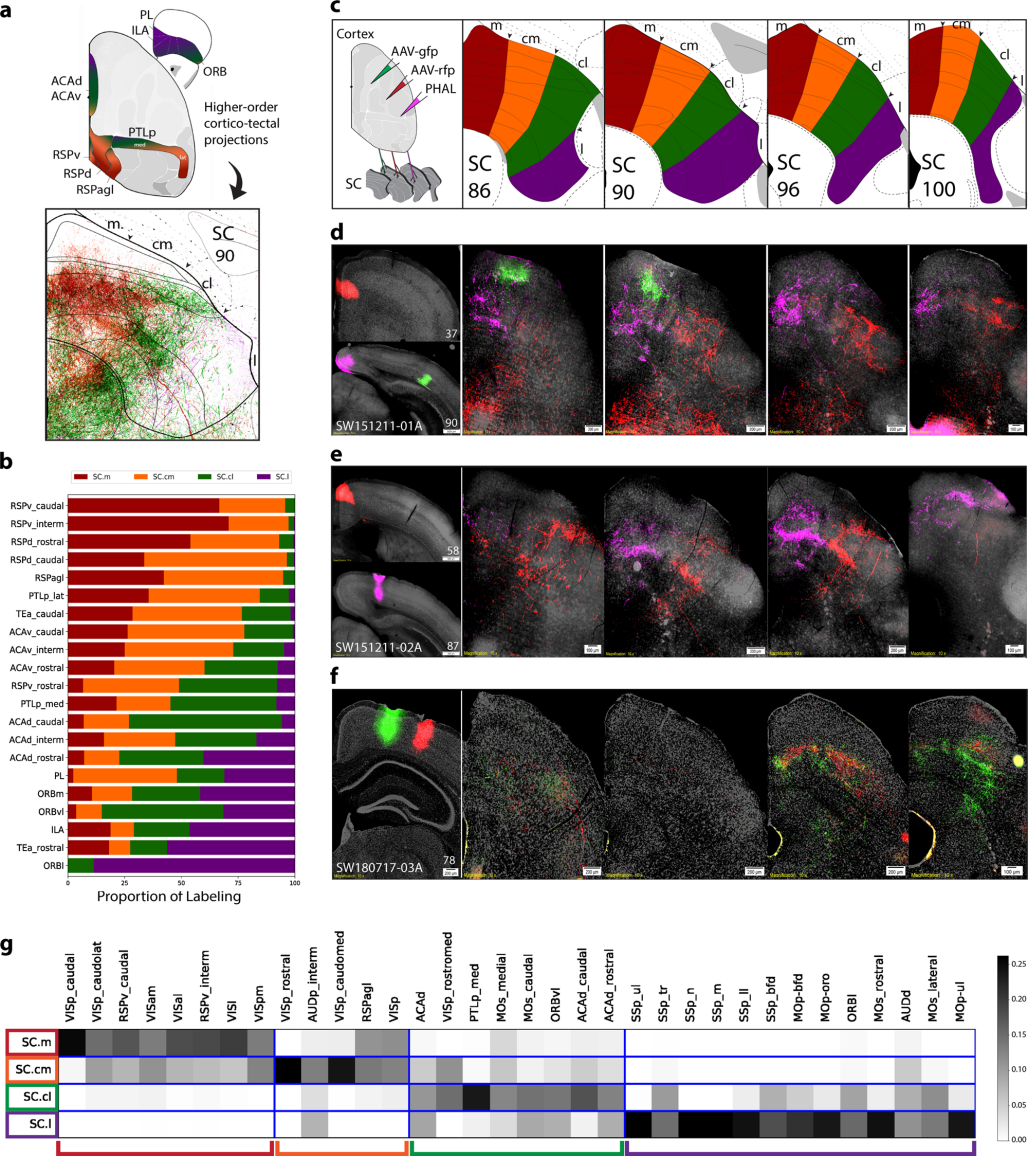
Distribution of higher-order cortical inputs across SC zones. **a** Schematic of higher-order association areas part of medial cortico-cortical subnetworks (Zingg et al., 2014). Color-coding is consistent with the SC zones, and represents the topography based on cortico-tectal projection patterns that target distinct zones. Reconstruction is composite of inputs to SC ARA 90. **b** Stacked bar chart for visualization of proportion of labeling across each SC zone (*x*-axis) from each cortical ROI (*y*-axis) for each selected ROI. Values represent proportion of pixel density for each individual ROI across each zone (*n* = 1 case per row). See Supplementary Table 4 for zone- and layer-specific values. **c** Schematic of anterograde injection strategy, and custom SC atlas levels. **d** Raw data panels. VISp, RSPv, and ACAd-rostral projections to SC. **e** ACAd-interm and VISam projections. **f** Two PTLp-medial projections projection to caudal SC levels. **g** Visualization of cortico-tectal modular communities within the SC (*n* = 34 cases). Matrices show communities identified by Louvain analysis of cortical injection ROIs (*y-*axis) and SC zones (*x*-axis). Edges are shaded according to their connectivity labeling, and boxes along the diagonal reflect modular communities identified. Legend on right side is based on the range of normalized values calculated by the Louvain algorithm. Value 0.25 represents the maximum intensity (black), and value 0 represents the minimum (white). Color-coded brackets below the matrix communities correspond to the same color codes in the SC custom atlas zones. Color-code associations: red (SC.m), orange (SC.cm), green (SC.cl), purple (SC.l).

Next, we systematically mapped cortico-tectal projections of higher-order association areas along the medial edge of the cortex, including the ACA, retrosplenial (RSP), and posterior parietal (PTLp) (Fig. 3a). These cortical areas are highly interconnected to form two medial cortico-cortical subnetworks, which transmit and integrate visual, auditory, somatic sensory, as well as spatial orientation information to the prefrontal cortex^20^. Here, we found that each of these cortical areas generate distinctive projections to different SC zones (Fig. 3b), which provide a subcortical structural basis for multimodal cortical inputs to be further integrated with visual, auditory and somatic sensorimotor information. In particular, the dorsal RSP (RSPd), agranular RSP (RSPagl), lateral PTLp, and ventral ACA (ACAv) generate dense projections to the SC.m and SC.cm (across the superficial and intermediate gray layers), while the ventral RSP (RSPv), medial PTLp, and dorsal ACA (ACAd) preferentially innervate the SC.cl. The rostral ACAd provides further inputs to the SC.l (Fig. 3c, d; Supplementary Fig. 4a). The caudal temporal association area (TEa), which is heavily connected with the visual and auditory cortical areas^20^, generates projections to the SC.cl, while the rostral TEa, sharing dense bidirectional connections with the somatic sensorimotor areas^20^, projects preferentially to the SC.l (Supplementary Table 4).

Finally, the SC also receives inputs from prefrontal areas (PFC), including the medial (ORBm), lateral (ORBl), and ventrolateral (ORBvl) orbitofrontal, prelimbic (PL) and infralimbic (ILA) (Fig. 3b; Supplementary Fig. 4b). Axonal projections arising from the ORBm, PL, and ILA are distributed across all four SC zones, while ORBvl and ORBl preferentially target the SC.cl and SC.l. Consistent with this result, the ORBvl and ORBl also share bidirectional connections with the somatic sensorimotor areas (MOs, MOp, and SSp) associated with trunk, lower limb, and whisker regions^20^. Unlike other cortical areas that generate unilateral cortico-tectal projections, the ORB areas generate bilateral projections to the SC, although its functional significance remains unclear.

### Cortico-tectal projections organized as modular communities

To visualize cortico-tectal inputs as a weighted connectivity matrix, the thresholded anterograde labels were overlapped on the layer- and zone-specific custom SC atlas templates. An implementation of the Louvain algorithm was applied to the connectivity matrix for modular community detection of cortical injection sites with their terminal distributions to common SC zones^23,24^ (see Methods). Here, we reveal that community organization is conserved in cortico-tectal subnetworks composed of distinct sensory information and higher-order associative cortical areas in the four SC zones. The connectivity matrix demonstrates that the SC constitutes four modular subnetworks differentiated by its cortical inputs (Fig. 3g). SC.m and SC.l modular networks are highly differentiated, whereas SC.cm and SC.cl are distinct, but closely related. The SC.m community contained predominantly inputs from the primary and secondary visual areas, as well as all subdivisions of the RSP, which shares bidirectional connections with all visual areas and receives spatial information through robust inputs from the dorsal subiculum through RSPv^20,25^. By contrast, the SC.l community contained the majority of inputs from somatic sensorimotor/prefrontal subnetworks from SSp, SSs, MOs, MOp, and PFC areas. Both the SC.cm and SC.cl receive highly integrated inputs from cortical areas within the medial cortico-cortical subnetworks associated with visual, auditory, and spatial orientation^20^. In particular, the SC.cm receives denser direct inputs from the visual and auditory areas, as well as several higher-order association areas, such as the ACAv, RSPd, RSPagl, and PTLp-lateral^20^. In contrast, the SC.cl preferentially receives (1) information associated with the lower central visual field; (2) direct inputs from the MOs-fef/ACAd, which is involved in controlling eye movement; (3) somatic sensorimotor inputs associated with trunk (SSp-tr); and (4) higher-order association areas receiving somatic sensorimotor inputs, such as ORBl, ORBvl, and PTLp-medial. The ORBvl also receives direct visual information^18^, thereby providing multimodal inputs to the SC.cl. This separation of communities suggests functionally distinct processing of visuospatial information in the SC.m and somatic sensorimotor information in the SC.l with more multimodal associative integration processed in the SC.cm and SC.cl.

### Brain-wide connectivity corroborates cortico-tectal networks

**Fig. 4:**
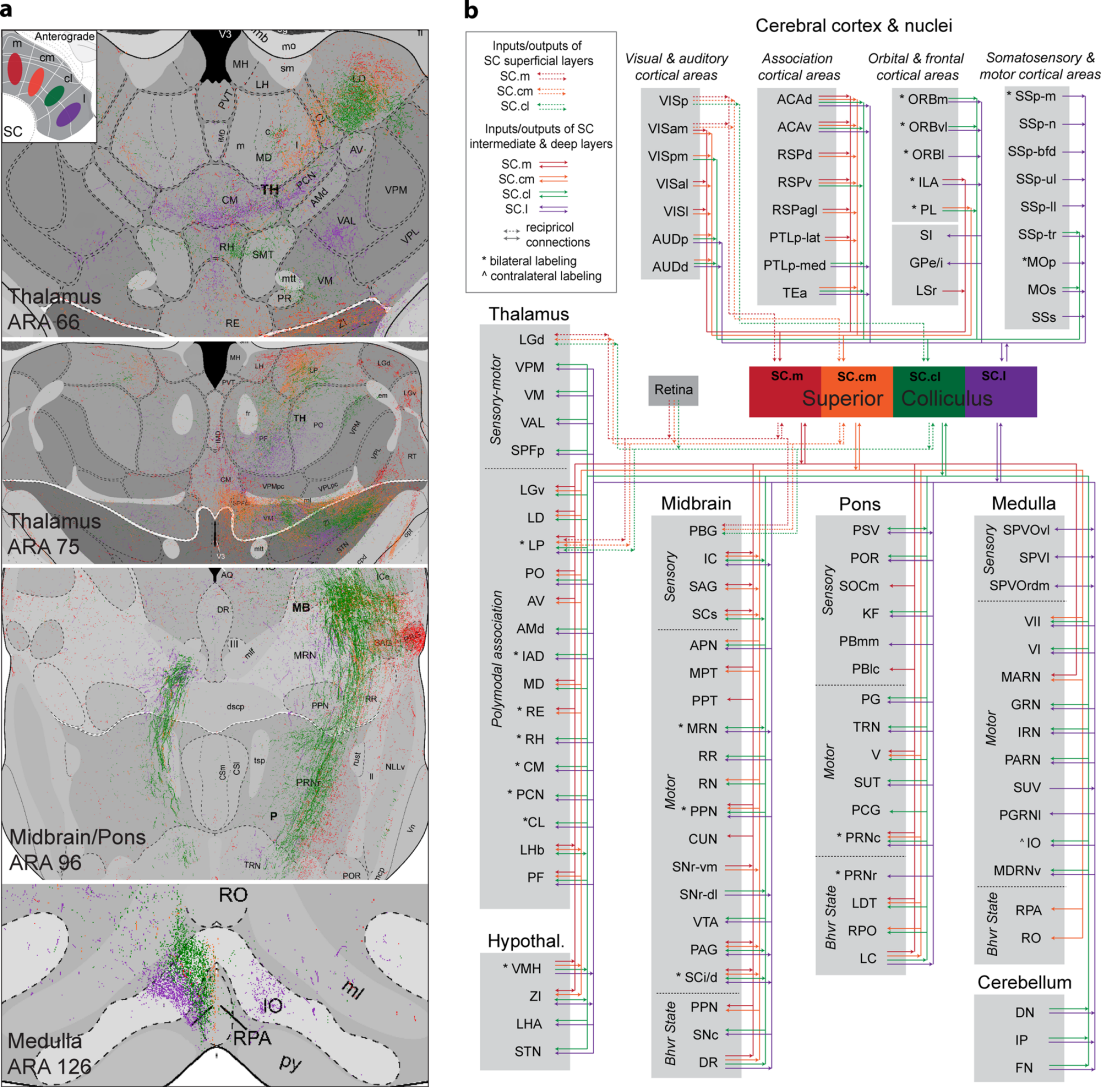
Brain-wide connectivity of SC inputs and outputs. **a** Color-coded map of anterograde projections from SC zones throughout the brain demonstrating topographic output patterns. Top left insert is a visual aid representation the anterograde injections placed in each SC zone, with colors corresponding to the same color of projections throughout the brain. See Supplementary Table 2 for injection site details. SC.m (red): SW190619-04A (PHAL), SW190619-02A (PHAL); SC.cm (orange): SW190619-02A (AAV-tdTomato); SC.cl (green): SW171010-01A (AAV-gfp), SW171010-02A (AAV-gfp); SC.l (purple): SW171010-01A (AAV-tdTomato), SW171010-01A (PHAL). **b** Unweighted wiring map of all inputs and outputs of superior colliculus zones and layers. Assembled using data from anterograde and retrograde injections placed in SC to systematically trace outputs and inputs distributed from cortex down through the hindbrain and cerebellar structures. Color-code associations: red (SC.m), orange (SC.cm), green (SC.cl), purple (SC.l). See Supplementary Table 1 for complete list of abbreviations.

After establishing the cortico-tectal projectome, we further investigated brain-wide connections of the SC in the context of their modular subnetworks. We created an anterograde projection map from each SC zone to visually summarize these spatial and topographic innervation patterns (Fig. 4a). Based on extensive anterograde and retrograde tracing data from a total of 30 injections spanning the entire SC (Supplementary Fig. 5), we assembled a complete mesoscale connectivity diagram of the mouse SC that illustrates distinctive input-output organization of all four SC zones (Fig. 4b).

#### Zone-specific connections with sensorimotor nuclei in the lower brainstem

The SC relays integrated information as motor commands via direct projections to brainstem motor and thalamic nuclei^26,27^. First, we found that the SC.l is distinguished from the other three SC zones by receiving dense inputs from the spinal trigeminal nucleus interpolar (SPVI) and ventrolateral oral part (SPVOvl)—the primary somatosensory nuclei processing orofacial and tactile information (such as the jaw fur, inside the mouth, teeth, vibrissae, nose, and adjacent eye areas^28^) (Fig. 4; Supplementary Fig. 6c), as well as the principle sensory nucleus of the trigeminal (PSV). The SC.l generates significant projections to the trigeminal motor nucleus (V) and supratrigeminal nucleus (SUT) essential in controlling jaw movements^29^ (Fig. 4; Supplementary Fig. 6b). In addition, the SC.l also generates denser projections to the intermediate reticular nucleus (IRN), parvicellular reticular nucleus (PARN) and paragigantocellular reticular nucleus (PGRNl). These reticular nuclei contain numerous premotor neurons that are organized into different central motor pattern generators in controlling orofacial rhythmic motor actions, such as chewing, licking, and swallowing^28,30^, and skilled forelimb movements^31^. Additionally, the SC.l also projects to the medial parabrachial nucleus (PBm) and Kölliker-Fuse nucleus(KF), which shares reciprocal connections with the preBötzinger complex and plays an essential role in the control of breathing, licking, and whisking^28,30,32–34^.

Quantification of output projections (Supplementary Fig. 6d) reveal that SC.l and SC.cl share several common targets, including (1) the contralateral facial nucleus (VII, predominantly its lateral part), (2) the gigantocellular reticular nucleus (GRN, predominantly to the contralateral side), which contains the highest density of reticulo-spinal neurons projecting directly to neck motor neurons to control head movements^35^ and left-right directional locomotion^36^, and (3) the midbrain reticular nucleus (MRN), which is implicated in eye-head coupling during gaze through its connection with the SC^37^. Additionally, it is important to note that both the SC.l and SC.cl receive direct inputs from deep cerebellar nuclei (DN, IP, and FN), but generate topographically arranged projections to the inferior olivary complex (IO) that relays climbing fibers to the cerebellum. Together, these cerebellar related circuits also play an integrative role in sensory-guided orofacial movements^28^. The IO-cerebellar circuits are involved in motor coordination and learning^38^, though the functional role of the triangular circuits, SC.l/SC.cl → IO → cerebellum → SC.l/SC.cl remain to be investigated.

In comparison to the SC.l, the SC.cl generates denser projections to several auditory related structures, including the inferior colliculus (all three subdivisions, ICe, ICd, and ICc, bilaterally with ipsilateral predominance), as well as the cuneiform nucleus (CUN) and nucleus sagulum (SAG)^39^. The reciprocal connections between the SC and IC are implicated in auditory defense responses^1^. The SC.m, SC.cm, and SC.cl also generate direct projections to several subdivisions of the midbrain pretectal area, including the nucleus of the posterior commissure (NPC), olivary pretectal nucleus (OP), nucleus of the optic tract (NOT), nucleus of the posterior commissure (NPC), as well as the posterior (PPT) and medial pretectal nuclei (MPT). The pretectum receives direct binocular input from photosensitive ganglion cells in the retina and is primarily involved in mediating nonconscious behavioral responses to acute changes in light such as the pupillary light and optokinetic reflexes^40^. All four SC zones provide significant projections to the anterior pretectal nucleus (APN), which is implicated in nociceptive guided visual motor actions and the pathogenesis of central pain syndrome^41^. The SC.m projects directly to the parabigeminal nucleus (PBG) and this SC → PBG pathway has been implicated in detecting looming objects and triggering fear responses^42^. Finally, each SC zone projects densely to the periaqueductal gray (PAG, presumably with topographic projection patterns), which plays a critical role in coordinating somatic and autonomic reactions in defensive responses and is a critical region in driving motivation for hunting and foraging behaviors^1,43,44^. It is important to note that the four SC zones share extensive connections with brainstem monoaminergic cell groups with different preferences. Each group receives inputs from the locus coeruleus (LC, noradrenergic) and dorsal raphe (DR, serotonergic). All SC zones project to the midbrain dopaminergic groups in the SNc and VTA, and PPN (cholinergic), with the SC.m projecting to the lateral dorsal tegmental area (LDT, cholinergic). The SC.cm also generates specific projections to the medullar raphe nuclei (DR, RPA, RO, and RPO) (Supplementary Fig. 6d).

#### Hypothalamic interactions with SC

**Fig. 5:**
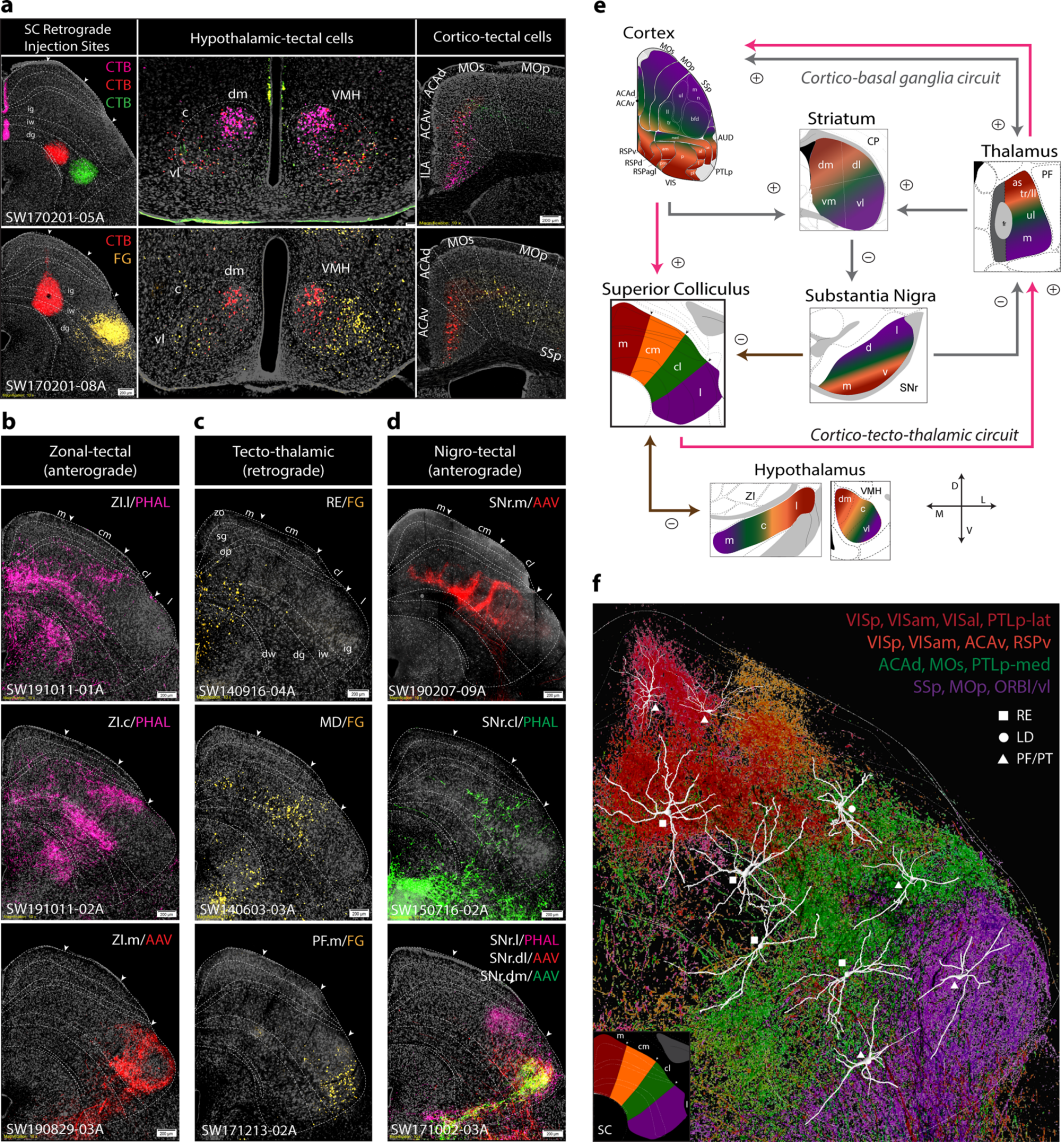
Topographic organization of brain-wide inputs and outputs of SC zones. **a** Raw data labeling from two separate cases of retrograde injections into the SC. Top row: CTB (pink) injection into SC.m, CTB (red) injection into SC.cl, and CTB (green) injection into SC.l. Cells are topographically retrogradely labeled in VMH domains and cortex. Bottom row: CTB (red) injection in SC.cm, and FG injection in SC.l. **b** Raw connectivity data in SC (all at ARA 90) from three separate anterograde injections in the ZI. The ZI.l (lateral part) targets SC.m/cm; the ZI.c (center part) targets SC.cl, and the ZI.m (medial part) targets SC.l. **c** Raw connectivity data in SC (all at ARA 90) from three separate FG injections in different thalamic nuclei, RE (top), MD (middle) or PF.m (bottom), and the respective retrogradely-labeled thalamic projections neurons, respectively, in SC.m/cm, SC.cl, or SC.l. Cells were distributed heterogeneously across superficial, intermediate and deep layers, but clustered topographically and preferentially in distinct zones. Scale bar 200 µm in SC panels. **d** Labeling produced from anterograde injections into distinct SNr domains project to SC. **e** Topographic organization of mouse SC connectivity. Directional cross at bottom left. The four zones of the SC are connected either uni- or bi-directionally with cortex, PF, ZI, VMH, and SNr. Plus signs and minus signs indicate excitatory or inhibitory projections, respectively. **f** Dendrites from LD-, RE-, and PF/PT-projecting SC neurons were reconstructed to visualize differences and dendritic arborizations across SC layers and zones. Examples of reconstructed neurons were overlapped onto reconstructions of cortico-tectal projections on the SC atlas. This facilitates analysis of spatial correlation between cortical projection fields and dendritic fields of SC-projection neurons. Color-code associations: red (SC.m), orange (SC.cm), green (SC.cl), purple (SC.l). See Supplementary Table 1 for complete list of abbreviations.

The SC zones also receive topographic inputs from hypothalamic structures, including the zona incerta (ZI) and ventromedial nucleus of hypothalamus (VMH)^45,46^. The SC.cl and SC.l intermediate and deep layers receive dense inputs from the ventrolateral part of the ventromedial hypothalamus (VMH.vl), known to be involved in approach, appetitive, reproductive and social attack responses^47,48^. Triple retrograde injections into the SC.m-ig (CTb-647), SC.cl-dg (CTb-555), and SC.l-dg (CTb-488) reveal that the SC.m receives dense inputs primarily from the VMH.dm, whereas the SC.cl and SC.l receive more inputs from the VMH.vl. Another experiment comparing the SC.cm (CTb-555) and SC.l-ig (FG) revealed the SC.cm receives input from the VMH.dm/c, and the SC.l receives input from VMH.vl. (Fig. 5a). Interestingly, we also found that the SC.cl and SC.m generate significant projections to the anterior hypothalamic nucleus (AHN). The AHN and VHMdm share reciprocal connections and are two essential nodes of the hypothalamic defensive network^49^.

The output patterns of the ZI to SC support the topographic organization of medial visuomotor and lateral somatic sensorimotor subnetworks. Anterograde injections into (1) the lateral ZI project to SC.m and SC.cm, (2) central ZI projects to SC.cm and SC.cl, and (3) medial ZI projects to SC.l (Fig. 5b). Interestingly, lateral ZI → SC.m/cm innervate superficial and intermediate layers, further supporting the intrinsic alignment of visuomotor subnetworks that also align with our identified cortico-tectal communities. These findings are congruent with functional networks implicating the lateral SC in appetitive and approach behaviors^19,50^.

#### Connectivity within cortico-tecto-thalamic subnetworks

Thus far, we identified that each SC zone receives convergent inputs from a specific set of cortical areas and displays distinguishable connections with sensorimotor nuclei in the brainstem as described above. Numerous studies have described detailed neuronal connections of the SC with the thalamus^51,52^. Here, we further demonstrate how each of these newly defined SC zones sends projections back to specific thalamic nuclei that send ascending projections to the same cortical areas that generate direct projections to the SC, thus completing the loop within the cortico-tecto-thalamic subnetwork. The SC.m and SC.cm share reciprocal connections with the dorsal lateral geniculate nucleus (LGd) and lateroposterior nucleus (LP), and generate projections to the ventral lateral geniculate nucleus (LGv), laterodorsal nucleus (LD), posterior nucleus (PO), nucleus of reuniens (RE), anteroventral nucleus (AV), and parafascicular nucleus (PF) (Fig. 4a). As shown in Fig. 4a, color-coded axonal terminals from the SC.m and SC.cm in the LP and LD are topographically arranged. Injections of two visual-related thalamic nuclei, the LP and LD, resulted in dense labeling in the SC.m and SC.cm, but with different laminar specificities (Supplementary Fig. 3d). LP-projecting neurons are distributed specifically in the superficial gray layer (sg), while LD-projecting neurons are distributed primarily in the intermediate gray layer (ig). Our results also revealed that the SC.m and SC.cm (mostly intermediate and deep layers) contain dense thalamic projecting neurons that target the RE (Fig. 5c). The LP, LD, and RE share massive bidirectional connections with the visual areas and several high order association areas (such as the ACA, RSP, and PTLp) that innervate the SC.m and SC.cm. Together, these structures are organized into a cortico-tecto-thalamic network that process  and integrate  visual, auditory, and spatial orientation information to regulate motor outputs associated with goal-directed behaviors.

In parallel, we found the SC.cl contained a discrete thalamic projecting neuron that targeted the LD, PO, and lateral and center parts of the mediodorsal thalamic nucleus (MDl, MDc) (Fig. 5c). The MDl shares dense bidirectional connections with the ACAd and MOs-fef, which generate direct projections to the SC.cl. Altogether, these structures are organized into another distinctive loop as follows: cortico(ACAd and MOs)-tecto(SC.cl)-thalamo(MDl)-cortical. The SC.l (and its adjacent SC.cl) contains thalamic projecting neurons that target several somatic sensorimotor-related thalamic nuclei, such as the ventrolateral parafascicular nucleus mouth region (PF.m)^53^, centromedial (CM), ventral anterolateral nucleus (VAL), the caudal ventromedial nucleus (VM), and paracentral nucleus (PCN) (Fig. 4; Supplementary Fig. 3d), following a topographically organized somatotopic order. In particular, the rostral SC.l projects primarily to the PF.m, which also shares massive bidirectional connections with the somatic sensorimotor areas (SSp and MOp) associated with the mouth and upper limb^53^. In parallel, the caudal levels of SC.l and SC.cl send selective projections to the dorsolateral PF and rostral VM, which also share bidirectional connections also with the somatic sensorimotor cortical areas associated with the trunk and lower limb. To our knowledge, these somatotopically organized cortico-tecto-thalamic loops have not been reported before (Fig. 5e).

Finally, to test the anatomical basis for how SC neurons integrate inputs from different sources, we characterized dendritic morphologies of SC neurons. Representative neurons in each zone were labeled via a G-deleted-rabies injection in the LD (for SC.m/cm), RE (for SC.m/cm/cl), and dorsal PF (for SC.cm/cl/l) (Supplementary Fig. 7). The large injection into the dorsal PF also partially infected neurons in the midbrain pretectal region (MPT), which retrogradely-labeled cells in the superficial layers of SC.m (we thus refer to this injection as PF/MPT). Reconstructions of rabies-labeled SC neurons projecting to RE, LD, and PF/MPT allow us to analyze the spatial correlation between cortical projection fields and dendritic fields^54,55^ of SC-projection neurons^56,57^ (Fig. 5f). Here, reconstructed neurons are superimposed above their corresponding SC zones and receive inputs from visual-related cortical areas (VIS, RSP, and PTLp), and reconstructions of PF/MPT-projecting neurons in SC.cl/SC.l zones overlap with inputs from somatic sensorimotor subnetworks (see Figs. 2c, 3d, e). We found significant distinctions in the morphometric features of width, number of bifurcations, branch path length, and contraction (Supplementary Fig. 7c)^58,59^. Notably, based on the width (overall arbor size) in each zone, cells projecting from SC.m superficial layers had smaller dendritic fields, and cells from intermediate and deep layers of SC.l were larger as they transitioned through intermediary sizes in SC.cm and SC.cl, respectively. This size trend in the zones is further modulated by a second trend displayed by the projection target, whereby the LD-projecting cells have the smallest arbors, and RE-projecting cells in the SC.cl and PF/MPT-projecting cells in the SC.l have the largest arbors. Larger dendritic fields imply the ability to integrate from a broader array of incoming fiber pathways, whereas a narrower arbor reflects a selectively focused input tuning^48^. These neurons exhibited heterogeneous morphological properties consistent with the rodent cell types that may contribute to distinct behaviors^42,48,60^. It is possible that downstream-projecting SC neurons display varying morphological features than ascending thalamus-projecting SC neurons^42^; nevertheless, our results suggest that functional analyses of SC neurons involving motor, sensory, and cognitive behaviors can also be addressed in the context of inputs and outputs from distinct modular networks. Several rodent studies provide entry into genetic and cell-type-specific investigations of functional SC networks^48,61,62^, though more studies are needed to characterize how multimodal cortical and subcortical information is integrated at single neuron resolution. All neuronal morphologies reconstructed in this study along with detailed experimental metadata are cataloged into a digital library, accessible on www.NeuroMorpho.Org^63^.

### Interactions between cortico-tectal and basal ganglia subnetworks

**Fig. 6:**
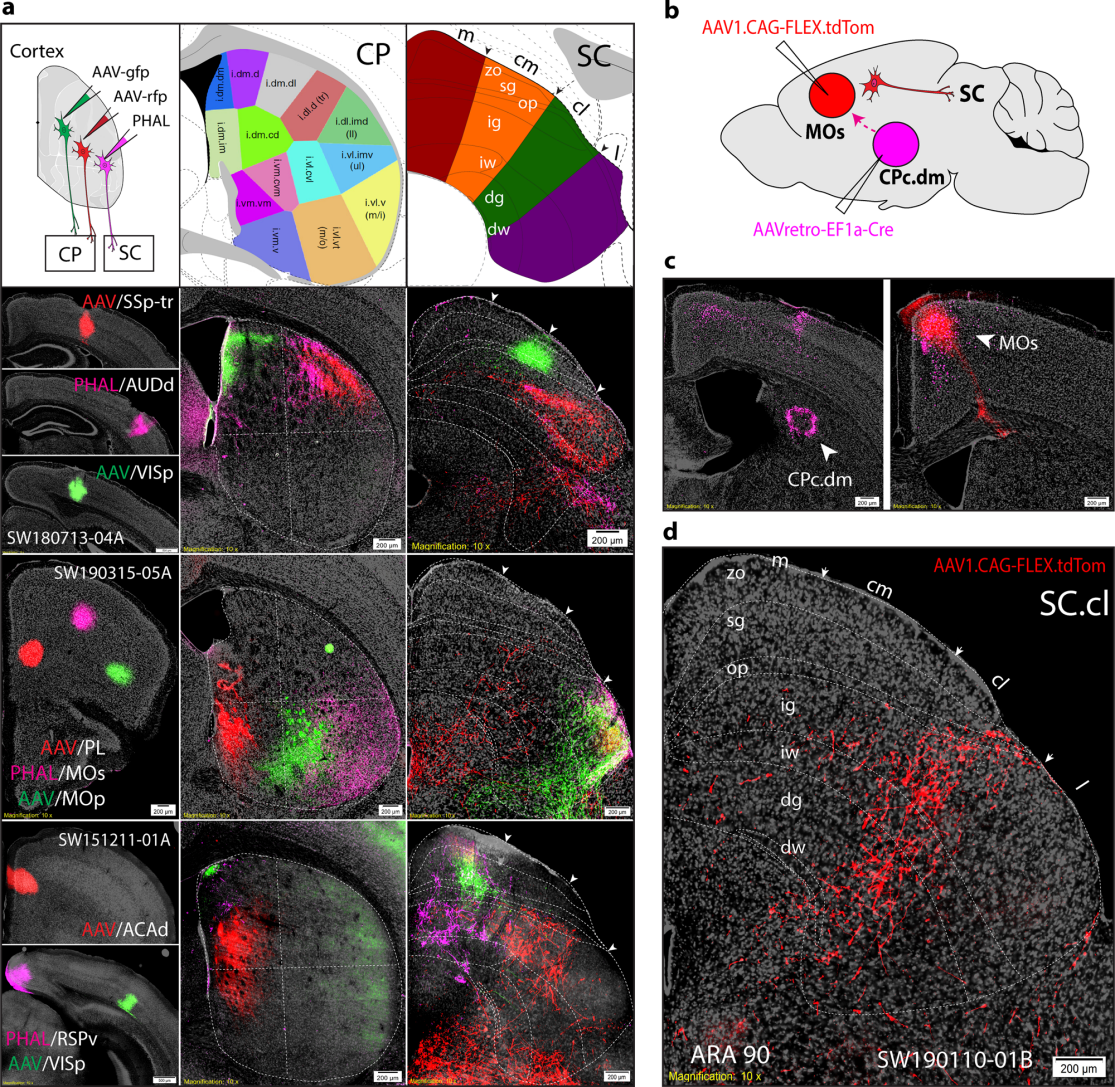
Cortico-striatal and cortico-tectal have conserved topography. Within the cortex, somatic sensorimotor areas are each organized into distinct highly interconnected subnetworks that integrate the visual field, somatotopic body map and higher-order areas which then project subcortically to the caudoputamen (CP) into distinct domains^19,20^. Each cortical area sends parallel descending projections to SC zones and CP domains. **a** Three separate triple anterograde cases into different cortical areas send topographic projections to corresponding caudate putamen (CP) domains and SC zones. **b** Injection site schematic of combinatorial tract-tracing method using AAVretro-Cre injection (in CPc.d at ARA 57) and Cre-dependent AAV-mCherry (in MOs at ARA 47). **c** Injection sites in CPc.dm and MOs within the same mouse brain (SW190110-01B). **d** Red anterograde labeling demonstrates a subset of MOs cortical projecting neurons (presumably pyramidal tract projection neurons or PT neurons) generate collateral projections specifically to the SC.cl zone and CPc.dm. Scale bars are 200 µm. Color-code associations: red (SC.m), orange (SC.cm), green (SC.cl), purple (SC.l). SC superior colliculus, SC layers: zo zonal, sg superficial gray; op optic, ig intermediate gray, iw intermediate white, dg deep gray, dw deep white.

The interactions between the cortico-tectal and basal ganglia system were noted in previous work, but have not been systematically investigated^26,51,64,65^. First, the comprehensive cortico-tectal projection map, together with our previous cortico-striatal projection map^21^, allows us to identify a one-to-one correspondence of cortical axonal terminal fields in the CP and SC arising from individual cortical areas (Fig. 6a). This provides an anatomical framework for understanding functional correlations between these two systems in regulating motor behavior. In fact, the same cortical projection neurons generate collateral projections to innervate both targets^1,66^. As an example, a subset of MOs/ACAd cortical projecting neurons (presumably pyramidal tract projection neurons or PT neurons) generates collateral projections to both the SC.cl and dorsal domain of caudal CP (CPc.d) (Fig. 6b–d). Thus, in addition to direct cortico-tectal projections, cortical information can also reach the SC indirectly through a multi-synaptic, cortico-basal ganglia-tectal projection pathway.

Each SC zone harbors distinct thalamic projecting neurons, and receives direct convergent inputs from functionally correlated cortical areas (as aforementioned) as well as inputs from specific domains within the reticular part of the substantia nigra (SNr), the motor output node of the basal ganglia subnetworks (see Fig. 5d, e). Our recent work demonstrated that the SNr is subdivided into 14 different domains based on their convergent and divergent inputs from 30 different striatal domains, each of which in turn receive a specific set of functional relevant cortical inputs^21,53^. The newly defined medial SNr domain (SNr.m), which receives convergent inputs from the dorsomedial striatum that in turn receives convergent inputs from the visual cortical areas, ACA, RSP, and PTLp^20,53^, generate dense projections to the SC.m, SC.cm, as well as SC.cl (Fig. 5c) that align with patterns from cortico-tectal projections from these high order association areas (see Fig. 3a). In contrast, the centrolateral domain of the SNr (SNr.cl), which receives the densest inputs from the dorsolateral striatal domains that are innervated by the MOp and SSp trunk and lower limb domains, project specifically to the SC.cl. Finally, the SC.l receives dense inputs from the SNr domain (Fig. 5c) that is specifically innervated by the ventrolateral CP domains, which receive convergent somatic sensorimotor cortical inputs associated with orofacial and upper limb (Fig. 6a).

**Fig. 7:**
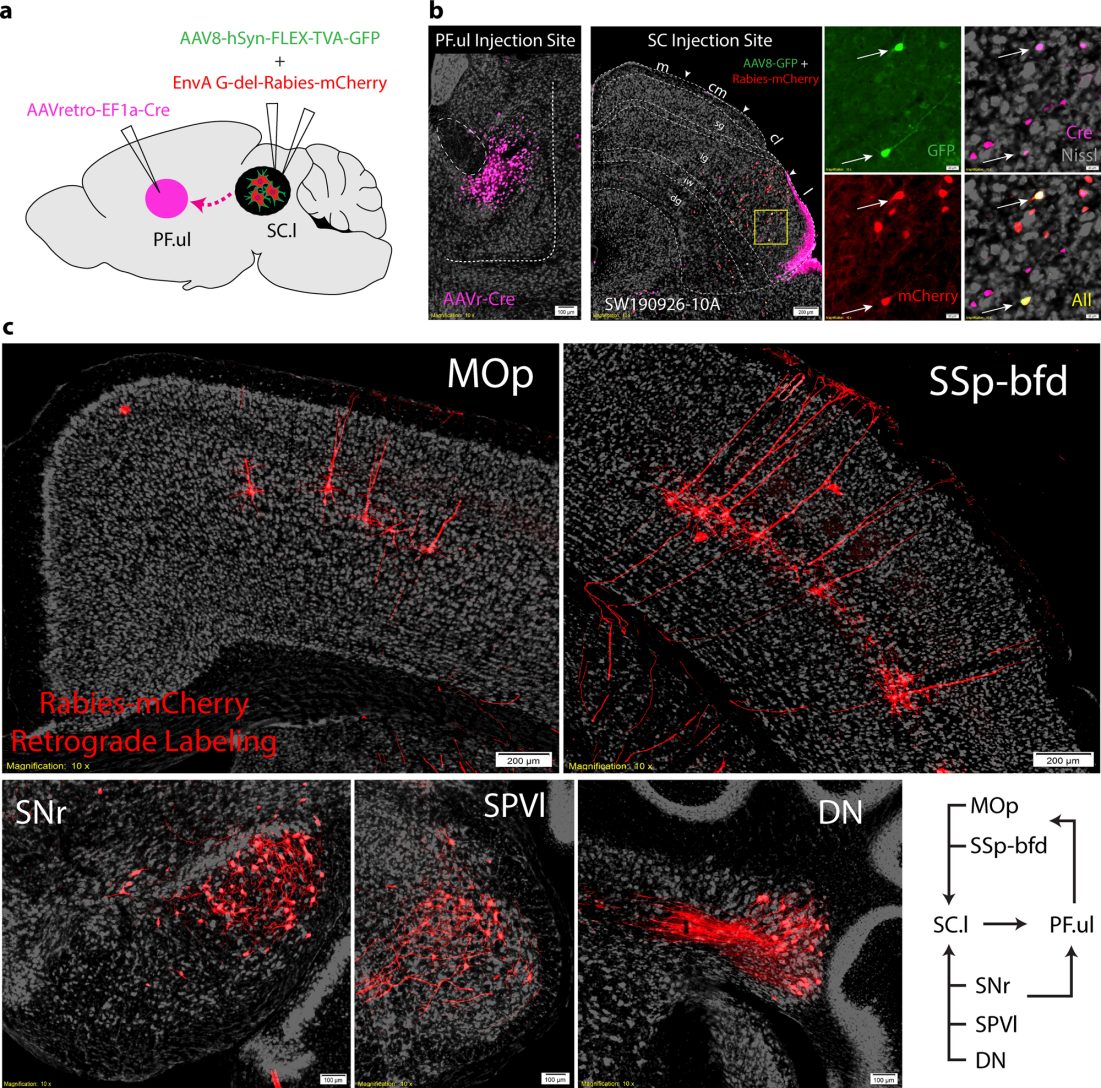
Upper-limb/orofacial subnetworks in the SC.cl and SC.l. **a** TRIO-tracing strategy targeting AAVretro-EF1a-Cre in PF.ul (PF upper limb), and rabies virus (AAV8-hSyn-FLEX-TVA-GFP) and Cre-dependent helper virus (EnvA G-del-Rabies-mCherry) in SC.l reveal mono-synaptic inputs to the PF-projecting neurons in the SC.l. **b** Raw images of injection sites in PF.ul, and SC.l with zoomed in panels of triple labels starter cells in SC (case SW190926-10A). **c** Rabies retrograde labeling in somatic sensorimotor cortical areas MOp and SSp-bfd. Cells labeled in the dorsal and central lateral substantia nigra reticulata (SNr), the interpolar part of the spinal nucleus of the trigeminal (SPVI) and dentate nucleus (DN) of the cerebellum. Bottom right corner is a summary diagram showing that thalamic (PF) projecting neurons in SC.l receive convergent inputs from the somatic sensorimotor cortical areas (MOp, SSp), subset of the basal ganglia (SNr), somatic sensory nuclei (i.e., SPVI), and deep cerebellar nuclei (i.e., DN).

In parallel, we demonstrate a refined network of organization of parallel channels relayed through the cortico-tecto-thalamic circuit. This is most evident in somatotopic maps from SSp and MOp to SC.cl and SC.l zones. The four SC zones project to at least four domains within the PF, where SC.cl/SC.l → PF.m/ul, SC.cm/cl → PF.tr/ll, and SC.m → PF.as (Fig. 5e). Output projections from SNr domains also target the same somatotopic zone in SC as well as the corresponding domain in PF. To show the subnetwork interactions of this cortico-tecto-thalamic pathway and reveal mono-synaptic inputs to the PF-projecting neurons in the SC.l, we used a TRIO (tracing the relationship between input and output)-tracing strategy targeting AAVretro-Cre in dorsomedial PF, and rabies virus and Cre-dependent helper virus in SC.l^67^ (Fig. 7a, b). As expected, rabies-labeled cells that target PF-projecting SC.l neurons were found in SSp-ul, SSp-m, SSs, and MOp-oro cortical areas. Additionally, neurons were labeled in the dorsolateral SNr, deep cerebellar nuclei (DN) and the spinal nucleus of the trigeminal interpolar part (SPVI) and ventrolateral oral part (SPVOvl) associated with processing orofacial and tactile information^68^ (Fig. 7c). These data provide evidence to suggest the SC.l receives convergent inputs from functionally correlated cortical areas, subsets of cortico-basal ganglia (i.e., dorsolateral SNr), deep cerebellar nuclei and brainstem sensorimotor nuclei associated with hand-to-mouth coordination behaviors. In turn, the SC.l sends projections to the PF, through which integrated somatic information is sent back to the corresponding cortical areas. Finally, we observed that all four SC zones directly project to the SNc and VTA, which presumably generate dopaminergic projections back to the striatum and cortex. The SC.l also generates direct projections back to multiple components of the basal ganglia, including the GPe, GPi, and STN^26,27,65^. Additional genetic and trans-synaptic experiments should be pursued to further validate the connections described.

## Discussion

Numerous anatomical and functional studies have demonstrated that the SC plays a critical role in integrating external environment information and determining the whereabouts of specific events, such as potential prey or predators, to guide animals’ goal-directed behaviors^69^. The SC receives extensive cortical inputs to facilitate various functions including spatial attention, navigation, defense, and decision-making^1,14,15,70,71^. These connections are often divided into two broad functional modules along the medial and lateral halves^19,72^, yet there is more complexity underlying its organization that has not been previously shown. Thus, not only have we refined the SC to four anatomically relevant subdivisions, but we have also revealed they are organized as distinct subnetworks throughout the brain. Here, we generated a comprehensive anatomical map of the mouse SC revealing parallel subnetworks correlated with functional specializations. In particular, we systematically characterized all cortico-tectal pathways based on the convergence and divergence of cortical fiber terminations across the SC to create the mouse cortico-tectal projectome. We identified four zone-specific delineations from medial to lateral in the SC using a combined connectivity and computational neuroanatomic approach: SC.m, SC.cm, SC.cl, and SC.l (Fig. 1). Each zone also shares distinguished connectivity patterns with different sensorimotor nuclei in the lower brainstem, hypothalamus and thalamus. Together, they assemble four parallel neural networks in controlling different sensory integration and motor actions.

This work provides an important conceptual understanding of how cortical and subcortical subnetworks are superimposed in the SC to guide functionally distinct responses. For example, for the first time, we characterized distinct connectivity features of the SC.l and SC.cl, which were previously collectively grouped into the lateral SC. In regard to somatotopic order, their connectivity patterns clearly reveal a somatotopic map with the rostral SC.l associated with orofacial and upper limb, while the caudal SC.l and SC.cl associated with lower limb, and body trunk (Supplementary Fig. 3b). This rough rostro-caudal somatotopic order is similar to that in the cortex^20^, but distinguished from that in the dorsal striatum^21^, GPe and SNr^53^. The SC.l sits in a unique interface to integrate somatic sensorimotor information from brainstem sensory nuclei (i.e., SPVO, SPVI), cortical somatic sensorimotor areas, and cerebellum. In turn, the SC.l sends this information to the cortex through the tecto-thalamo-cortical pathway on one hand, and project to the motor nuclei in the lower brainstem (presumably in regulating behavior). Specifically, we found that the SC.l receives predominantly somatotopic sensory information from the SPVI and SPVOvl, and somatic sensorimotor cortical areas associated with orofacial and upper-limb area and projects to the brainstem reticular formation in controlling orofacial movements. In contrast, the SC.cl (1) receives convergent cortical inputs associated with the lower visual field, and somatosensory inputs associated with the upper body trunk, (2) receives inputs from MOs frontal-eye-field that is involved in controlling eye movements, and (3) projects to the GRN and other structures that coordinate head movements. These data suggest that the SC.cl plays a critical role in controlling eye-head coupling and orienting movements during exploratory behavior (i.e., prey capture)^33^, while the SC.l is involved in controlling orofacial and upper-limb movement in the consummatory phase of behavior (e.g., biting, chewing).

Next, in regard to the visual field organization in SC.m, SC.cm, and SC.cl, our results show that each zone receives dense visual inputs, but with different preferences corresponding to the upper central, upper peripheral, and lower portions of the visual field. We found that the SC.m receives inputs from the VISp-caudal  and caudolateral,  while the SC.cm receives inputs from the VISp-rostral and caudomedial. The SC.m receive denser inputs from the RSPv (which receives denser spatial information from the dorsal subiculum^20^, suggesting more visuospatial integration, while SC.cm receives denser inputs from AUDp, suggesting more visuoauditory integration. The SC.m and SC.cm share connectivity with most cortical and subcortical structures associated with visual and auditory integration and visuomotor responses to perceive, localize, and recognize objects in the outside world. In addition, both the SC.cm and SC.m project to the APN, which receives nociceptive inputs and is implicated in central pain syndrome^41^.

**Fig. 8:**
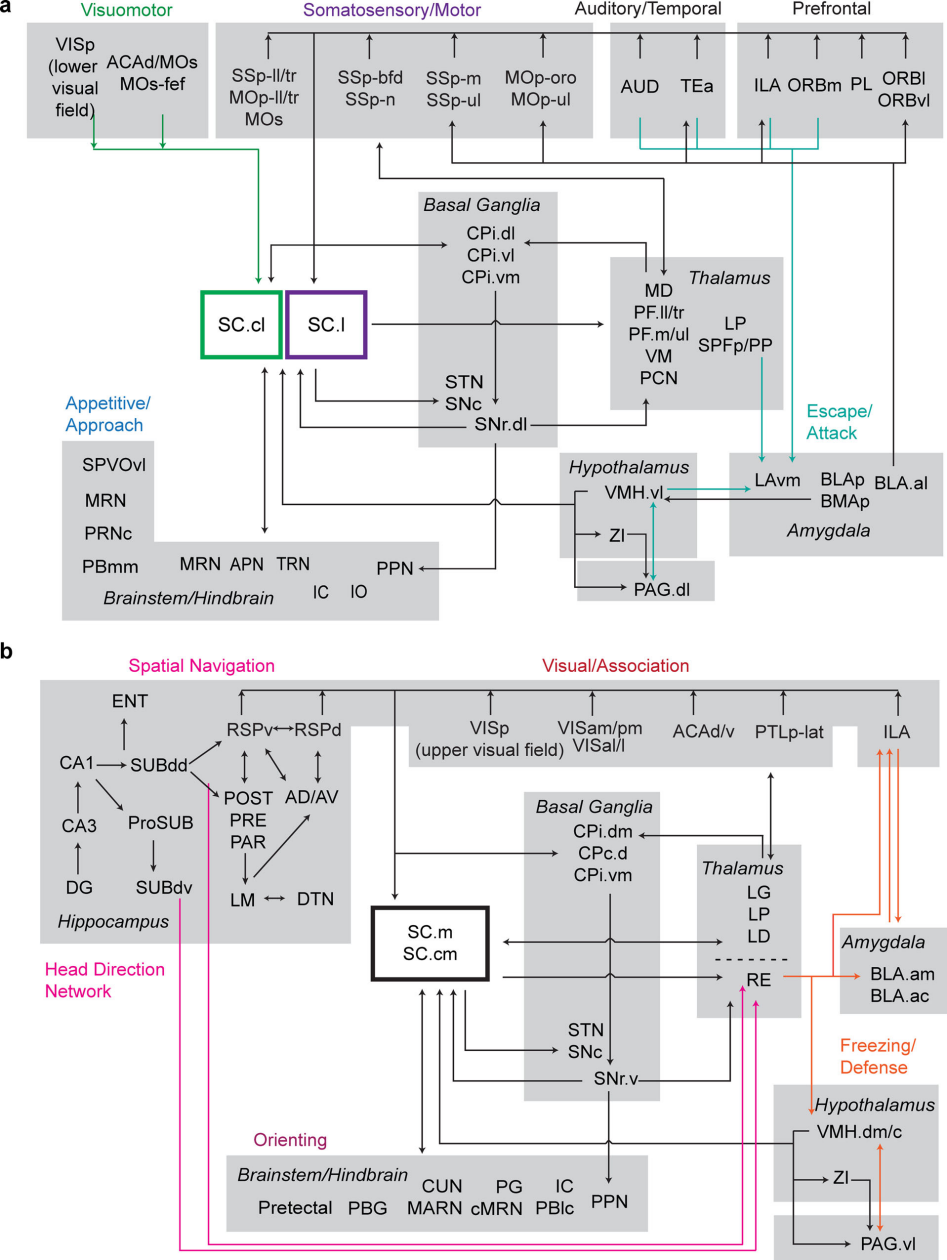
Subnetwork organization of SC zones. **a** Integration of SC.cl and SC.l zones in visuomotor, somatic sensorimotor, escape and approach subnetworks. SC.cl/l neurons receive inputs from MO/SS (orofacial, barrel field, upper limb) cortices, motor-related domains from thalamic nuclei (PF.mouth, PF.upper limb, VM, PCN), sexual approach behavior (VMH.vl) and orofacial/upper-limb brainstem and cerebellar inputs. This input provides evidence for SC.cl/l zones as distinct somatic sensorimotor subnetworks that mediate a subset of behaviors distinct from the more visually integrated range of behaviors mediated through SC.m/cm. Our network analysis reveals the segregation of ventromedial prefrontal cortex (vmPFC) outputs from ORB and ILA regions to functionally specific SC.cl/l zones. These prefrontal regions also project densely to VMH, ZI and PAG, with BLA sharing bilateral connections with ILA^81^). **b** SC.m and SC.cm integration of visual information with spatial-related and head-direction hippocampal networks^25^, attention/orienting regions and freezing/defense regions. See Supplementary Table 1 for complete abbreviation list.

Furthermore, we found each of the four zones are associated with distinct parallel cortico-tecto-thalamic subnetworks, and with cortico-basal ganglia-thalamic subnetworks (Fig. 5e). The continuity in topographic organization throughout structures within the basal ganglia system and thalamic nuclei with the SC zones suggests a grand orchestration of compartmentalized integration to facilitate coordinated behaviors. Altogether, the complete wiring diagram of the SC enables us to generate functional networks (Fig. 8) associated with several testable functional hypotheses, as elaborated below.

### Subnetworks implicated in approach/appetitive behaviors

The cortico-tectal lateral zones have functional implications in somatic sensorimotor movements that mediate appetitive and approach related behaviors^19,73,74^ (Fig. 8a). The sparse projections arising from ORBm/vl → SC.cl overlap with ACAd/MOs-fef → SC.cl inputs that target the lower nasal and temporal regions of the visual field represented in SC.cl, which is medially adjacent to the somatic sensorimotor subnetworks that facilitate spatial orientation near the ground. This suggests that vmPFC → SC provides prefrontal top-down input to command emotional and decision-making related responses. The SC receives cortical information both directly through the cortico-tectal projection pathway and indirectly through the multi-synaptic cortico-striato-nigra-tectal projection pathway. The lateral PFC projections from ORBl → SC.l inputs align with barrel field and oropharyngeal subnetworks in SC.l, thereby guiding decision-making about relevant stimuli proximal to the ground^73,75^. The combination of these limbic system inputs may facilitate the integration of valence and motivated emotional responses in social and appetitive behaviors^49^, though functional experiments are required to test this hypothesis. Our anatomical findings support that the SC.cl is involved in eye-head coordination during approach/appetite behavior, including pray capturing, while the SC.l is involved in sensorimotor actions of orofacial movement during the consummatory phase (such as chewing, licking, and biting).

### Subnetworks implicated in defensive/aggressive behaviors

The cortico-tecto-thalamic subnetwork is directly and indirectly connected with hypothalamic and amygdalar structures that may coordinate the activation of fear-related circuitry^49,76,77^. Within the medial cortico-cortical subnetworks, ORB is interconnected with ACAv, ACAd/MOs, and PTLp-med (internal body map), which all project to the SC.cl/l zones, suggesting a coordination of motor outputs for the whole body to prompt escape and attack reactions (Fig. 8). Notably, the SC.m and SC.cm deep layers also receive dense input from the dorsomedial part of the VMH (VMH.dm/c), known to evoke innate defensive responses and aggressive responses to predators^42,78,79^. Moreover, these medial SC zones send outputs to the RE, CM, and IAM, which have been shown to be activated during arousal and the presence of visual threats^52,80^. Our results reveal an ILA → SC.m/l pathway that is consistent with the role of ILA in mediating fear extinction, risk assessment and evaluation to threat situations (particularly to looming stimuli/predators)^19,50,74,81^.

### Subnetworks implicated in navigation and goal-oriented behaviors

As described, RSP, PTLp-lat, and VIS prominently align in the medial zones SC.m and SC.cm (and partially in SC.cl) that process distinct representations of visual information from the upper peripheral and central regions of the visual field^48,55^ (Fig. 8b). The RSP → SC presumably contributes to mediating spatial orientation and navigation, particularly through head-direction cells in RSPv interacting with the CA1dr/subiculum hippocampal network^25,62,82^. The SC.m/cm, in turn, projects to RE in the thalamus, which also contains head-direction-sensitive cells^83^. The ACA is implicated in guiding response selection based on the value in context, richness, and memory of an environment during exploratory and foraging behavior^74^. As environments vary during navigation, the overlapping inputs from ACA → SC contribute to facilitating attention during coordinated exploratory and evaluative response strategies. Our findings of the visuomotor subnetwork in SC.cl further support this zone as an analogous point of convergence for frontal-eye-field orienting behaviors in primates and cats^2^. In particular, the SC.cl further projects to the MRN and GRN, which are involved in coordinating locomotive and attentive visual behaviors^84,85^. As such, the widespread projections from ACAd/v to SC.cm, SC.cl and SC.l may provide information regarding the contextual value of the environment that integrates with multisensory SC neurons processing input from virtually the entire visual, auditory, and somatotopic fields. Cells in the SC.cl-ig aligned with acetylcholinesterase-rich patches also project strongly to the MD thalamic nucleus, which is involved in frontal-eye-field processing^77,86,87^. By incorporating value-defined inputs across the SC, we can better understand the role of distinct SC zones in commanding eye movements and goal-oriented motor behaviors^88^.

Overall, these results refine previous studies observing sensory topographic distribution patterns within the SC and provide a comprehensive understanding of how all higher-order cortical inputs are integrated. To the best of our knowledge, this map of the cortico-tectal projectome is the first in the mouse model and will thus serve as a useful cross-species reference^17,18,89^. We have shown the mouse SC can be subdivided into four columnar zones with distinct connectivity that correlate with functional subnetworks. For each zone, we characterized the input-output organization and provided working hypotheses to explore their functional implications. This study advances our understanding of SC organization within brain-wide subnetworks. In humans, the SC is hypothesized to be a major locus of interest for potential therapeutic targets in treating hyper-responsivity and distractibility in attention-deficit hyperactivity disorders^8–11^. Understanding the neural networks of attention-related visual behaviors in mouse models has clinical significance in examining aberrant connections of neurodevelopmental disorders^7^. Ultimately, the cortico-tectal projectome provides an anatomical framework for understanding how individual SC cell types integrate convergent input from multiple brain areas and functional modalities for the control of attention and other goal-directed behaviors.

## Methods

### Mouse connectome project methodology

Anatomical tracer data was generated as part of the Mouse Connectome Project (MCP). We systematically and carefully mapped neuronal connectivity of each cortical structure to determine their input connectivity to the superior colliculus (SC) (for complete injection site list, see Supplementary Table 2). We used multiple fluorescent tracing strategies with a combination of classic tract-tracing and viral tracing methods. First, we used a triple anterograde tracing approach with individual injections of PHAL and GFP- and tdTomato- expressing adeno-associated viruses (AAV). Additionally, to investigate the convergence or divergence of axonal fiber pathways either into or out of the SC, we used a double co-injection method that introduces two different tracer cocktails each containing one anterograde and one retrograde tracer to simultaneously visualize two sets of input/output connectivity. Similar MCP experimental methods and online publication procedures have also been described previously^20,21,25,90^. The data analyzed in this study were produced through the Brain Initiative Cell Census Network (BICCN: RRID:SCR_015820).

#### Animal subjects

All MCP tract-tracing experiments were performed using 8-week-old male C57BL/6J mice (*n* = 60; Jackson Laboratories) (C57BL/6J IMSR Cat# JAX:000664, RRID:IMSR_JAX:000664). Mice had ad libitum access to food and water and were pair-housed within a temperature- (21–22 °C), humidity- (51%), and light- (12 h: 12 h light/dark cycle) controlled room within the Zilkha Neurogenetic Institute vivarium. All experiments were performed according to the regulatory standards set by the National Institutes of Health Guide for the Care and Use of Laboratory Animals and by the institutional guidelines described by the USC Institutional Animal Care and Use Committee.

#### Tracer injection experiments

The MCP uses a variety of combinations of anterograde and retrograde tracers to simultaneously visualize multiple anatomical pathways within the same Nissl-stained mouse brain. Triple anterograde tracing experiments involved three separate injections of 2.5% *Phaseolus vulgaris* leucoagglutinin (PHAL; Vector Laboratories, Cat# L-1110, RRID:AB_2336656), and AAVs encoding enhanced green fluorescent protein (AAV-GFP; AAV2/1.hSynapsin.EGFP.WPRE.bGH; Penn Vector Core; 7 × 10¹² vg/mL) and tdTomato (AAV1.CAG.tdtomato.WPRE.SV40; Penn Vector Core; 4 × 10¹² vg/mL). An anterograde tracer of 5% biotinylated dextran amine (BDA; Invitrogen) was used in some cases. Retrograde tracers included cholera toxin subunit B conjugates 647, 555, and 488 (CTb; AlexaFluor conjugates, 0.25%; Invitrogen), Fluorogold (FG; 1%; Fluorochrome, LLC), and AAVretro-EF1a-Cre (AAV-retro-Cre; Viral Vector Core; Salk Institute for Biological Studies; 7 × 10¹² vg/mL). To provide further details on specific connectivity patterns, we also performed quadruple retrograde tracer, and rabies/PHAL experiments. Quadruple retrograde tracer experiments involved four different injection sites receiving a unique injection of either 0.25% CTb-647, CTb-555 CTb-488, 1% FG, or AAV-retro-Cre. To trace the connectivity of projection defined neurons, a Cre-dependent anterograde tracing strategy was employed, which involved the delivery of Cre to projection neurons of interest via an injection of AAVretro-EF1a-Cre into their target regions. Next, AAV1.CAG.FLEX.eGFP.WPRE.bGH (RRID:Addgene_51502; 1 × 10¹³ vg/mL) and AAV1.CAG.FLEX.TdTomato.WPRE.bGH (RRID:Addgene_51503) were delivered to the different Cre-expressing SC neuronal populations. Furthermore, to retrieve the morphological information from different tectal projection neurons, Gdel-RV-4tdTomato^58^ (2.14e10 infectious units/mL), and Gdel-RV-4eGFP^59^ (1.51e11 infectious units/mL) tracers of 10 nl volumes were each injected into two different downstream targets. Finally, an AAV-based multi-synaptic tracing method TRIO (**t**racing the **r**elationship of **i**nputs and **o**utputs) was used to reveal mono-synaptic inputs to projection-specific SC cell types. First an AAVretro-EF1a-Cre (7 × 10¹² vg/mL) injection was made in an SC-projection region. At the same time as the AAVretro-EF1a-Cre injection, a Cre-dependent AAV expressing TVA (an avian receptor protein) and rabies glycoprotein (AAV8-hSyn-FLEX-TVA-P2A-mCherry-2A-oG) was injected into the SC (where the projection neuron cell bodies are located). Following three weeks for Cre recombination and transgene expression of TVA and glycoprotein, an EnvA-pseudotyped G-deleted rabies-EGFP (EnvA G-deleted-rabies-GFP; 1.51e11 infectious units/mL) was injected into the same SC injection site. The EnvA/TVA receptor system allows for rabies infection only within the specific projection neuron population^67^. Following infection, the reincorporation of rabies glycoproteins in newly packaged rabies virions within the projection neurons allows the rabies virus to move trans-synaptically to label all neuronal inputs to the specific projection neuron population, including all cortical and brainstem inputs. All cases used in this study are listed in Supplementary Table 2. Though the injections were discrete in volume, some may have spread into adjacent structures. Anterograde injections in the gustatory (GU), visceral (VISC), entorhinal (ENT), perirhinal (PERI), ectorhinal (ECT) areas, and claustrum (CLA) did not produce any evident projections to SC, cases are included in Supplementary Table 2.

#### Statistics and reproducibility

Reproducibility and control of individual variability of labeling was validated in different cases with injections in the same locations. No statistical methods were used to pre-determine sample sizes; our sample sizes are similar to those reported in previous publications^20,21^. In most cases, anterograde tracing results are cross validated by retrograde labeling injections at the anterograde fiber terminal fields and vice versa. Anterograde AAV (or PHAL) tracer injections made into identical locations of a given cortical area in two different mice resulted in identical projection patterns SC. Several other injection cases were repeated as further controls and resulted in identical labeling patterns.

#### Stereotaxic surgeries

On the day of experiment, mice were deeply anesthetized and mounted into a Kopf stereotaxic apparatus where they were maintained under isoflurane gas anesthesia (Datex-Ohmeda vaporizer). For triple anterograde injection experiments, PHAL was iontophoretically delivered via glass micropipettes (inner tip diameter 24–32 μm) using alternating 7 s on/off pulsed positive electrical current (Stoelting Co. current source) for 10 min, and AAVs were delivered via the same method for 2 min (inner tip diameter 8–12 μm). For anterograde/retrograde co-injection experiments, tracer cocktails were iontophoretically delivered via glass micropipettes (inner tip diameter 28–32 μm) using alternating 7 s on/off pulsed positive electrical current (Stoelting Co. current source) for 5 (BDA or AAV/FG) or 10 min (PHAL/CTB-647). For quadruple retrograde tracing experiments, at each injection site, 50 nL of the retrograde tracer was individually pressure-injected via glass micropipettes at a rate of 10 nL/min (Drummond Nanoject III). Rabies injections of 10 nL were individually pressure-injected via glass micropipettes (inner tip diameter 8–12 μm). All injections were placed in the right hemisphere. Injection site coordinates for each surgery case are in Supplementary Table 2 based on the (ML, AP, and DV) coordinate system from the 2008 Allen Brain Reference coronal atlas^22^. Following injections, incisions were sutured, and mice received analgesic pain reliever and were returned to their home cages for recovery.

#### Histology and immunohistochemical processing

After 1–3 weeks post-surgery, each mouse was deeply anesthetized with an overdose of Euthasol and *trans*-cardially perfused with 50 ml of 0.9% saline solution followed by 50 ml of 4% paraformaldehyde (PFA, pH 9.5). Following extraction, brain tissue was post-fixed in 4% PFA for 24–48 h at 4 °C. Fixed brains were embedded in 3% Type I-B agarose (Sigma–Aldrich) and sliced into four series of 50 μm thick coronal sections using a Compresstome (VF-700, Precisionary Instruments, Greenville, NC; RRID:SCR_018409) and stored in cryopreservant at −20 °C. For double co-injection experiments, one series of tissue sections was processed for immunofluorescent tracer localization. For PHAL or AAVretro-EF1a-Cre immunostaining, sections were placed in a blocking solution containing normal donkey serum (Vector Laboratories) and Triton X (VWR) for 1 h. After rinsing in buffer, sections were incubated in PHAL primary antiserum (1:100 rabbit anti-PHAL antibody (Vector Laboratories Cat# AS-2300, RRID:AB_2313686)) or AAVretro-EF1a-Cre primary antiserum (1:100) mixed with donkey serum, Triton X, in KPBS buffer solution for 48–72 h at 4 °C. Sections were then rinsed again in buffer solution and then immersed in secondary antibody solution (donkey serum, Triton X, and 1:500 donkey anti-mouse IgG conjugated with Alexa Fluor 488 (Thermo Fisher Scientific Cat# A-31571, RRID:AB_162542)), or 1:500 donkey anti-rabbit conjugated with CY3 (Jackson ImmunoResearch Labs Cat# 715-165-151, RRID:AB_2315777) for 3 h. BDA immunofluorescence was visualized using a 647- or 568-conjugated Streptavidin. Finally, all sections were stained with Nissl Neurotrace 435/455 (Thermo Fisher Cat# N21479) for 2–3 h to visualize cytoarchitecture. After processing, sections were mounted onto microscope slides and cover slipped using 65% glycerol.

### Imaging and informatics processing

Complete tissue sections were scanned using a ×10 objective lens on an Olympus VS120 slide scanning microscope (RRID:SCR_018411). Each tracer was visualized using appropriately matched fluorescent filters and whole-tissue section images were stitched from tiled scanning into VSI image files. For online publication, raw images are corrected for correct left-right orientation and matched to the nearest Allen Reference Atlas level (ARA)^22^.

#### Connection lens workflow

An informatics workflow was specifically designed to reliably warp, reconstruct, annotate and analyze the labeled pathways in a high-throughput fashion through our in-house image processing software, Connection Lens (Fig. 1b). Tissue sections from each analyzed case were assigned and registered to a standard set of 10 corresponding ARA levels ranging from 84 to 102. All images shown in this manuscript are from the raw data of unwarped, unregistered VSI images. Threshold parameters were individually adjusted for each case and tracer. Adobe Photoshop (RRID:SCR_014199) was used to correct conspicuous artifacts in the threshold output files. Each color channel was brightness/contrast adjusted to maximize labeling visibility (Nissl Neurotrace 435/455 is converted to brightfield), and TIFF images are then converted to JPEG file format.

#### Assessment of injection sites and fibers of passage

All cortical injection cases included in this work are, in our judgment, prototypical representatives of each cortical area. We have demonstrated our targeting accuracy with respect to injection placement, our attention to injection location, and the fidelity of labeling patterns derived from injections to the same cortical location in previous studies^20,21^. In the current report, we also demonstrate our injection placement accuracy and the consistent labeling resulting from injections placed in the same cortical areas as described in detail in Supplementary Methods for Hintiryan et al., 2016. A combination of labeling produced from both anterograde and retrograde cases across cortex and SC was used to generate the input/output connectome diagram (Supplementary Figs. 1 and 5; Fig. 5). An important concern when employing automated analysis of connectivity is the issue of fibers of passage getting annotated as functional connections when in fact no synapses exist. Pathways devoid of synapses can produce bright labeling that can be annotated as positive pixels. This is especially relevant with regard to cortico-tectal fibers that travel from the rostral direction through the SC onto a caudal SC termination site. For example, ARA 86 level exhibited more labeling from fibers of passage entering rostrally from both sensorimotor and higher-order cortical areas such as MOp/MOs (Fig. 2d), PTLp (Fig. 3f), and RSPv-rostral (Supplementary Fig. 4a). Some pixel fibers had evident terminal boutons, as determined by comparison and validation with the raw tissue data, thus these pixels remained for quantification. Pixels that arose from lines of straight bold fibers (indicative of fibers of passage as validated by raw data for each section) void of boutons or synapses were removed from analysis. The pixel values that were indistinguishable from terminals remained as part of the computational analysis.

#### Defining the SC custom atlas and delineating SC zones

The workflow was applied toward analyzing projections from 86 representative cortical injection cases to sections with characteristic labeling across the far rostral SC (ARA 86), rostral SC (ARA 90), middle SC (ARA 96), and caudal SC (ARA 100). ARA level 86 is the furthest rostral where notable fiber terminations were observed. The labeling at level 90 (−3.68 mm from bregma) was the target of rich projections from all cortical areas, representative of all cortico-tectal projections, and exhibited the most segregation of labeling in terms of unique zones. Therefore, it was selected as the representative section for the rostral SC. The SC at level 86 (−3.28 mm from bregma) was the furthest rostral level we could use for analysis that expressed prominent terminals from a subset of cases. Therefore, it was selected as the representative section for the far rostral SC. The SC at levels 96 (−4.28 mm from bregma) and 100 (−4.65 mm from bregma) displayed distinguishable labeling with variable degrees of layer- and zone-specific cortical inputs and far fewer discrete termination fields compared to the rostral SC and were therefore selected to represent the middle and caudal SC, respectively.

In the Allen Brain Reference Atlas (ARA), the SC spans ARA levels 83 through 104, which measures out to 2 mm in the rostral-caudal axis. ARA levels 86 through 100 were selected as they represent the majority of the SC volume across all dimensions. Specifically, these levels span ~1.4 mm in the rostral-caudal axis, and extend up to 3 mm in dorsal-ventral depth and up to 2 mm in midline-lateral width. The extreme rostral levels (83–85) and extreme caudal levels (101–104) span the remaining 0.6 mm in the rostral-caudal axis (about 0.3 mm at each extreme end, respectively), and each extends only about 1 mm in dorsal-ventral depth and 1 mm in midline-lateral width. Based on the dimensions of the SC throughout the reference atlas coordinate frame, the structural volume between atlas levels 86 through 100 constitutes ~90% of the total volume.

We overlapped reconstructed cortico-tectal fibers from the 86 cases to each custom SC level (86, 90, 96, and 100), and determined the boundaries of the four zone delineations based on the average patterns generated. Some cortico-tectal patterns naturally distributed across more than one zone, though the general boundaries were still consistent within the four zones. As representative examples, anterograde AAV injections into the right hemispheres of PTLp (red), VISam (orange), ACAd (green), and MOp (purple) terminate with different laminar and regional patterns in SC (ARA 90) (Fig. 1a). PTLp produced ipsilateral terminals in the intermediate layers of SC, delineating the medial zone (SC.m). VISam produced tiered terminals in the SC superficial and intermediates layers adjacent to SC.m, delineating the centromedial zone (SC.cm). ACAd produced tiered terminals in the SC intermediate and deeper layers, laterally adjacent to the VISam terminal field, delineating the centrolateral zone (SC.cl). MOp produced terminals targeting the far lateral SC, delineating lateral zone (SC.l). Together, these representative cortico-tectal projections reveal layer-specific terminals distributed across four distinct zones along the medial-lateral axis of SC.

#### Polar coordinate analysis and validation of SC zone borders

We designed a computational method to validate our manual annotation and delineation of the zone borders in the SC. We refer to this as the polar coordinate analysis. Given the roughly circular nature of the SC structure relative to the midline, we assigned each pixel of labeling an *x* and *y* coordinate value to plot. This renders the angular position and distribution of terminal labeling for each cortical case. Reconstructed pixel labeling from each case was plotted as a probability distribution graph where the *y-*axis represents the probability density and the *x-*axis represent the theta angle in degrees (θ°) where the labeling is found in the SC. Each case is plotted as a normalized smooth histogram where the area under the curve equals 1. The height of each plot represents how likely the cortical region projects to the SC at any given angle of theta (i.e., the *y-*axis value is the projection density at each angular position). Notably, cases that have a similar histogram also have a similar projection profile which facilitates in assessing the reproducibility of labeling patterns from injection sites at the same ROI.

At each ARA level 86, 90, 96, and 100, the midline θ value was set to 90°, corresponding to beginning of the SC.m zone on the left side of the *x*-axis (Supplementary Fig. 2a). The θ values decreased in value as they shifted to the right corresponding to the SC.cm, SC.cl, and SC.l zone at the far right, respectively. Histogram curves of individual cases with confined projections to each SC zone were plotted on the same graph at each ARA level (Supplementary Fig. 2b). The average distribution plots for each zone at each ARA level were plotted to analyze the approximate θ° range for each zone (Supplementary Fig. 2c). The four average probability distribution plots show peaks with ranges that closely align with the manually delineated borders in the custom atlas levels. Each zone was clearly distinguishable based on the average peaks, with the exception at ARA 100 with SC.cl and SC.l zones where the structure is much smaller relative to the rest of the SC and the peaks are mostly overlapping. Importantly, the geometric radial lines do not entirely reflect the natural curvature of the SC, and our manual borders were delineated based on the raw image data to reflect the natural shape of the structure. Here in Table 1, we provide a list of the approximate theta (θ°) ranges for each SC zone at each atlas level of analysis.

**Table 1:** Angular ranges for each SC zone.

	SC.m	SC.cm	SC.cl	SC.l
ARA 86	90–75°	75–60°	60–45°	45–30°
ARA 90	90–75°	75–55°	55–35°	35–10°
ARA 96	90–75°	75–60°	60–45°	45–5°
ARA 100	90–75°	75–60°	60–45°	45–0°

#### Color-coding, data visualization, and proportional stacked bar charts

The same color scheme within SC zones was used for consistency of reference throughout the diagrams: SC.m (red), SC.cm (orange), SC.cl (green), SC.l (purple). In the topographic map of the brain dorsal view (Figs. 2g, 3b, 5e), cortical areas were color-coded based on their SC zone target. Stacked bar charts for visualization of proportion of labeling across each SC zone (*x*-axis) from each cortical ROI (*y-*axis). Values represent proportion of pixel density for the selected ROI (*n* = 1 per cortical area) distributed across each SC zone across all layers. The chart facilitates an overview representation of how cortico-tectal projections preferentially target-specific zones and how they compare across cortical groups. For complete breakdown of values for zone- and layer-specific proportion of label bar charts, see Supplementary Tables 3 and 4.

#### Community detection and connectivity matrices

We further analyzed the annotation data to objectively identify groups of cortical injection sites that send converging inputs within different SC zones. To perform this final stage of the neuroinformatics pipeline, we first built an adjacency matrix out of our annotation data. The graph structure of the data are relatively simple: nodes and connections are organized as a multi-tree with two levels: the cortex and the four SC zones. We performed community detection (modularity maximization) on ROI annotated data. The overlap annotation per each case within a group was aggregated into a single matrix. Overlap refers to a convergence of terminal fields within the SC between cortical source areas. The overlap value between a source and target ROI is the ratio of common labeling among the two ROIs to total source labeling^21^. Once the aggregated matrix was constructed, we further normalized the data so that the total labelling across each injection site (typically close in the first place) was adjusted to equal with the injection site featuring maximum total labeling. On this normalized matrix we applied the Louvain community detection algorithm at a single scale (gamma 1.0) to the data and identified clusters of injection sites with similar SC termination fields.

As the result of this greedy algorithm being non-deterministic, we performed 100 separate executions, and subsequently calculated a consensus community structure to characterize the 100 executions as a single result. Given the modularity of the data (that is, highly topographic labeling), a modularity optimization algorithm, like the Louvain, was well suited. However, the element of randomness makes it probable that the algorithm will reveal a different community structure over multiple runs. To mitigate this issue, the algorithm was run 1000 times. The community structure that emerged most often, which we defined as the community structure mode (borrowing from statistics) is reported. A mean and standard deviation for the number of communities that was detected across the 1000 runs was also computed. Subsequently, to aid visualization we employed the community structure to reorder and color code an adjacency matrix such that connections were placed close to the diagonal. An accompanying color-coded SC illustrates the spatial arrangement of the communities/zones. The code for generating the illustrative color-coded SC employed a ‘winner takes all’ reconstruction of community terminal fields. For each cell, the method compared overlap data from across injection sites and colored according to the community with the greatest quantity of labeling.

For matrix visualization, we applied an algorithm to modularize ROIs based on community assignment, as well as prioritize connections along the diagonal. Such a visualization provides a high-level overview of the connectivity (Fig. 3g). Community coloring provides more detail by visually encoding segmentation by community, and ultimately, injection site (anterograde visuals). To carry out this process, we developed software to programmatically march through each segmented pixel per each image. Using the ROI name, the algorithm looked up the corresponding community assigned during the consensus community step. Using a table containing a color assigned (by the authors) to each injection site, the algorithm retrieved the injection site associated with the community and colored the pixel with the corresponding injection site color value. A subsequent step took advantage of the fact that each pixel was assigned to only a single community by aggregating all colorized images corresponding to a given atlas level into a single representative image.

### 3D tissue processing and imaging

To assess whether thalamic-projection neurons in the four SC zones were morphologically distinct, representative neurons in each zone were labeled via a G-deleted-rabies injection in the LD (for SC.m/cm), in the RE (for SC.m/cm), and dorsal PF (Supplementary Fig. 7a; Fig. 5f). One week was allowed for tracer transport following injections, after which the animals were perfused. The SHIELD clearing protocol was used for the 3D tissue processing workflow^91,92^, followed by neuronal reconstructions and statistical analyses^81^. Mice were *trans*-cardially perfused with cold saline and SHIELD perfusion solution. The brains were extracted and incubated in the SHIELD perfusion solution at 4 °C for 48 h. The SHIELD perfusion solution was replaced with the SHIELD OFF solution and tissues were incubated at 4 °C for 24 h. The SHIELD OFF solution was replaced with the SHIELD ON solution and the tissues were incubated at 37 °C for 24 h. The whole brain was cut into 400 µm sections and were cleared in the SDS buffer at 37 °C for 72 h. The sections were then washed three times with KPBS and incubated in KPBS at 4 °C for 24 h. Sections were mounted and cover slipped onto 25x75x1mm glass slides with an index matching solution 100% (EasyIndex, LifeCanvas Technologies, #EI-Z1001). Sections were imaged with a high speed spinning disk confocal microscope (Andor Dragonfly 202 Imaging System, Andor an Oxford Instruments Company, CR-DFLY-202-2540). ×10 magnification (NA 0.40, Olympus, UPLXAPO10X) was used to acquire an overview after which ×30 magnification (NA 1.05, Olympus, UPLSAPO30xSIR) was used to image through the SC ipsilateral to the injection site at 1 µm z steps. Sections were imaged with four excitation wavelengths (nm): 405 (blue Nissl background), 488 (green for Rabies), 561 (red for Rabies) with respective emission detection wavelengths of 450, 525, and 600.

#### 3D reconstructions, visualizations, and analysis of neuronal morphology

Manual reconstruction of the neurons was performed using Aivia (version.8.8.1, DRVision) (Fig. 5f), and geometric processing of neuron models, including smoothing, was performed using the Quantitative Imaging Toolkit (QIT) NeuronTransform and NeuronFilter modules^93^ (also available at http://cabeen.io/qitwiki). neuTube was used to facilitate visualizations of reconstructions^94^. All reconstructions are being made freely available in the Dong archive of www.NeuroMorpho.Org^63^ (RRID:SCR_002145). Although we wanted to compare projection neurons from each SC zone to each thalamic area, neurons from each injection were spread across two to four zones. This allowed us to compare SC neurons in neighboring zones that project to the same thalamic nucleus, as well as those that project to a different thalamic nucleus. Three groups were assigned from the RE rabies injected case based on the location of retrogradely-labeled cells in SC: RE-SC.m (*n* = 23), RE-SC.cm (*n* = 27), and RE-SC.cl (*n* = 4). Two groups were assigned from the LD case: LD-SC.m (*n* = 4), and LD-SC.cm (*n* = 4). Four groups were assigned from the PF/MPT case: PF/MPT-SC.m (*n* = 10), PF/MPT-SC.cm (*n* = 6), PF/MPT-SC.cl (*n* = 9), PF/MPT-SC.l (*n* = 5). Cells for this analysis were selected as representative thalamus-projecting SC neurons and are not representative of the entire population of neurons within the four zones or layers.

#### Statistical analyses on groups of reconstructions

To obtain an overall view of the dendritic morphology of SC-projection neurons located in the SC.m, SC.cm, and SC.cl, we applied the classic Sholl analysis using Fiji (ImageJ, RRID:SCR_003070). Quantitative morphological parameters characterizing arbor morphology were obtained from L-Measure^57^, and statistical analyses were performed using the R computing environment (RStudio Version 1.1.463). Using all of the measured morphological parameters, principal component analysis (PCA) was run to reduce the dimensionality and create a 2D scatterplot. The PCA shows the segregation of SC zone-specific neurons based on the measured features, namely the segregation of SC.m-PF/MPT from all groups, including SC.m projecting neurons to both RE and LD. Neurons from SC.cm/cl/l projecting to PF/MPT are completely segregated from all SC.m projecting groups (Supplementary Fig. 7b). We calculated a comprehensive battery of standard measurements, including the number of bifurcations, contraction, width, and branch path length, to compare morphological features. Statistically significant differences in several morphological features were detected across all pairwise comparisons (Supplementary Table 5). Examples of parameters with significant group differences and greater loading values influencing the PCA are presented with whisker plots (Supplementary Fig. 7c). Two-sided pairwise Wilcoxon rank sum tests were performed, and the parameters that survived FDR correction for multiple testing with a *p*-value < 0.05 are reported. Significant differences were detected across several pairwise parameters. Our experiments used code provided by the authors online diagrams using NeuronTools (https://github.com/Nevermore520/NeuronTools, RRID:SCR_017450) and the Wasserstein metric (https://bitbucket.org/grey_narn/geom_matching/src, SRC SCR_018424). The results were visualized in matrix plots created using R (Supplementary Fig. 7b, c).

### Reporting summary

Further information on research design is available in the Nature Research Reporting Summary linked to this article.

## Supplementary information

Supplementary Information

Reporting Summary

## Data Availability

All data used in this study are available from the corresponding author upon reasonable request. Anatomical tracer image data are available through our iConnectome viewer as part of the Mouse Connectome Project at USC (http://www.mouseconnectome.org). All reconstructions are being made freely available in the Dong archive of www.NeuroMorpho.Org. We used various software to navigate images and data including, FIJI/ImageJ (v1.53a), neuTube (v1.0z), Adobe Photoshop (v21.2.2), Aivia (v8.8.1, DRVision), and RStudio (v1.1.463).

## Code availability

All custom code that we developed for data analysis is available at https://github.com/lhgarciaa/sc_project and https://github.com/lhgarciaa/sc_analysis. Code used for other data analyses are freely accessible. For the Louvain algorithm implementation, the Brain Connectivity Toolbox (BCT) was employed, available at https://sites.google.com/site/bctnet/. For matrix visualization, the grid_communities algorithm for matrix visualizations are available at https://github.com/aestrivex/bctpy, and https://sites.google.com/site/bctnet/. Geometric processing of neuron models was performed using the Quantitative Imaging Toolkit (QIT), available here http://cabeen.io/qitwiki. We also computed persistence diagrams using NeuronTools (https://github.com/Nevermore520/NeuronTools; RID:SCR_017450) and then computed inter-neuron distances using the Wasserstein metric (https://bitbucket.org/grey_narn/geom_matching/src). In addition, we have used several open-source Python packages: scipy (v0.17.0), matplotlib (v1.5.1), ipython (v2.4.1), ipython-genutils (v0.2.0), jupyter (v1.0.0), jupyter-client (v5.2.3), jupyter-console (v5.2.0), jupyter-core (v4.4.0), pandas (v0.23.3), sympy (v1.2), nose (v1.3.7), pydot (v1.2.3), bctpy (v0.5.0), pyflakes (v1.1.0), pandas (v0.23.3), cycler (v0.9.0), plotly (v3.0.0).
