# Supplementary material for: Organization of the inputs and outputs of the mouse superior colliculus
Source: Nat Commun. 2021 Jun 28;12:4004. doi: 10.1038/s41467-021-24241-2 (PMC8239028; doi:10.1038/s41467-021-24241-2)
Supplement: Supplementary file 1 — Supplementary Information [file 41467_2021_24241_MOESM1_ESM.pdf]

**SUPPLEMENTARY INFORMATION**

# Organization of the inputs and outputs of the mouse superior colliculus

---

Nora L. Benavidez[1-3], Michael S. Bienkowski[2], Muye Zhu[2,3], Luis H. Garcia[2,3], Marina Fayzullina[2,3], Lei Gao[2,3], Ian Bowman[2,3], Lin Gou[2,3], Neda Khanjani[2], Kaelan R. Cotter[2,3], Laura Korobkova[1,2], Marlene Becerra[2], Chunru Cao[2,3], Monica Y. Song[1-3], Bin Zhang[2,3], Seita Yamashita[2,3], Amanda J. Tugangui[2,3], Brian Zingg[2,3], Kasey Rose[1], Darrick Lo[2,3], Nicholas N. Foster[2,3], Sarvia Aquino[2], Tyler Boesen[2,3], Hyun-Seung Mun[2,3], Ian R. Wickersham[4], Giorgio A. Ascoli[5], Houri Hintiryan[2,3] & Hong-Wei Dong[2,3]*

[1] *Neuroscience Graduate Program, University of Southern California, Los Angeles, CA 90033, USA.*

[2] *Stevens Neuroimaging and Informatics Institute, Laboratory of Neuro Imaging, Keck School of Medicine, University of Southern California, Los Angeles, CA 90033, USA.*

[3] *Current affiliation: UCLA Brain Research & Artificial Intelligence Nexus, Department of Neurobiology, David Geffen School of Medicine, University of California Los Angeles, Los Angeles, CA 90095, USA.*

[4] *McGovern Institute for Brain Research, Massachusetts Institute of Technology, Cambridge, MA 02139, USA.*

[5] *Krasnow Institute for Advanced Study, George Mason University, Fairfax, VA 22030, USA.*

*Corresponding author: Hong-Wei Dong, M.D., Ph.D. (hongweid@mednet.ucla.edu)

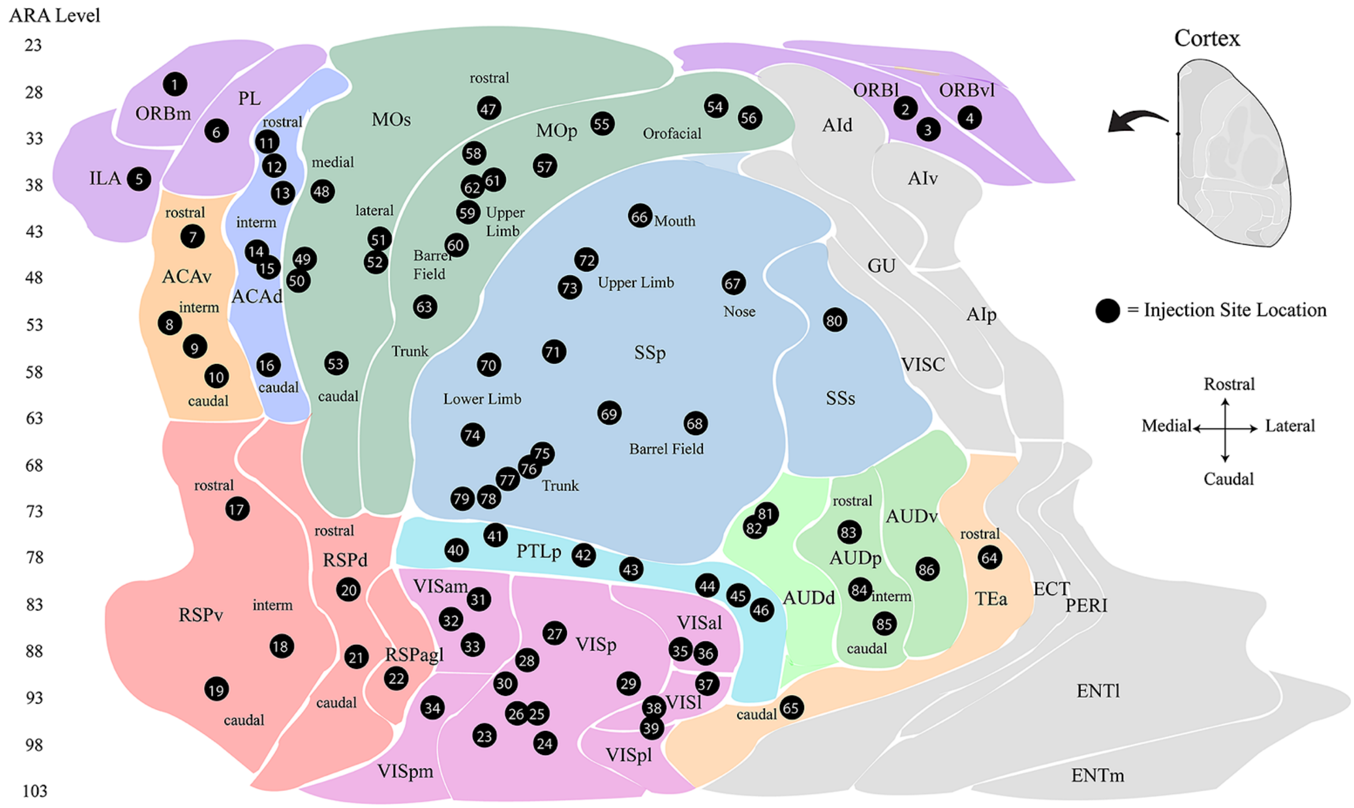


1. ORBm (28) SW110808-04A
2. ORBl (35) SW180117-01A
3. ORBl (37) SW110808-02A
4. ORBvl (33) SW190327-02A
5. ILA (37) SW190315-07A
6. PL (33) SW190315-05A

7. ACAv rostral (44) SW120403-02A
8. ACAv interm (54) SW121031-04B
9. ACAv interm (56) SW110321-04B
10. ACAv caudal (60) SW180228-03A

11. ACAd rostral (36) SW151210-01A
12. ACAd rostral (37) SW151211-01A
13. ACAd interm (42) SW180227-01A
14. ACAd interm (46) SW121031-03B
15. ACAd interm (47) SW180227-02A
16. ACAd caudal (58) SW151211-02A

17. RSPv rostral (73) SW110615-03
18. RSPv interm (88) SW190327-04A
19. RSPv caudal (90) SW151211-01A
20. RSPd rostral (83) SW110613-03B
21. RSPd caudal (88) SW110614-01B
22. RSPagl (91) SW110615-01B

23. VISp caudal (95) SW121221-03A
24. VISp caudolat (96) SW121221-02A
25. VISp caudolat (94) SW180302-05A
26. VISp caudomed (96) SW121221-02A
27. VISp rostromed (87) SW180713-04A
28. VISp rostral (86) SW121221-03A
29. VISp (92) SW151211-01A
30. VISp (90) SW180713-03A
31. VISam (83) SW121031-03B
32. VISam (84) SW151210-02A
33. VISam (86) SW151211-02A
34. VISpm (94) SW190327-04A
35. VISal (90) SW180302-05A
36. VISal (91) SW180302-04A
37. VISl (92) SW180815-01A
38. VISl (92) SW120819-04A
39. VISl/pl (97) SW180522-04A

40. PTLp med (76) SW180717-03A
41. PTLp med (76) SW180717-03A
42. PTLp med (77) SW180424-01A
43. PTLp med (77) SW180424-01A
44. PTLp lat (81) SW180302-05A
45. PTLp lat (82) SW151215-03A
46. PTLp lat (82) SW180302-04A

47. MOs rostral (31) SW190315-05A
48. MOs medial (40) SW180227-01A
49. MOs medial (47) SW180227-02A
50. MOs medial (49) SW121210-02A
51. MOs lateral (44) SW171130-02A
52. MOs lateral (46) SW171130-02A
53. MOs caudal (58) SW110322-03A
54. MOp oro (31) SW190315-05A
55. MOp oro (34) SW171101-01A
56. MOp oro (32) SW180516-02A
57. MOp oro (40) SW171130-02A
58. MOp oro (35) SW180117-01A
59. MOp ul (41) SW180117-01A
60. MOp ul (44) SW171101-01A
61. MOp bfd (38) SW171101-01A
62. MOp bfd (40) SW180516-02A
63. MOp tr (52) SW180516-02A

64. TEa rostral (78) SW120125-01A
65. TEa caudal (94) SW120819-04A

66. SSp-m (43) SW170410-04A
67. SSp-n (50) SW110516-02B
68. SSp-bfd (64) l SW181109-09A
69. SSp-bfd (64) m SW181109-09A
70. SSp-ll (56) SW170410-03A
71. SSp-ul (58) SW110322-03A
72. SSp-ul (47) SW170410-03A
73. SSp-ul (49) SW170410-04A
74. SSp-tr/ll (70) SW121031-03B
75. SSp-tr (68) SW180713-04A
76. SSp-tr (68) SW180713-03A
77. SSp-tr (74) SW121031-04B
78. SSp-tr (74) SW151210-02A
79. SSp-tr (76) SW151211-02A
80. SSs (53) SW151210-01A

81. AUDd (74) SW180713-03A
82. AUDd (74) SW180713-04A
83. AUDp rostral (76) SW190315-09A
84. AUDp interm (84) SW121031-04B
85. AUDp caudal (86) SW190315-09A
86. AUDv (70) SW190315-09A

**Supplementary Figure 1 | Flat map of mouse cortex with injection sites names.**
Shaded brain region corresponds to same shaded color box with corresponding numbers for each case. Generated by expanding the length of each cortical area from the coordinate framework on the Allen Institute Adult Mouse Brain Atlas. Filled circles indicate location of injection site. List of injection sites, coordinates, and location can be cross-referenced by index number with Supplementary Table 2.

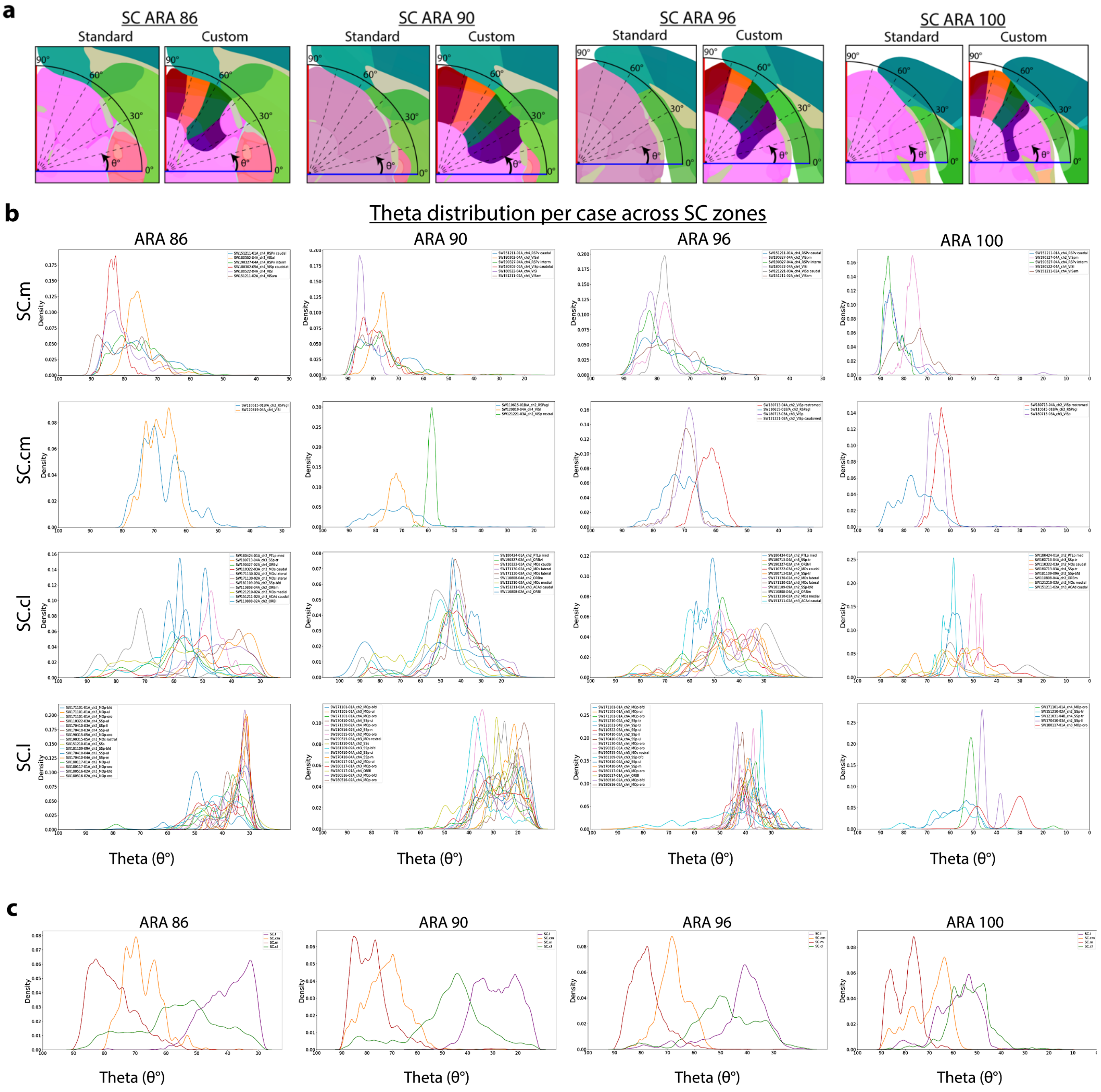
a
SC ARA 86
SC ARA 90
SC ARA 96
SC ARA 100
Standard
Custom
90°
60°
30°
0°
θ°
b
Theta distribution per case across SC zones
ARA 86
ARA 90
ARA 96
ARA 100
SC.m
SC.cm
SC.cl
SC.l
Density
Theta (θ°)
c
ARA 86
ARA 90
ARA 96
ARA 100
Density
Theta (θ°)

**Supplementary Figure 2 | Probability distribution plots.**
**a)** Atlas levels for the SC from rostral to caudal: ARA levels 86, 90, 96, and 100. Left side is polar coordinate ranges used to quantify angular distribution of thresholded pixel labeling in SC. Angles represented by theta (θ°) values where midline starts at 90° and ranges toward 0° at lateral angles. Right side is the custom SC atlas with overlay of angular range showing coarse alignment of manually delineated borders in SC. Color-code associations: red (SC.m), orange (SC.cm), green (SC.cl), purple (SC.l). **b)** Probability distribution plots organized by individual cortical cases that target distinct zones. SC.m, SC.cm, SC.cl, and SC.l. *X*-axis represents pixel density, *y*-axis represents theta angle from medial to lateral. Columns of panels are organized by ARA level displaying plots of individual cases that preferentially target to each SC zone. Top row plots from cases that target SC.m (n=7), next row SC.cm (n=4), next row SC.cl (n=12), and last row SC.l (n=20). **c)** Average of probability distribution plots from multiple cases. Average is based on cases from data in b.

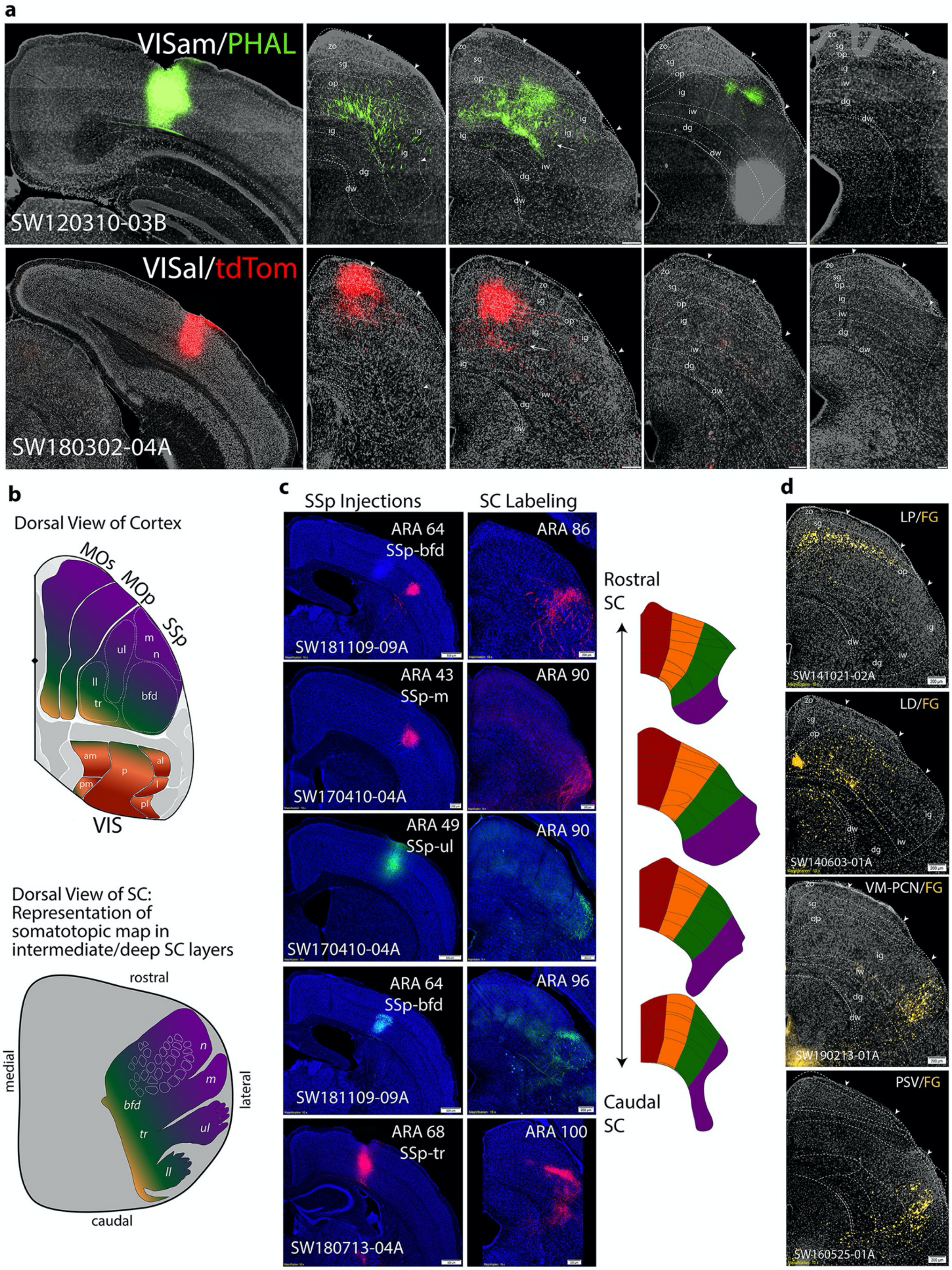
a
VISam/PHAL
SW120310-03B
VISal/tdTom
SW180302-04A
zo
sg
op
ig
iw
dg
dw
b
Dorsal View of Cortex
MOs
MOp
SSp
m
n
ul
ll
tr
bfd
am
p
al
pm
l
pl
VIS
Dorsal View of SC:
Representation of
somatotopic map in
intermediate/deep SC layers
rostral
medial
lateral
caudal
n
m
bfd
ul
tr
ll
c
SSp Injections
SC Labeling
ARA 64
SSp-bfd
SW181109-09A
ARA 86
ARA 43
SSp-m
SW170410-04A
ARA 90
ARA 49
SSp-ul
SW170410-04A
ARA 90
ARA 64
SSp-bfd
SW181109-09A
ARA 96
ARA 68
SSp-tr
SW180713-04A
ARA 100
Rostral
SC
Caudal
SC
d
LP/FG
SW141021-02A
LD/FG
SW140603-01A
VM-PCN/FG
SW190213-01A
PSV/FG
SW160525-01A

**Supplementary Figure 3 | Sensory related connectivity with SC.**

**a)** Raw data of higher order visual areas VISam and VISal. A PHAL injection into VISam (SW120310-03B) projects mainly to intermediate layers in SC.cm, with some terminals in superficial layers. An AAV-tdTomato injection into VISal (SW18080302-04A) projects densely to superficial layers of SC.m with some fibers in the intermediate layers. **b)** Within the cortex, somatic sensorimotor areas are each organized into distinct highly interconnected subnetworks that integrate the somatotopic body map and project subcortically to the SC. Body parts include mouth (m), nose (n), upper limb (ul), lower limb (ll), barrel-field (bfd), and trunk (tr). The dorsal view of the cortical map is color-coded with the dorsal view of the SC body part region it projects to (consistent with the color-coded zones in the SC custom atlas). This organization is conserved topographically as illustrated by dorsal view of SC with overlayed homunculus of somatosensory body representation. **c)** Injection sites into distinct SSp regions project to adjacent and distinct SC.l or SC.cl zones across rostral to caudal levels show adjacent body part projections. Color-code associations: red (SC.m), orange (SC.cm), green (SC.cl), purple (SC.l). Scale bars are 200 µm for all SC panels; 500 µm for injection site panels. **d)** Retrograde labeling of fluorogold (FG)-labeled cells in the SC from four separate injections. First panel: injection into the lateroposterior nucleus of thalamus (LP) back-labeled cells across superficial layers in SC.m and SC.cm zones. Second panel: injection into the laterodorsal nucleus of thalamus (LD) back-labeled cells across superficial and intermediate layers predominantly in SC.cm and SC.m, with sparse cells in SC.cl. Third panel: injection into the ventromedial nucleus (VM) and paracentral nucleus of thalamus (PCN) back-labeled cells in the intermediate and deep layers confined to the SC.l zone.

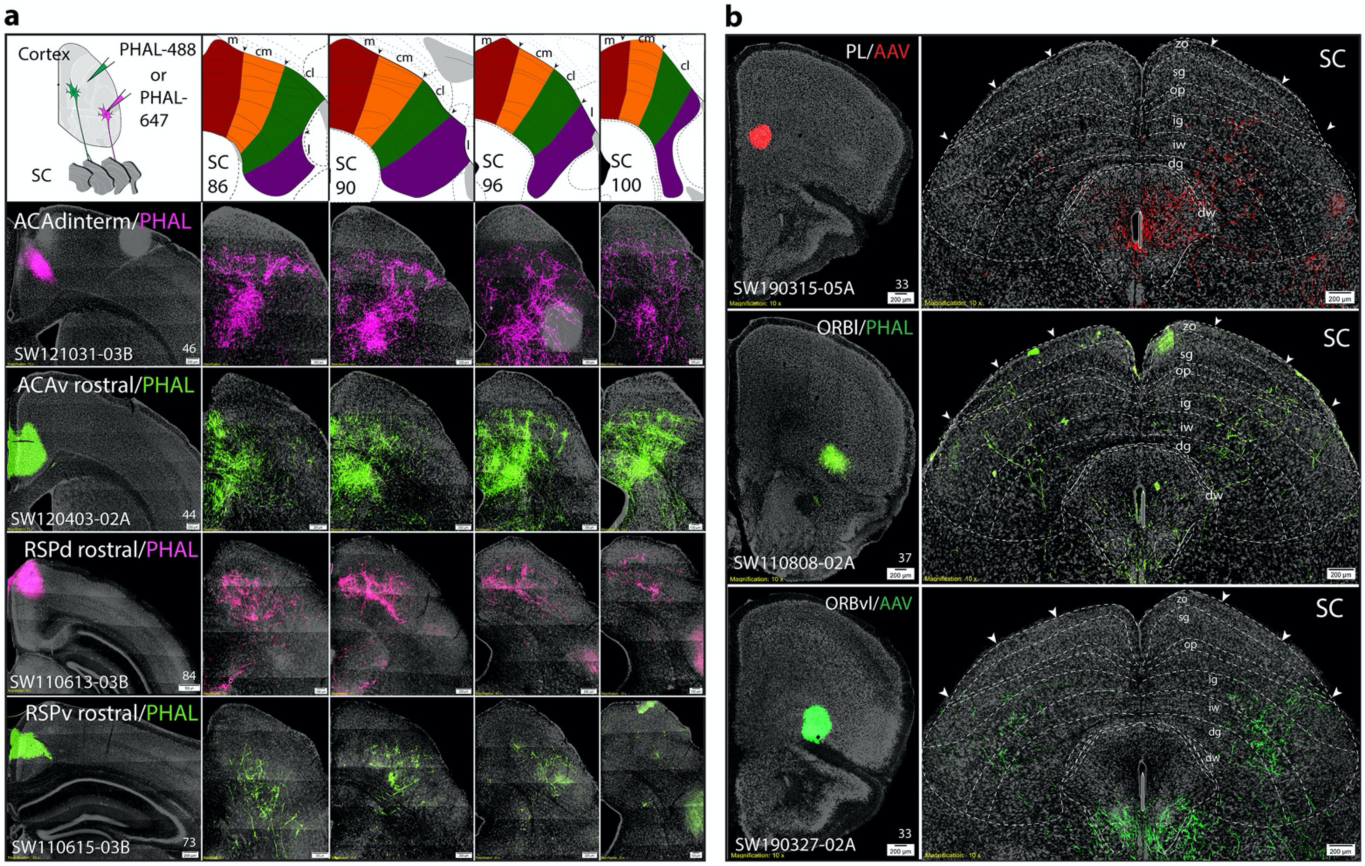


**Supplementary Figure 4 | Higher-order cortico-tectal arrays.**

**a)** Data array of individual cases from higher order projections to SC. Injection sites from top to bottom: ACAd-intermediate, ACAv-rostral, RSPd-rostral, RSPv-rostral. **b)** Three cases with single anterograde injections into PL (AAV), ORBl (PHAL), and ORBvl with their respective bilateral projections predominately to SC.cl and SC.l zones at ARA 90. Scale bars are 200 µm.

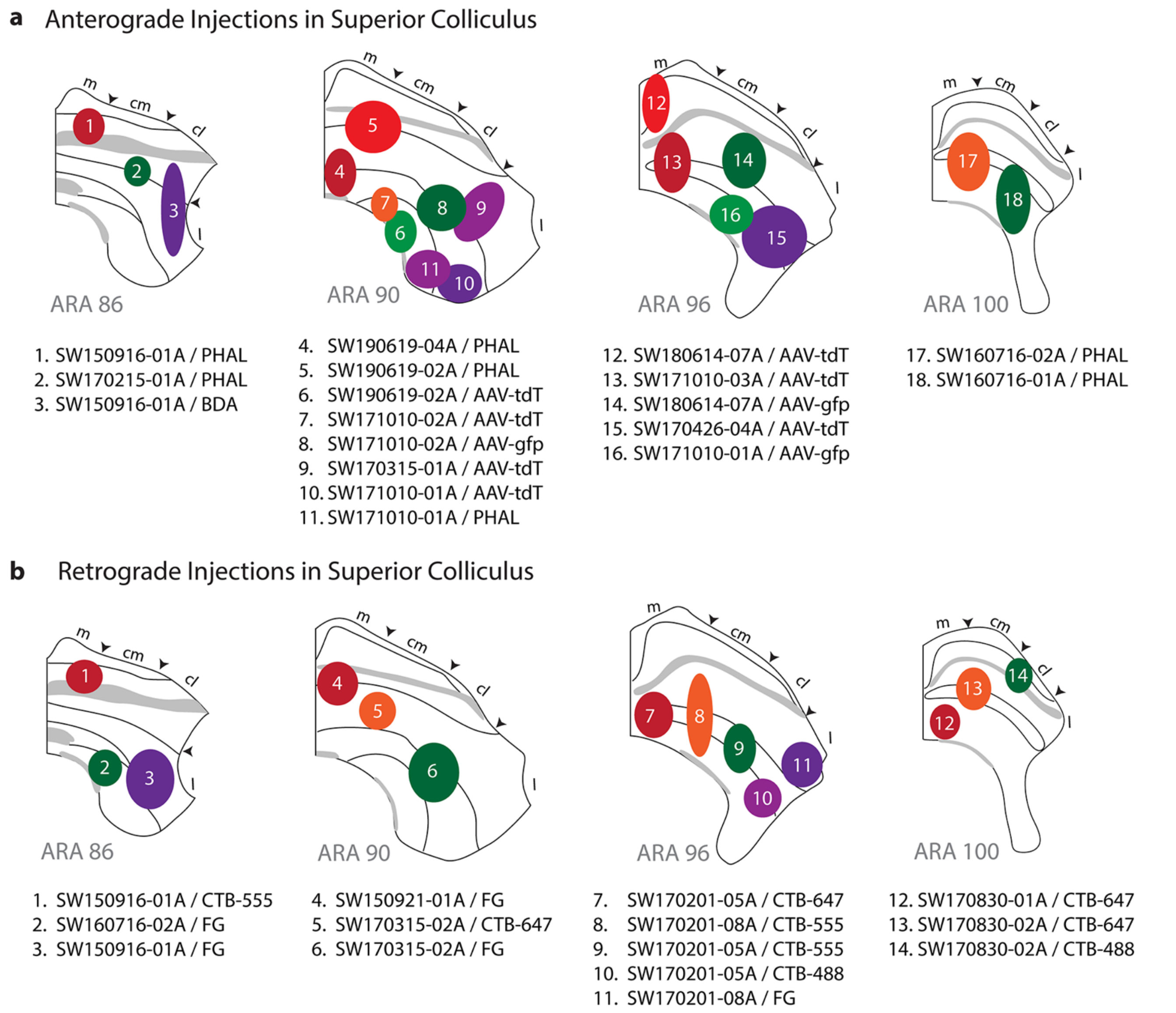


**Supplementary Figure 5 | Injection sites in SC.**

**a)** Anterograde injection sites in superior colliculus. **b)** Retrograde injection sites. The list can be cross-referenced with Supplementary Table 2 for complete details of injection sites, coordinates, and locations. Color-code associations: red (SC.m), orange (SC.cm), green (SC.cl), purple (SC.l). *Abbreviations: AAV, adeno-associated virus; AAV-tdT, AAV-tdTomato; AAV-gfp, AAV-green fluorescent protein; ARA, Allen Reference Atlas; BDA, biotinylated dextran amine; CTB, cholera toxin subunit B conjugates (488, 555, 567); FG, Fluorogold; PHAL, phaseolus vulgaris leucoagglutinin; SC, superior colliculus; m, medial zone; cm, centromedial zone; cl, centrolateral zone; l, lateral zone.*

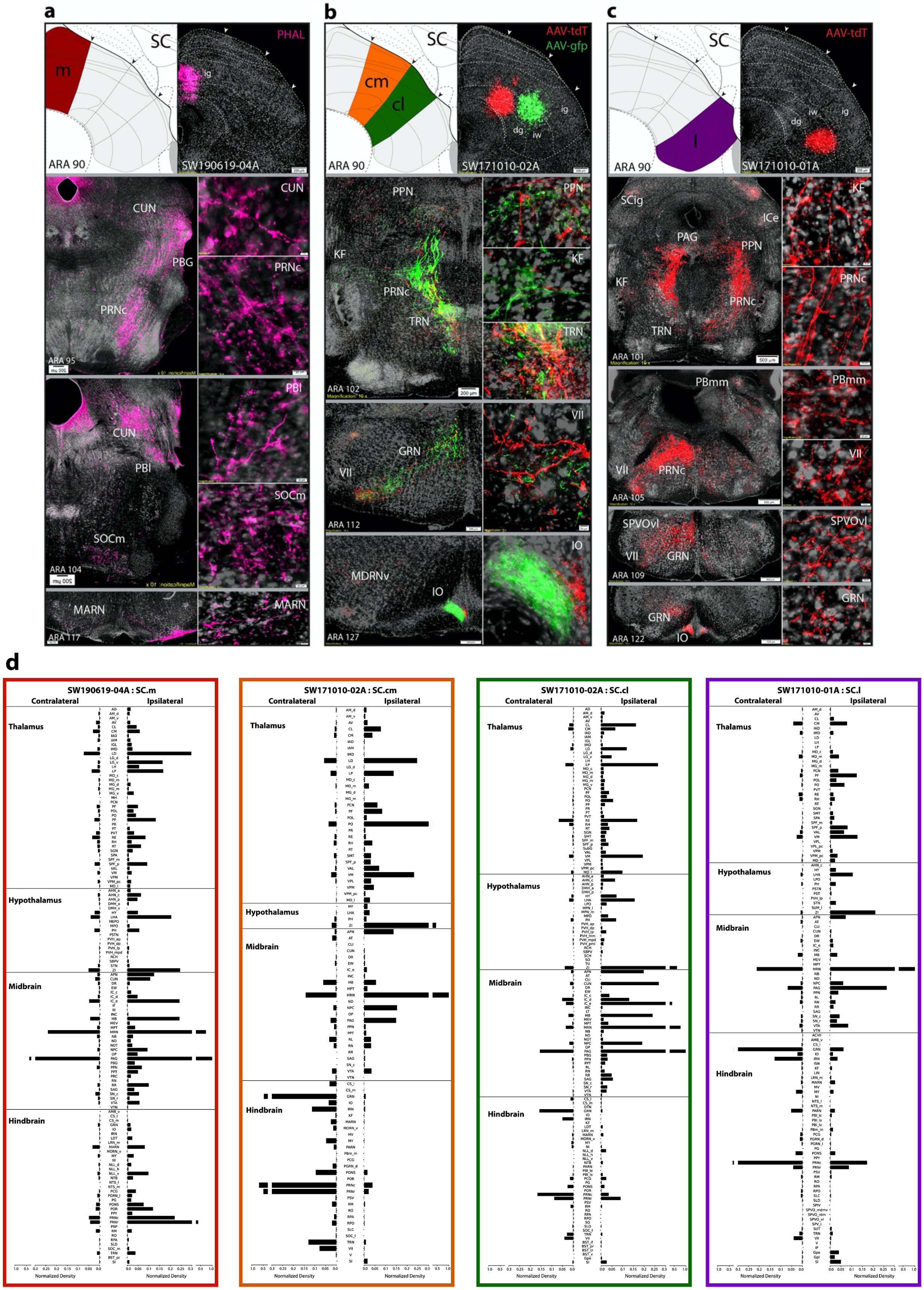
a
SC
m
PHAL
ig
ARA 90
SW190619-04A
CUN
PBG
PRNc
ARA 95
PBI
SOCm
ARA 104
MARN
ARA 117
b
SC
cm
cl
AAV-tdT
AAV-gfp
ig
dg
iw
ARA 90
SW171010-02A
PPN
KF
PRNc
TRN
ARA 102
GRN
VII
ARA 112
MDRNv
IO
ARA 127
c
SC
l
AAV-tdT
iw
ig
dg
ARA 90
SW171010-01A
SCig
ICe
PAG
PPN
KF
PRNc
TRN
ARA 101
PBmm
VII
ARA 105
SPVOvl
GRN
ARA 109
IO
ARA 122
d
SW190619-04A : SC.m
SW171010-02A : SC.cm
SW171010-02A : SC.cl
SW171010-01A : SC.l
Contralateral
Ipsilateral
Thalamus
Hypothalamus
Midbrain
Hindbrain
Normalized Density

**Supplementary Figure 6 | Downstream projections from distinct SC zones to brainstem regions.**
**a)** PHAL injection into SC.m-ig show prominent ipsilateral projections to midbrain and hindbrain regions. **b)** AAV-tdTomato injection into SC.cm-ig/iw and AAV-gfp injection into SC.cl-ig/iw produce downstream targets similar brainstem regions, with segregated but adjacent outputs in IO. **c)** Downstream projections from AAV-tdTomato injection in SC.l in intermediate white and deep grey layers. See Supplementary Table 1 for complete list of abbreviations. **d)** Quantification of four anterograde injections into each SC zone. Plots represent reconstructed pixel data from output projection terminals of SC throughout the brain. Distributions to contralateral and ipsilateral brain regions are shown, with brain regions organized based on ontological hierarchy in the Allen Reference Atlas ( *y*-axis). Data normalization assigns values based on minimum threshold pixel value (0.0006) and maximum threshold pixel value (1) which represents the brain region that receives the highest value of pixels (*x*-axis).

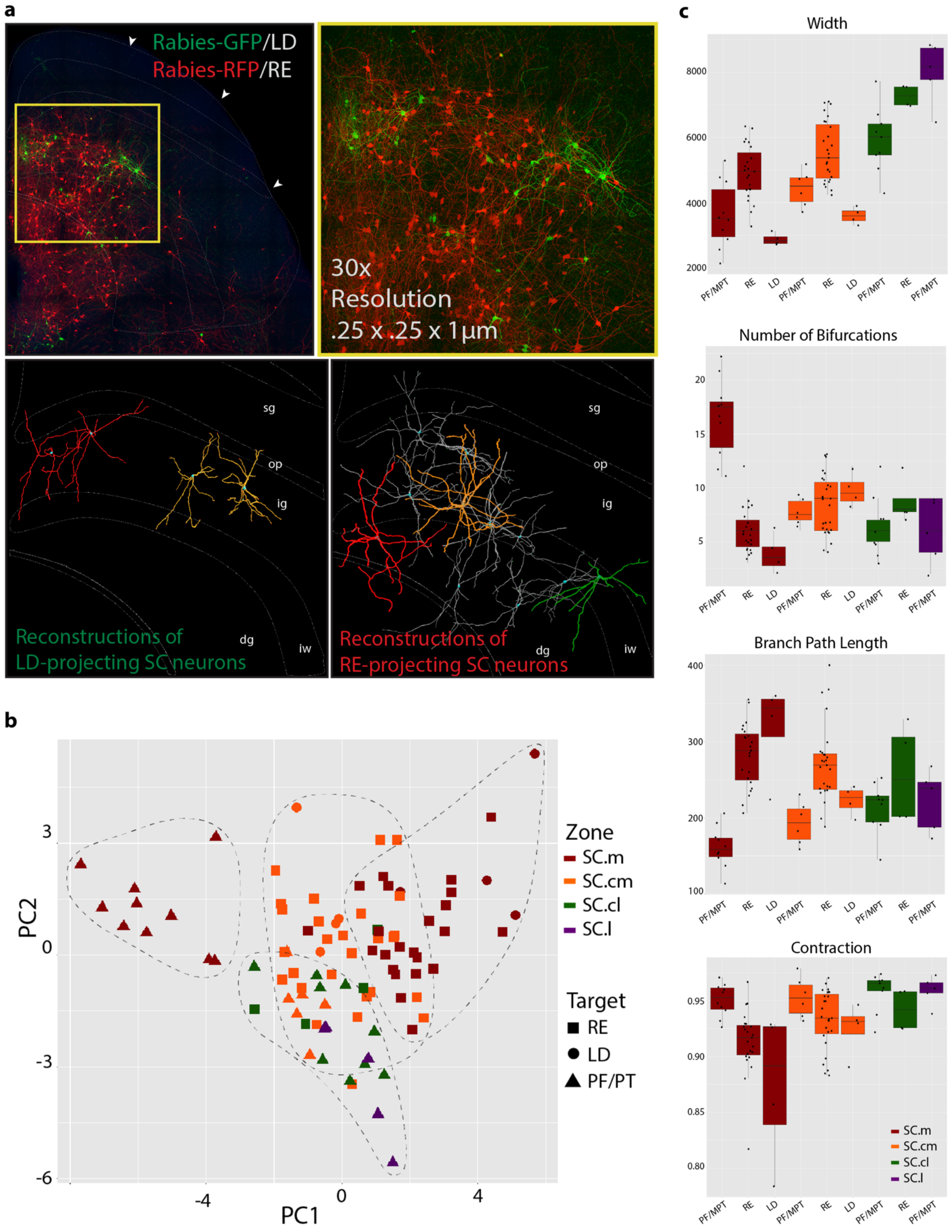
a
Rabies-GFP/LD
Rabies-RFP/RE
30x
Resolution
.25 x .25 x 1µm
sg
op
ig
dg
iw
Reconstructions of
LD-projecting SC neurons
Reconstructions of
RE-projecting SC neurons
b
PC2
PC1
Zone
SC.m
SC.cm
SC.cl
SC.l
Target
RE
LD
PF/PT
c
Width
Number of Bifurcations
Branch Path Length
Contraction
PF/MPT
RE
LD

**Supplementary Figure 7 | Characterization of neuronal cell types in the SC based on their anatomical locations, projection targets and neuronal morphology.**

**a)** LD projecting neurons in SC labeled with rabies-GFP in SC.m/cm-ig, and RE projecting neurons labeled with rabies-RFP injected into cells in SC.m/cm-ig/iw/dg. Tissue was processed by the SHIELD clearing protocol and followed by confocal imaging for 3D-reconstruction. Dendrites from LD and RE-projecting SC neurons were reconstructed to visualize differences and dendritic arborizations across SC layers and zones. The LD-projecting SC.m/SC.cm neurons had pyramidal-shaped cell bodies with dendrites that extended dorsally to the superficial optic layers and within the intermediate layers. Of the RE-projecting SC.m/cm neurons, some had dendrites that extended into superficial and intermediate layers, with others that extended into intermediate and deep layers. A subset of neurons extends dendrites horizontally, suggesting they integrate information within the same layer across zones. Other neurons extend vertically, suggesting these receive multimodal information integrated within multiple SC layers. **b)** Principal component analysis (PCA) shows segregation of zone- and target-specific cells based on measured morphological features (see Methods). Number of SC projection neuron reconstructions to RE (n=54), LD (n=8), and PF/MPT (n=31). **c)** Data are presented as whisker plots, where the center line represents the median, box limits show the upper and lower quartiles, and the whiskers represent the minimum and maximum data values. Examples of reports from pairwise tests of morphological parameters that survived the false discovery rate correction tests. Width measures the overall arbor size of neurons. RE-projecting cells in SC.cl and PF/MPT-projecting cells in SC.l are more than twice the width of LD-projecting cells in SC.m. Number of bifurcations compartmentalizes the arborizations into functionally distinct synaptic integration elements. Branch length represents a ratio between the width and number of bifurcations measures, and provides an indication of how they co-vary. It also relates to the number of synapses that can be received on a single computational element. Contraction measures the efficient occupancy of space to reach as many axonal boutons as possible in nearby and further distances. *P*-values for all parameters can be found in Supplementary Table 6. *Abbreviations: LD, laterodorsal nucleus; PF/MPT, parafascicular nucleus of thalamus/midbrain pretectal area; RE, reuniens nucleus of thalamus; SC, superior colliculus; SC layers: zo, zonal; sg, superficial grey; op, optic; ig, intermediate grey; iw, intermediate white; dg, deep grey; dw, deep white.*

**Supplementary Table 1 | Brain Region Abbreviation List.**

**ACAd**, Anterior cingulate area cortex, dorsal part
**ACAv**, Anterior cingulate area cortex, ventral part
**AD**, Anterodorsal nucleus thalamus
**AId**, Agranular insular area, dorsal part
**AIv**, Agranular insular area, ventral part
**AIp**, Agranular insular area, posterior part
**APN**, Anterior pretectal nucleus
**AUDd**, Auditory cortex, dorsal part
**AUDp**, Primary auditory cortex
**AUDv**, Auditory cortex, ventral part
**AV**, Anteroventral nucleus thalamus
**BLA**, Basolateral amygdalar nucleus
  **BLAa**, Basolateral amygdalar nucleus, anterior part
  **BLAp**, Basolateral amygdalar nucleus, posterior part
**BMA**, Basomedial amygdalar nucleus
  **BMAa**, Basomedial amygdalar nucleus, anterior part
  **BMAp**, Basomedial amygdalar nucleus, posterior part
**CA1**, Field CA1, hippocampal formation
**CA3**, Field CA3, hippocampal formation
**CLA**, Claustrum
**CM**, Centromedial nucleus thalamus
**CP**, Caudate putamen
    **CPc.d**, Caudate putamen, caudal, dorsal domain
    **CPi.dl**, Caudate putamen, intermediate, dorsolateral domain
    **CPi.vl**, Caudate putamen, intermediate, ventrolateral domain
    **CPi.vm**, Caudate putamen, intermediate, ventromedial domain
    **CPr**, Caudate putamen, rostral
**CUN**, Cuneate nucleus
**DN,** Dentate nucleus
**DG**, Dentate gyrus
**DTN**, Dorsal tegmental nucleus
**ENT**, Entorhinal cortex
**GRN**, Gigantocellular reticular nucleus,
**GU**, Gustatory cortex
**Hb,** Habenula nucleus thalamus
**IAM**, Interanteromedial nucleus thalamus
**ICe**, Inferior colliculus, external part
**ILA**, Infralimbic area cortex
**IO**, Inferior olivary nucleus
**IRN**, Intermediate reticular nucleus
**KF**, Koelliker-Fuse subnucleus
**LA**, Lateral amygdalar area
**LD**, Laterodorsal nucleus thalamus
**LG**, Lateral geniculate nucleus thalamus
**LP,** Lateroposterior nucleus thalamus

**LM**, Lateral mammillary nucleus
**MARN**, Magnocellular reticular nucleus
**MD,** Mediodorsal nucleus thalamus
**MDRNv**, Medullary reticular nucleus, ventral part
**MOp,** Primary motor cortex
  **MOp-oro,** Primary motor cortex, orofacial part
**MOs,** Secondary motor cortex
**MOs-fef**, Secondary motor cortex, frontal eye field
**MRN,** Midbrain reticular nucleus
**MPT**, Midbrain pretectal region
**ORBl**, Orbitofrontal area cortex, lateral part
**ORBm**, Orbitofrontal area cortex, medial part
**ORBvl**, Orbitofrontal area cortex, ventrolateral part
**PAG**, Periaquaductal gray nucleus
  **PAG.dl**, Periaquaductal gray nucleus, dorsolateral part
  **PAG.vl**, Periaquaductal gray nucleus, ventrolateral part
**PAR**, Parasubiculum
**PARN**, Parvicellular reticular nucleus
**PBG,** Parabigeminal nucleus
**PBl**, Parabrachial nucleus, lateral part
**PBmm**, Parabrachial nucleus, mediomedial part
**PCN**, Pericentral nucleus thalamus
**PERI**, Perirhinal area cortex
**PF,** Parafascicular nucleus
  **PF.ll,** Parafascicular nucleus, lower limb
  **PF.m,** Parafascicular nucleus, mouth domain
  **PF.tr,** Parafascicular nucleus, trunk domain
  **PF.ul,** Parafascicular nucleus, upper limb domain
**PGRNl**, Paragigantocellular reticular nucleus, lateral part
**PL**, Prelimbic area cortex
**PP**, Peripenduncular nucleus
**PPN**, Pedunculopontine nucleus
**POST**, Postsubiculum
**PRE**, Presubiculum
**PRNc**, Pontine reticular nucleus, caudal part
**PSV,** Principal sensory nucleus of the trigeminal
**PTLp**, Posterior parietal cortex
**RE**, Reuniens nucleus thalamus
**RSPagl**, Retrosplenial area cortex, agranular part
**RSPd**, Retrosplenial area cortex, dorsal part
**RSPv**, Retrosplenial area cortex, ventral part
**SC**, Superior colliculus
  **SC.m**, Superior colliculus, medial zone
  **SC.cm**, Superior colliculus, centromedial zone
  **SC.cl**, Superior colliculus, centrolateral zone
  **SC.l**, Superior colliculus, lateral zone
**SI**, Substantia innominate

**SNc**, Substantia nigra pars compacta
**SNr**, Substantia nigra pars reticulata
**SOCm**, Superior olivary complex, medial part
**SPFPp**, Subparafascicular nucleus, parvicellular part
**SPVOl**, Spinal motor nucleus of trigeminal, oral lateral part
**SPVOvl**, Spinal motor nucleus of trigeminal, oral ventrolateral part
**SSp,** Somatosensory cortex primary
    **SSp-bfd,** Somatosensory cortex primary, barrel field
    **SSp-ll,** Somatosensory cortex primary, lower limb
    **SSp-m,** Somatosensory cortex primary, mouth
    **SSp-n,** Somatosensory cortex primary, nose
    **SSp-tr,** Somatosensory cortex primary, trunk
    **SSp-ul,** Somatosensory cortex primary, upperlimb
**SSs,** Somatosensory cortex, supplementary
**STN,** Subthalamic nucleus
**SUB**, Subiculum
**SUBd**, Subiculum dorsal
    **SUBdd**, Subiculum dorsal, dorsal part
    **SUBdv**, Subiculum dorsal, ventral part
    **ProSUB**, Prosubiculum
**SUBv**, Subiculum ventral
    **SUBvv**, Subiculum ventral, ventral tip
**TEa**, Temporal association area
**TRN**, Tegmental reticular nucleus
**V,** Trigeminal cranial nucleus
**VII**, Facial motor cranial nucleus
**VISC**, Visceral cortex
**VISal**, Visual cortex, anterolateral part
**VISam**, Visual cortex, anteromedial part
**VISl**, Visual cortex, lateral part
**VISp**, Visual cortex, primary
**VISpl**, Visual cortex, posterolateral part
**VISpm**, Visual cortex, posteromedial part
**VM**, Ventromedial nucleus thalamus
**vmPFC**, Ventromedial prefrontal cortex
**VMH**, Ventromedial hypothalamic nucleus
    **VMH.c**, Ventromedial hypothalamic nucleus, central part
    **VMH.dm**, Ventromedial hypothalamic nucleus, dorsomedial part
    **VMH.vl**, Ventromedial hypothalamic nucleus, ventrolateral part
**ZI**, Zona incerta
    **ZI.m**, Zona incerta, medial part
    **ZI.c**, Zona incerta, central part
    **ZI.l**, Zona incerta, lateral part

## Supplementary Table 2 | Injection Site List.

Injection site coordinates and tracer information for all cases used throughout the study. Cases are identified by Case ID, Injection Site (ROI, region of interest), Tracer, Tracer Type, Figure reference, ARA Level of injection site, and Coordinates for the injection site based on the Allen Reference Atlas Coordinate Frame. Cortex cases can be cross-referenced to flat map injection site location in Supplementary Figure 1 (n=44 mice with 86 total cortical pathways).

| Cortex cases with anterograde projections to Superior Colliculus | | | | | | | | | | |
|---|---|---|---|---|---|---|---|---|---|---|
| **Mouse #** | ***Pathway #*** | **Case ID** | **Injection Site (ROI)** | **Tracer** | **Tracer Type** | **Figure** | **ARA Level** | **ML (X)** | **AP (Y)** | **DV (Z)** |
| **1** | ***1*** | SW110321-04B/A | ACAv interm | PHAL-488 | classic / anterograde | 3g, 4b | 56 | 0.4 | -0.18 | -0.75 |
| **2** | ***2*** | SW110322-03A | MOs caudal | PHAL-488 | classic / anterograde | 2g, 4b | 58 | 0.6 | -0.38 | -0.75 |
| | ***3*** | SW110322-03A | SSp-ul | BDA | classic / anterograde | 4b | 58 | 2.3 | -0.38 | -1.5 |
| **3** | ***4*** | SW110516-02B/A | SSp-n | PHAL-488 | classic / anterograde | 2g, 4b | 50 | 3.1 | 0.45 | -2.7 |
| **4** | ***5*** | SW110613-03B/A | RSPd rostral | PHAL-488 | classic / anterograde | 3b, SF4a, 4b | 83 | 0.6 | -2.98 | -0.25 |
| **5** | ***6*** | SW110614-03B/A | RSPd caudal | PHAL-488 | classic / anterograde | 4b | 88 | 0.6 | -3.45 | -0.25 |
| **6** | ***7*** | SW110615-01B/A | RSPagl | PHAL-488 | classic / anterograde | 3b, 3g, 4b | 91 | 0.9 | -3.78 | -0.25 |
| **7** | ***8*** | SW110615-03B/A | RSPv rostral | PHAL-488 | classic / anterograde | 3b, SF4a, 4b | 73 | 0.5 | -1.85 | -0.75 |
| **8** | ***9*** | SW110808-02A | ORBl | PHAL-488 | classic / anterograde | SF4b, 4b | 37 | 1.75 | 1.95 | -3.25 |
| **9** | ***10*** | SW110808-04A | ORBm | PHAL-488 | classic / anterograde | 3b, 4b | 28 | 0.6 | 2.62 | -2.4 |
| **10** | ***11*** | SW120125-01A | TEa rostral | PHAL-488 | classic / anterograde | 3b, 4b | 78 | 4.25 | -2.35 | -3.4 |
| **11** | ***12*** | SW120403-02A | ACAv rostral | PHAL-488 | classic / anterograde | 3b, SF4a, 4b | 44 | 0.5 | 1.05 | -1.8 |
| **12** | ***13*** | SW120819-04A | VISl | BDA | classic / anterograde | 4b | 92 | 3.5 | -3.88 | -1.75 |
| | ***14*** | SW120819-04A | TEa caudal | PHAL-488 | classic / anterograde | 3b, 4b | 94 | 4.1 | -4.08 | -2.5 |
| **13** | ***15*** | SW121031-03B/A | SSp-tr/ll | AAV-tdTomato | virus / anterograde | 4b | 70 | 1.6 | -1.55 | -0.75 |
| | ***16*** | SW121031-03B/A | ACAd interm | PHAL-647 | classic / anterograde | 3b, SF4a, 4b | 46 | 0.4 | 0.85 | 1.25 |
| | ***17*** | SW121031-03B/A | VISam | AAV-GFP | virus / anterograde | 4b | 83 | 1.6 | -2.98 | -0.5 |
| **14** | ***18*** | SW121031-04B/A | ACAv interm | PHAL-647 | classic / anterograde | 3b, 4b | 54 | 0.4 | 0.2 | -1.6 |
| | ***19*** | SW121031-04B/A | SSp-tr | AAV-tdTomato | virus / anterograde | 2f, 4b | 74 | 2 | -1.98 | -1 |
| | ***20*** | SW121031-04B/A | AUDp interm | AAV-GFP | virus / anterograde | 2g, 2f, 3g, 4b | 84 | 4.1 | -3.1 | -2.5 |
| **15** | ***21*** | SW121210-02A | MOs medial | AAV-GFP | virus / anterograde | 2g, 4b | 49 | 0.5 | 0.55 | -0.85 |
| **16** | ***22*** | SW121221-02A | VISp caudolat | BDA | classic / anterograde | 4b | 96 | 2.75 | -4.28 | -1.25 |
| | ***23*** | SW121221-02A | VISp caudomed | PHAL-488 | classic / anterograde | 2g, 4b | 96 | 2.5 | -4.28 | -1 |
| **17** | ***24*** | SW121221-03A | VISp caudal | BDA | classic / anterograde | 2g, 4b | 95 | 1.8 | -4.12 | -0.75 |
| | ***25*** | SW121221-03A | VISp rostral | PHAL-488 | classic / anterograde | 4b | 86 | 5 | -3.28 | -1.75 |
| **18** | ***26*** | SW151210-01A | ACAd rostral | AAV-tdTomato | virus / anterograde | 3b, 3g, 4b | 36 | 0.3 | 1.85 | -1.25 |
| | ***27*** | SW151210-01A | SSs | AAV-GFP | virus / anterograde | 4b | 53 | 3.25 | 0.15 | -3.25 |
| **19** | ***28*** | SW151210-02A | VISam | AAV-tdTomato | virus / anterograde | 3e, 3g, 4b | 84 | 1.5 | -3.08 | -0.75 |
| | ***29*** | SW151210-02A | SSp-tr | AAV-GFP | virus / anterograde | 3e, 4b | 74 | 2 | -1.95 | -0.7 |
| **20** | ***30*** | SW151211-01A/B | VISp | AAV-GFP | virus / anterograde | 3d, 6a, 4b | 92 | 3 | -3.88 | -1.2 |
| | ***31*** | SW151211-01A/B | ACAd rostral | AAV-tdTomato | virus / anterograde | 3b, 3d, 6a, 4b | 37 | 0.6 | -1.75 | -1.25 |
| | ***32*** | SW151211-01A/B | RSPv caudal | PHAL-647 | classic / anterograde | 3b, 3d, 3g, 6a, 4b | 90 | 0.5 | -3.68 | -0.75 |
| **21** | ***33*** | SW151211-02A | ACAd caudal | AAV-tdTomato | virus / anterograde | 3e, 4b | 58 | 0.4 | -0.38 | -1 |
| | ***34*** | SW151211-02A | VISam | PHAL-647 | classic / anterograde | 3e, 4b | 86 | 1.5 | -3.28 | -0.5 |
| | ***35*** | SW151211-02A | SSp-tr | AAV-GFP | virus / anterograde | 3g, 4b | 76 | 3 | -2.15 | -1.2 |
| **22** | ***36*** | SW151215-03A | PTLp lat | AAV-tdTomato | virus / anterograde | 3b, 4b | 82 | 3.6 | -2.88 | -1.75 |
| **23** | ***37*** | SW170410-03A | SSp-ll | AAV-GFP | virus / anterograde | 3g, 4b | 56 | 2.2 | -0.18 | -1.25 |
| | ***38*** | SW170410-03A | SSp-ul | AAV-tdTomato | virus / anterograde | 3g, 4b | 47 | 2.5 | -0.75 | 1.5 |
| **24** | ***39*** | SW170410-04A | SSp-m | AAV-tdTomato | virus / anterograde | 3g,4b, SF3c | 43 | 2.75 | 1.15 | -2.5 |
| | ***40*** | SW170410-04A | SSp-ul | AAV-GFP | virus / anterograde | 4b, SF3c | 49 | 2.5 | 0.55 | -1.5 |
| **25** | ***41*** | SW171101-01A | MOp-oro | PHAL-647 | classic / anterograde | 3g, 4b | 34 | 2.1 | 2.1 | -2.75 |
| | ***42*** | SW171101-01A | MOp-ul | AAV-tdTomato | virus / anterograde | 4b | 44 | 1.5 | 1.1 | -1.5 |
| | ***43*** | SW171101-01A | MOp-bfd | AAV-GFP | virus / anterograde | 3g, 4b | 38 | 1.8 | 1.65 | -1.4 |
| **26** | ***44*** | SW171130-02A | MOs lateral | AAV-tdTomato | virus / anterograde | 2d, 4b | 44 | 1.1 | 1.1 | -1.5 |
| | ***45*** | SW171130-02A | MOs lateral | AAV-GFP | virus / anterograde | 2d, 4b | 46 | 1.1 | 0.85 | -1.25 |
| | ***46*** | SW171130-02A | MOp-oro | PHAL-647 | classic / anterograde | 2d, 4b | 40 | 1.4 | 1.42 | -1.6 |
| **27** | ***47*** | SW180117-01A | ORBl | PHAL-647 | classic / anterograde | 3b, 4b | 35 | 1.5 | 2.1 | -3.3 |
| | ***48*** | SW180117-01A | MOp-oro | AAV-tdTomato | virus / anterograde | 3g, 4b | 35 | 1.5 | 2.1 | -1.75 |
| | ***49*** | SW180117-01A | MOp-ul | AAV-GFP | virus / anterograde | 3g, 4b | 41 | 1.4 | 1.35 | -1.4 |
| **28** | ***50*** | SW180227-01A | ACAd interm | PHAL-647 | classic / anterograde | 3g, 4b | 42 | 0.5 | 1.25 | -1.4 |
| | ***51*** | SW180227-01A | MOs medial | AAV-tdTomato | virus / anterograde | 3g, 4b | 40 | 0.6 | 1.42 | -1.25 |
| **29** | ***52*** | SW180227-02A | ACAd interm | PHAL-647 | classic / anterograde | 4b | 47 | 0.3 | 0.75 | -1.2 |
| | ***53*** | SW180227-02A | MOs medial | AAV-tdTomato | virus / anterograde | 4b | 47 | 0.4 | 0.75 | -0.75 |
| **30** | ***54*** | SW180228-03A | ACAv caudal | AAV-tdTomato | virus / anterograde | 3b, 4b | 58 | 0.4 | -0.38 | -1.25 |
| | ***55*** | SW180302-04A | VISal | AAV-tdTomato | virus / anterograde | 4b | 91 | 3.4 | -3.88 | -1.6 |
| | ***56*** | SW180302-04A | PTLp lat | AAV-GFP | virus / anterograde | 4b | 82 | 3.5 | -2.88 | -1.65 |
| **31** | ***57*** | SW180302-05A | VISp caudolat | PHAL-647 | classic / anterograde | 2c, 4b | 94 | 2.25 | -3.88 | -1 |
| | ***58*** | SW180302-05A | VISal | AAV-tdTomato | virus / anterograde | 2c, 4b | 90 | 3.4 | -3.68 | -1.3 |
| | ***59*** | SW180302-05A | PTLp lat | AAV-GFP | virus / anterograde | 2c, 4b | 81 | 3.6 | -2.88 | -1.75 |
| **32** | ***60*** | SW180424-01A | PTLp med | AAV-tdTomato | virus / anterograde | 3b, 4b | 77 | 2.25 | -2.55 | -6 |
| | ***61*** | SW180424-01A | PTLp med | PHAL-488 | classic / anterograde | 3g, 4b | 77 | 1.5 | -2.55 | -6 |
| **33** | ***62*** | SW180516-02A | MOp-oro | PHAL-647 | classic / anterograde | 4b | 32 | 2 | 2.25 | -2.5 |
| | ***63*** | SW180516-02A | MOp-bfd | AAV-tdTomato | virus / anterograde | 4b | 40 | 1.6 | 1.42 | -1.6 |
| | ***64*** | SW180516-02A | MOp-tr | AAV-GFP | virus / anterograde | 4b | 52 | 1.6 | 0.25 | -1.3 |
| **34** | ***65*** | SW180522-04A | VISl/pl | AAV-tdTomato | virus / anterograde | 4b | 97 | 3.5 | -4.15 | -2 |
| **35** | ***66*** | SW180713-03A | VISp | AAV-tdTomato | virus / anterograde | 4b | 90 | 2 | -3.98 | -0.8 |
| | ***67*** | SW180713-03A | SSp-tr | PHAL-647 | classic / anterograde | 4b | 68 | 2 | -1.35 | -1.25 |
| | ***68*** | SW180713-03A | AUDd | AAV-GFP | virus / anterograde | 3g | 74 | 4 | -1.95 | -2.1 |
| **36** | ***69*** | SW180713-04A | VISp rostromed | AAV-GFP | virus / anterograde | 3g, 6a, 4b | 87 | 2.25 | -3.38 | -0.75 |
| | ***70*** | SW180713-04A | SSp-tr | AAV-tdTomato | virus / anterograde | 4b, 6a, SF3c | 68 | 2.5 | -1.35 | -1.25 |
| | ***71*** | SW180713-04A | AUDd | PHAL-647 | classic / anterograde | 4b, 6a | 74 | 4 | -1.95 | -2.1 |
| **37** | ***72*** | SW180717-03A | PTLp med | PHAL-488 | classic / anterograde | 3f, 4b | 76 | 1.25 | -2.15 | -0.6 |
| | ***73*** | SW180717-03A | PTLp med | AAV-tdTomato | virus / anterograde | 3f, 4b | 76 | 2.1 | -2.15 | -0.75 |
| **38** | ***74*** | SW180815-01A | VISl | AAV-tdTomato | virus / anterograde | 3g, 4b | 92 | 3.75 | -3.88 | -2 |
| **39** | ***75*** | SW181109-09A | SSp-bfd | AAV-GFP | virus / anterograde | 4b, SF3c | 64 | 3 | -0.95 | -1.5 |
| | ***76*** | SW181109-09A | SSp-bfd | AAV-tdTomato | virus / anterograde | 4b, SF3c | 64 | 3.5 | -0.95 | -2 |
| **40** | ***77*** | SW190315-05A | PL | AAV-tdTomato | virus / anterograde | 3b, 4b, 6a, SF4b | 33 | 0.4 | 2.15 | -2.3 |
| | ***78*** | SW190315-05A | MOs rostral | PHAL-647 | classic / anterograde | 2e, 4b, 6a | 31 | 1.3 | 2.35 | -1.5 |
| | ***79*** | SW190315-05A | MOp-oro | AAV-GFP | virus / anterograde | 2e, 4b, 6a | 31 | 2 | 2.35 | -3 |
| **41** | ***80*** | SW190315-07A | ILA | PHAL-647 | classic / anterograde | 3b, 4b | 37 | 0.5 | 1.75 | -3 |
| **42** | ***81*** | SW190315-09A | AUDp rostral | AAV-tdTomato | virus / anterograde | 4b | 76 | 4.1 | -2.15 | -2.6 |
| | ***82*** | SW190315-09A | AUDp caudal | AAV-GFP | virus / anterograde | 4b | 86 | 4.1 | -3.28 | -2.3 |
| | ***83*** | SW190315-09A | AUDv | PHAL-647 | classic / anterograde | 4b | 70 | 4.2 | -1.56 | -3.1 |
| **43** | ***84*** | SW190327-02A | ORBvl | AAV-GFP | virus / anterograde | 3b, 3g, 4b, SF4b | 33 | 1.25 | 2.15 | -3.1 |
| **44** | ***85*** | SW190327-04A | RSPv interm | AAV-GFP | virus / anterograde | 3b, 4b | 88 | 0.4 | -3.45 | -0.6 |
| | ***86*** | SW190327-04A | VISpm | AAV-tdTomato | virus / anterograde | 3g, 4b | 94 | 1.25 | -4.08 | -0.75 |

# (Supplementary Table 2 : Continued)

**Additional cases used throughout study**

| Mouse # | *Pathway #* | Case ID | Injection Site (ROI) | Tracer | Tracer Type | Figure | ARA Level | ML (X) | AP (Y) | DV (Z) |
|---|---|---|---|---|---|---|---|---|---|---|
| **45** | *87* | SW150916-01A | SC.m | PHAL | classic / anterograde | 4b | 86 | 0.5 | -3.28 | -0.4 |
| | *88* | SW150916-01A | SC.l | BDA | classic / anterograde | 4b | 86 | 1.1 | -3.28 | -2.5 |
| | *89* | SW150916-01A | SC.m | CTB-555 | classic / retrograde | 4b | 86 | 0.5 | -3.28 | -0.4 |
| | *90* | SW150916-01A | SC.l | FG | classic / retrograde | 4b | 86 | 1.1 | -3.28 | -2.5 |
| **46** | *91* | SW150921-01A | SC.m | FG | classic / retrograde | 4b | 90 | 0.3 | -3.68 | -1.6 |
| **47** | *92* | SW160716-01A | SC.cl/l | PHAL | classic / anterograde | 4b | 100 | 0.5 | -4.65 | -1.3 |
| **48** | *93* | SW160716-02A | SC.cm | PHAL | classic / anterograde | 4b | 100 | 0.3 | -4.65 | -1.2 |
| | *94* | SW160716-02A | SC.cm | FG | classic / retrograde | 4b | 86 | 0.5 | -3.28 | -2.3 |
| **49** | *95* | SW170201-05A | SC.m-iw/dg | CTB-647 | classic / retrograde | 4b, 5a | 96 | 0.2 | -4.28 | -1.75 |
| | *96* | SW170201-05A | SC.cl-dg | CTB-555 | classic / retrograde | 4b, 5a | 96 | 0.6 | -4.28 | -2.25 |
| | *97* | SW170201-05A | SC.l-dg | CTB-488 | classic / retrograde | 4b, 5a | 96 | 0.9 | -4.28 | -2.6 |
| **50** | *98* | SW170201-08A | SC.cm-ig/iw/dg | CTB-555 | classic / retrograde | 4b, 5a | 96 | 0.6 | -4.28 | -1.5 |
| | *99* | SW170201-08A | SC.l-ig | FG | classic / retrograde | 4b, 5a | 96 | 1.4 | -4.28 | -2.4 |
| **51** | *100* | SW170215-01A | SC.cm | PHAL | classic / anterograde | 4b | 86 | 0.5 | -3.28 | -2.3 |
| **52** | *101* | SW170315-01A | SC.cl/l | AAV-tdTomato | virus / anterograde | 4b | 90 | 1.5 | -3.68 | -2 |
| **53** | *102* | SW170315-02A | SC.cm | CTB-647 | classic / retrograde | 4b | 90 | 0.5 | -3.68 | -1.5 |
| | *103* | SW170315-02A | SC.cl | FG | classic / retrograde | 4b | 90 | 1.5 | -3.68 | -2.75 |
| **54** | *104* | SW170426-04A | SC.l | AAV-tdTomato | virus / anterograde | 4b | 96 | 1.1 | -4.28 | -2.1 |
| **55** | *105* | SW170830-01A | SC.m | CTB-647 | classic / retrograde | 4b | 100 | 0.3 | -4.65 | -1.5 |
| **56** | *106* | SW170830-02A | SC.cm | CTB-647 | classic / retrograde | 4b | 100 | 0.25 | -4.65 | -1.3 |
| | *107* | SW170830-02A | SC.cl | CTB-488 | classic / retrograde | 4b | 100 | 0.75 | -4.65 | -1.3 |
| **57** | *108* | SW171010-01A | SC.l-ig/iw | AAV-tdTomato | virus / anterograde | 4a-b, SF6c | 90 | 1 | -3.68 | -2.8 |
| | *109* | SW171010-01A | SC.l-dg | PHAL | virus / anterograde | 4a-b | 88 | 1.25 | -3.45 | -2.75 |
| | *110* | SW171010-01A | SC.cl-dg | AAV-GFP | virus / anterograde | 4a-b | 94 | 1 | -4.08 | 2.5 |
| **58** | *111* | SW171010-02A | SC.cm | AAV-tdTomato | virus / anterograde | 4a-b, SF6b | 90 | 0.55 | -3.68 | -2.4 |
| | *112* | SW171010-02A | SC.cl | AAV-GFP | virus / anterograde | 4a-b, SF6b | 90 | 0.75 | -3.68 | -2.5 |
| **59** | *113* | SW171010-03A | SC.m | AAV-tdTomato | virus / anterograde | 4b | 96 | 0.4 | -4.28 | -1.6 |
| **60** | *114* | SW180614-07A | SC.m | AAV-tdTomato | virus / anterograde | 4b | 96 | 0.25 | -4.28 | -1.2 |
| | *115* | SW180614-07A | SC.cl | AAV-GFP | virus / anterograde | 4b | 96 | 0.9 | -4.28 | -1.6 |
| **61** | *116* | SW190619-02A | SC.m/cm | PHAL | classic / anterograde | 4a-b | 90 | 0.5 | -3.68 | -1.5 |
| | *117* | SW190619-02A | SC.cl | AAV-tdTomato | virus / anterograde | 4a-b | 90 | 0.8 | -3.68 | -2.1 |
| **62** | *118* | SW190619-04A | SC.m-iw/dg | PHAL | classic / anterograde | 4a-b, SF6a | 90 | 0.25 | -3.68 | -1.8 |
| **63** | *119* | SW140603-01A | LD | FG | classic / retrograde | SF3a | 69 | 1.2 | -1.45 | -2.6 |
| **64** | *120* | SW140603-03A | MD | FG | classic / retrograde | 5c | 66 | 0.3 | -1.15 | -3.2 |
| **65** | *121* | SW140916-04A | RE | FG | classic / retrograde | 5c | 61 | 0.2 | -0.65 | -4.7 |
| **66** | *122* | SW141021-02A | LP | FG | classic / retrograde | SF3a | 77 | 1.6 | -2.25 | -2.5 |
| **67** | *123* | SW150716-02A | SNr.cl | PHAL-488 | classic / anterograde | 5d | 84 | 1.2 | -3.08 | -5.2 |
| **68** | *124* | SW171002-03A | SNr.l | PHAL-647 | classic / anterograde | 5d | 84 | 1 | -3.08 | -5.25 |
| | *125* | SW171002-03A | SNr.dl | AAV-RFP | classic / anterograde | 5d | 84 | 1.5 | -3.08 | -5.1 |
| | *126* | SW171002-03A | SNr.dm | AAV-GFP | classic / anterograde | 5d | 84 | 0.75 | -3.08 | -5.3 |
| **69** | *127* | SW171213-02A | PF.m | FG | classic / retrograde | 5c | 75 | 0.7 | -2.05 | -3.75 |
| **70** | *128* | SW180508-04A | SNr.v | PHAL-647 | classic / anterograde | 5e | 84 | 1.5 | -3.08 | -5.5 |
| | *129* | SW180508-04A | SNr.dl | AAV-tdTomato | classic / anterograde | 5e | 84 | 1.5 | -3.08 | -5.1 |
| | *130* | SW180508-04A | SNr.dm | AAV-GFP | classic / anterograde | 5e | 84 | 1 | -3.08 | -5.5 |
| **71** | *131* | SW190110-01B | CPc.dm | AAVretro-EF1a-Cre | virus / retrograde | 6b | 57 | 1.6 | -0.28 | -2.5 |
| | *132* | SW190110-01B | MOs | AAV1.CAG-FLEX-tdTom | virus / anterograde | 6b | 47 | 0.5 | 0.75 | -1 |
| **72** | *133* | SW190207-09A | SNr.m | AAV-RFP | virus / anterograde | 5d | 84 | 0.75 | -3.08 | -5.6 |
| **73** | *134* | SW190213-01A | VM/PCN | FG | classic / retrograde | SF3a | 72 | 0.6 | -1.75 | -4 |
| **74** | *135* | SW190829-03A | ZI.medial | AAV-RFP | virus / anterograde | 5b | 74 | 0.6 | -1.95 | -5 |
| **75** | *136* | SW190926-10 | PF.ul | AAVretro-EF1a-Cre | virus / retrograde | 7, 4b | 75 | 0.5 | -2.05 | -3.4 |
| | *137* | SW190926-10 | SC.l-ig | ıSyn-FLEX-TVA-P2A-GFP- | virus / anterograde | 7, 4b | 90 | 1.5 | -3.68 | -2.5 |
| | *138* | SW190926-10 | SC.l-ig | nvA G-deleted-rabies-mCher | virus / retrograde | 7, 4b | 90 | 1.5 | -3.68 | -2.5 |
| **76** | *139* | SW191011-01A | ZI.lateral | PHAL-647 | classic / anterograde | 5b | 71 | 1.75 | -1.65 | -4.5 |
| **77** | *140* | SW191011-02A | ZI.central | PHAL-647 | classic / anterograde | 5b | 74 | 1.25 | -1.95 | -4.3 |

***Cortex regions which do not send anterograde projections to Superior Colliculus***

| | | | | | | | | | | |
|---|---|---|---|---|---|---|---|---|---|---|
| **78** | 141 | SW121220-05A | AId | BDA | classic / anterograde | n/a | 31 | 2.5 | 2.35 | -3.75 |
| **79** | 142 | SW120403-01A | AId | BDA | classic / anterograde | n/a | 27 | 2.3 | 2.75 | -3.75 |
| **80** | 143 | SW110321-04B | AIp | BDA | classic / anterograde | n/a | 56 | 4.1 | -0.18 | -4.6 |
| **81** | 144 | SW110906-02A | AIp | PHAL-488 | classic / anterograde | n/a | 64 | 4.3 | -0.95 | -4.5 |
| **82** | 145 | SW120118-01A | AId/v | BDA | classic / anterograde | n/a | 44 | 3.4 | 1.05 | -4.5 |
| **83** | 146 | SW110906-02A | GU | PHAL-488 | classic / anterograde | n/a | 38 | 2.9 | 1.65 | -3.5 |
| **84** | 147 | SW120125-03A | GU | PHAL-488 | classic / anterograde | n/a | 37 | 2.75 | 1.75 | -3.3 |
| **85** | 148 | SW110905-01A | VISC | BDA | classic / anterograde | n/a | 54 | 4 | 0.02 | -4.1 |
| **86** | 149 | SW120118-02A | VISC | BDA | classic / anterograde | n/a | 66 | 4.4 | -1.15 | -4 |
| **87** | 150 | SW110906-04A | ECT | BDA | classic / anterograde | n/a | 84 | 4.4 | -3.08 | -3.95 |
| **88** | 151 | SW180815-02A | ENT | AAV-tdTomato | virus / anterograde | n/a | 101 | 4.75 | -4.78 | -2.75 |
| **89** | 152 | SW120403-03A | PERI | BDA | classic / anterograde | n/a | 87 | 4.7 | -3.38 | -3.9 |

**Supplementary Table 3 | Proportion of labeling values: sensory cortex ROIs to SC zones-layers.** Values represent proportion of pixel density for the selected ROI (n=1 per cortical area) distributed across each SC zone across all layers. Far right summations correspond to values used in Figure 2g.

| | SC.m | | | | | | | SC.cm | | | | | | | SC.cl | | | | | | | SC.l | | | | Summations | | | |
|---|---|---|---|---|---|---|---|---|---|---|---|---|---|---|---|---|---|---|---|---|---|---|---|---|---|---|---|---|---|
| | SC.m-zo | SC.m-sg | SC.m-op | SC.m-ig | SC.m-iw | SC.m-dg | SC.m-dw | SC.cm-zo | SC.cm-sg | SC.cm-op | SC.cm-ig | SC.cm-iw | SC.cm-dg | SC.cm-dw | SC.cl-zo | SC.cl-sg | SC.cl-op | SC.cl-ig | SC.cl-iw | SC.cl-dg | SC.cl-dw | SC.l-ig | SC.l-iw | SC.l-dg | SC.l-dw | SC.m | SC.cm | SC.cl | SC.l |
| VISp_caudal | 0.45 | 0.57 | 0.08 | 0.00 | 0.00 | 0.00 | 0.00 | 0.00 | 0.05 | 0.01 | 0.00 | 0.00 | 0.00 | 0.00 | 0.00 | 0.00 | 0.00 | 0.00 | 0.00 | 0.00 | 0.00 | 0.00 | 0.00 | 0.00 | 0.00 | 1.10 | 0.06 | 0.00 | 0.00 |
| VISam | 0.00 | 0.04 | 0.13 | 0.22 | 0.09 | 0.03 | 0.00 | 0.00 | 0.07 | 0.15 | 0.13 | 0.07 | 0.03 | 0.02 | 0.00 | 0.02 | 0.07 | 0.02 | 0.02 | 0.01 | 0.02 | 0.00 | 0.00 | 0.00 | 0.02 | 0.51 | 0.47 | 0.16 | 0.02 |
| VISal | 0.00 | 0.10 | 0.12 | 0.04 | 0.00 | 0.00 | 0.00 | 0.01 | 0.34 | 0.50 | 0.05 | 0.00 | 0.00 | 0.00 | 0.00 | 0.01 | 0.00 | 0.00 | 0.00 | 0.00 | 0.00 | 0.00 | 0.00 | 0.00 | 0.00 | 0.26 | 0.90 | 0.01 | 0.00 |
| VISp_caudomed | 0.00 | 0.02 | 0.03 | 0.01 | 0.00 | 0.00 | 0.00 | 0.22 | 0.53 | 0.30 | 0.04 | 0.00 | 0.00 | 0.00 | 0.00 | 0.00 | 0.01 | 0.00 | 0.00 | 0.00 | 0.00 | 0.00 | 0.00 | 0.00 | 0.00 | 0.06 | 1.09 | 0.01 | 0.00 |
| VISp_rostromed | 0.00 | 0.00 | 0.00 | 0.00 | 0.00 | 0.00 | 0.00 | 0.05 | 0.13 | 0.13 | 0.03 | 0.00 | 0.00 | 0.00 | 0.19 | 0.44 | 0.20 | 0.02 | 0.00 | 0.00 | 0.00 | 0.00 | 0.00 | 0.00 | 0.00 | 0.00 | 0.34 | 0.85 | 0.00 |
| AUDp_interm | 0.00 | 0.00 | 0.00 | 0.00 | 0.13 | 0.07 | 0.00 | 0.00 | 0.00 | 0.00 | 0.07 | 0.20 | 0.35 | 0.05 | 0.00 | 0.00 | 0.00 | 0.00 | 0.06 | 0.07 | 0.02 | 0.13 | 0.00 | 0.02 | 0.00 | 0.20 | 0.67 | 0.15 | 0.15 |
| MOs_medial | 0.00 | 0.00 | 0.02 | 0.06 | 0.06 | 0.01 | 0.01 | 0.00 | 0.00 | 0.03 | 0.08 | 0.05 | 0.01 | 0.01 | 0.00 | 0.01 | 0.03 | 0.13 | 0.15 | 0.07 | 0.06 | 0.03 | 0.06 | 0.08 | 0.21 | 0.16 | 0.18 | 0.45 | 0.38 |
| SSp_tr | 0.00 | 0.00 | 0.01 | 0.06 | 0.04 | 0.00 | 0.00 | 0.00 | 0.00 | 0.00 | 0.15 | 0.05 | 0.00 | 0.00 | 0.00 | 0.00 | 0.02 | 0.25 | 0.21 | 0.07 | 0.01 | 0.12 | 0.08 | 0.08 | 0.00 | 0.11 | 0.20 | 0.56 | 0.28 |
| AUDd | 0.00 | 0.00 | 0.02 | 0.04 | 0.00 | 0.00 | 0.00 | 0.00 | 0.00 | 0.00 | 0.18 | 0.03 | 0.00 | 0.00 | 0.00 | 0.00 | 0.00 | 0.19 | 0.13 | 0.06 | 0.00 | 0.09 | 0.24 | 0.18 | 0.00 | 0.06 | 0.21 | 0.38 | 0.51 |
| MOs_caudal | 0.00 | 0.00 | 0.00 | 0.02 | 0.01 | 0.01 | 0.02 | 0.00 | 0.00 | 0.00 | 0.01 | 0.03 | 0.01 | 0.02 | 0.00 | 0.00 | 0.05 | 0.12 | 0.14 | 0.08 | 0.06 | 0.04 | 0.07 | 0.07 | 0.42 | 0.06 | 0.07 | 0.45 | 0.60 |
| MOs_lateral | 0.00 | 0.00 | 0.00 | 0.00 | 0.00 | 0.00 | 0.00 | 0.00 | 0.00 | 0.00 | 0.02 | 0.00 | 0.00 | 0.00 | 0.01 | 0.00 | 0.07 | 0.26 | 0.10 | 0.01 | 0.00 | 0.25 | 0.18 | 0.10 | 0.18 | 0.00 | 0.02 | 0.45 | 0.71 |
| SSp_bfd | 0.00 | 0.00 | 0.00 | 0.00 | 0.00 | 0.00 | 0.00 | 0.00 | 0.00 | 0.00 | 0.00 | 0.00 | 0.00 | 0.00 | 0.00 | 0.00 | 0.00 | 0.25 | 0.06 | 0.00 | 0.00 | 0.51 | 0.30 | 0.05 | 0.00 | 0.00 | 0.00 | 0.31 | 0.86 |
| MOp-bfd | 0.00 | 0.00 | 0.00 | 0.00 | 0.00 | 0.00 | 0.00 | 0.00 | 0.00 | 0.00 | 0.00 | 0.00 | 0.00 | 0.00 | 0.00 | 0.00 | 0.11 | 0.15 | 0.00 | 0.00 | 0.00 | 0.44 | 0.43 | 0.02 | 0.01 | 0.00 | 0.00 | 0.26 | 0.90 |
| MOp-tr | 0.00 | 0.00 | 0.00 | 0.00 | 0.00 | 0.00 | 0.00 | 0.00 | 0.00 | 0.00 | 0.00 | 0.00 | 0.00 | 0.00 | 0.01 | 0.00 | 0.24 | 0.11 | 0.02 | 0.00 | 0.00 | 0.66 | 0.04 | 0.09 | 0.00 | 0.00 | 0.00 | 0.38 | 0.79 |
| MOs_rostral | 0.00 | 0.00 | 0.00 | 0.00 | 0.00 | 0.00 | 0.00 | 0.00 | 0.00 | 0.00 | 0.00 | 0.00 | 0.00 | 0.00 | 0.00 | 0.00 | 0.01 | 0.07 | 0.00 | 0.00 | 0.00 | 0.81 | 0.19 | 0.08 | 0.00 | 0.00 | 0.00 | 0.08 | 1.08 |
| SSp_ll | 0.00 | 0.00 | 0.00 | 0.00 | 0.00 | 0.00 | 0.00 | 0.00 | 0.00 | 0.00 | 0.00 | 0.00 | 0.00 | 0.00 | 0.00 | 0.00 | 0.02 | 0.06 | 0.01 | 0.00 | 0.00 | 0.50 | 0.27 | 0.32 | 0.00 | 0.00 | 0.00 | 0.09 | 1.09 |
| MOp-oro | 0.00 | 0.00 | 0.00 | 0.00 | 0.00 | 0.00 | 0.00 | 0.00 | 0.00 | 0.00 | 0.00 | 0.00 | 0.00 | 0.00 | 0.00 | 0.00 | 0.00 | 0.05 | 0.01 | 0.00 | 0.00 | 0.50 | 0.54 | 0.07 | 0.00 | 0.00 | 0.00 | 0.06 | 1.11 |
| SSp_m | 0.00 | 0.00 | 0.00 | 0.00 | 0.00 | 0.00 | 0.00 | 0.00 | 0.00 | 0.00 | 0.00 | 0.00 | 0.00 | 0.00 | 0.00 | 0.00 | 0.00 | 0.00 | 0.00 | 0.00 | 0.00 | 0.82 | 0.32 | 0.02 | 0.00 | 0.00 | 0.00 | 0.00 | 1.16 |
| SSp_n | 0.00 | 0.00 | 0.00 | 0.00 | 0.00 | 0.00 | 0.00 | 0.00 | 0.00 | 0.00 | 0.00 | 0.00 | 0.00 | 0.00 | 0.00 | 0.00 | 0.00 | 0.00 | 0.00 | 0.00 | 0.00 | 0.81 | 0.32 | 0.04 | 0.00 | 0.00 | 0.00 | 0.00 | 1.17 |
| SSp_ul | 0.00 | 0.00 | 0.00 | 0.00 | 0.00 | 0.00 | 0.00 | 0.00 | 0.00 | 0.00 | 0.00 | 0.00 | 0.00 | 0.00 | 0.00 | 0.00 | 0.00 | 0.00 | 0.00 | 0.00 | 0.00 | 0.64 | 0.39 | 0.14 | 0.00 | 0.00 | 0.00 | 0.00 | 1.17 |
| SSs | 0.00 | 0.00 | 0.00 | 0.00 | 0.00 | 0.00 | 0.00 | 0.00 | 0.00 | 0.00 | 0.00 | 0.00 | 0.00 | 0.00 | 0.00 | 0.00 | 0.00 | 0.00 | 0.00 | 0.00 | 0.00 | 0.89 | 0.25 | 0.03 | 0.00 | 0.00 | 0.00 | 0.00 | 1.17 |

**Supplementary Table 4 | Proportion of labeling values: higher order cortex ROIs to SC zones-layers.** Values represent proportion of pixel density for the selected ROI (n=1 per cortical area) distributed across each SC zone across all layers. Far right summations correspond to values used in Figure 3b.

| | SC.m | | | | | | | SC.cm | | | | | | | SC.cl | | | | | | | SC.l | | | | Summations | | | |
|---|---|---|---|---|---|---|---|---|---|---|---|---|---|---|---|---|---|---|---|---|---|---|---|---|---|---|---|---|---|
| | SC.m-zo | SC.m-sg | SC.m-op | SC.m-ig | SC.m-iw | SC.m-dg | SC.m-dw | SC.cm-zo | SC.cm-sg | SC.cm-op | SC.cm-ig | SC.cm-iw | SC.cm-dg | SC.cm-dw | SC.cl-zo | SC.cl-sg | SC.cl-op | SC.cl-ig | SC.cl-iw | SC.cl-dg | SC.cl-dw | SC.l-ig | SC.l-iw | SC.l-dg | SC.l-dw | SC.m | SC.cm | SC.cl | SC.l |
| RSPv_caudal | 0.01 | 0.14 | 0.40 | 0.25 | 0.51 | 0.25 | 0.04 | 0.00 | 0.04 | 0.16 | 0.11 | 0.21 | 0.03 | 0.04 | 0.00 | 0.01 | 0.01 | 0.02 | 0.01 | 0.01 | 0.05 | 0.00 | 0.00 | 0.00 | 0.00 | 1.61 | 0.61 | 0.12 | 0.00 |
| RSPv_interm | 0.05 | 0.28 | 0.57 | 0.49 | 0.19 | 0.04 | 0.00 | 0.05 | 0.10 | 0.22 | 0.18 | 0.03 | 0.02 | 0.03 | 0.00 | 0.01 | 0.01 | 0.02 | 0.00 | 0.00 | 0.00 | 0.00 | 0.00 | 0.00 | 0.03 | 1.64 | 0.62 | 0.05 | 0.03 |
| RSPd_rostral | 0.00 | 0.06 | 0.30 | 0.41 | 0.33 | 0.06 | 0.05 | 0.00 | 0.02 | 0.15 | 0.23 | 0.30 | 0.05 | 0.03 | 0.00 | 0.00 | 0.03 | 0.01 | 0.07 | 0.02 | 0.04 | 0.00 | 0.00 | 0.01 | 0.16 | 1.20 | 0.79 | 0.18 | 0.16 |
| RSPd_caudal | 0.00 | 0.04 | 0.13 | 0.22 | 0.27 | 0.08 | 0.00 | 0.02 | 0.12 | 0.25 | 0.26 | 0.43 | 0.22 | 0.13 | 0.00 | 0.01 | 0.03 | 0.00 | 0.03 | 0.01 | 0.08 | 0.00 | 0.00 | 0.00 | 0.00 | 0.75 | 1.43 | 0.16 | 0.01 |
| RSPagl | 0.00 | 0.02 | 0.09 | 0.34 | 0.37 | 0.09 | 0.05 | 0.00 | 0.05 | 0.18 | 0.28 | 0.23 | 0.26 | 0.19 | 0.00 | 0.00 | 0.01 | 0.01 | 0.01 | 0.04 | 0.08 | 0.00 | 0.00 | 0.00 | 0.00 | 0.98 | 1.20 | 0.16 | 0.00 |
| PTLp_lat | 0.00 | 0.02 | 0.26 | 0.58 | 0.04 | 0.00 | 0.00 | 0.00 | 0.01 | 0.12 | 0.75 | 0.07 | 0.00 | 0.00 | 0.00 | 0.00 | 0.01 | 0.09 | 0.17 | 0.08 | 0.01 | 0.01 | 0.04 | 0.02 | 0.05 | 0.91 | 0.95 | 0.37 | 0.11 |
| TEa_caudal | 0.00 | 0.01 | 0.06 | 0.08 | 0.46 | 0.25 | 0.09 | 0.00 | 0.01 | 0.02 | 0.14 | 0.21 | 0.39 | 0.16 | 0.00 | 0.00 | 0.00 | 0.03 | 0.02 | 0.18 | 0.23 | 0.01 | 0.00 | 0.00 | 0.00 | 0.95 | 0.92 | 0.46 | 0.01 |
| ACAv_caudal | 0.00 | 0.00 | 0.05 | 0.30 | 0.01 | 0.02 | 0.01 | 0.00 | 0.00 | 0.16 | 0.38 | 0.27 | 0.21 | 0.06 | 0.00 | 0.02 | 0.08 | 0.06 | 0.28 | 0.09 | 0.11 | 0.00 | 0.00 | 0.01 | 0.23 | 0.39 | 1.07 | 0.63 | 0.24 |
| ACAv_interm | 0.00 | 0.01 | 0.05 | 0.14 | 0.13 | 0.10 | 0.14 | 0.00 | 0.01 | 0.05 | 0.20 | 0.26 | 0.20 | 0.32 | 0.00 | 0.01 | 0.03 | 0.04 | 0.08 | 0.14 | 0.23 | 0.00 | 0.00 | 0.03 | 0.16 | 0.58 | 1.03 | 0.52 | 0.20 |
| ACAv_rostral | 0.00 | 0.00 | 0.02 | 0.08 | 0.15 | 0.18 | 0.15 | 0.00 | 0.00 | 0.01 | 0.11 | 0.28 | 0.26 | 0.25 | 0.00 | 0.00 | 0.01 | 0.05 | 0.14 | 0.19 | 0.38 | 0.00 | 0.01 | 0.05 | 0.01 | 0.58 | 0.91 | 0.77 | 0.07 |
| RSPv_rostral | 0.00 | 0.00 | 0.02 | 0.05 | 0.01 | 0.03 | 0.00 | 0.00 | 0.00 | 0.05 | 0.30 | 0.11 | 0.05 | 0.03 | 0.00 | 0.00 | 0.04 | 0.16 | 0.19 | 0.19 | 0.20 | 0.02 | 0.01 | 0.04 | 0.83 | 0.11 | 0.54 | 0.79 | 0.90 |
| PTLp_med | 0.00 | 0.00 | 0.02 | 0.03 | 0.36 | 0.24 | 0.17 | 0.00 | 0.00 | 0.01 | 0.03 | 0.26 | 0.13 | 0.18 | 0.00 | 0.02 | 0.07 | 0.07 | 0.23 | 0.20 | 0.28 | 0.01 | 0.01 | 0.04 | 0.00 | 0.82 | 0.60 | 0.86 | 0.06 |
| ACAd_caudal | 0.00 | 0.00 | 0.00 | 0.06 | 0.01 | 0.02 | 0.03 | 0.00 | 0.00 | 0.02 | 0.13 | 0.10 | 0.03 | 0.05 | 0.00 | 0.07 | 0.23 | 0.21 | 0.58 | 0.19 | 0.29 | 0.00 | 0.01 | 0.04 | 0.24 | 0.13 | 0.34 | 1.57 | 0.30 |
| ACAd_interm | 0.00 | 0.00 | 0.05 | 0.11 | 0.05 | 0.07 | 0.08 | 0.00 | 0.00 | 0.05 | 0.15 | 0.19 | 0.11 | 0.32 | 0.02 | 0.01 | 0.10 | 0.11 | 0.17 | 0.17 | 0.15 | 0.02 | 0.06 | 0.10 | 0.25 | 0.36 | 0.83 | 0.72 | 0.42 |
| ACAd_rostral | 0.00 | 0.00 | 0.00 | 0.03 | 0.13 | 0.06 | 0.08 | 0.00 | 0.00 | 0.00 | 0.05 | 0.27 | 0.03 | 0.35 | 0.00 | 0.00 | 0.01 | 0.10 | 0.28 | 0.18 | 0.10 | 0.03 | 0.11 | 0.28 | 0.22 | 0.31 | 0.70 | 0.69 | 0.64 |
| PL | 0.00 | 0.00 | 0.00 | 0.02 | 0.00 | 0.00 | 0.00 | 0.00 | 0.00 | 0.02 | 0.26 | 0.10 | 0.17 | 0.28 | 0.00 | 0.00 | 0.00 | 0.01 | 0.25 | 0.11 | 0.29 | 0.00 | 0.12 | 0.20 | 0.52 | 0.02 | 0.83 | 0.65 | 0.84 |
| ORBm | 0.00 | 0.00 | 0.01 | 0.12 | 0.00 | 0.00 | 0.00 | 0.00 | 0.00 | 0.11 | 0.10 | 0.12 | 0.06 | 0.24 | 0.00 | 0.07 | 0.09 | 0.07 | 0.13 | 0.22 | 0.50 | 0.03 | 0.17 | 0.32 | 0.00 | 0.13 | 0.62 | 1.08 | 0.51 |
| ORBvl | 0.00 | 0.00 | 0.01 | 0.04 | 0.00 | 0.00 | 0.00 | 0.00 | 0.00 | 0.02 | 0.09 | 0.08 | 0.03 | 0.00 | 0.01 | 0.00 | 0.03 | 0.29 | 0.41 | 0.22 | 0.05 | 0.06 | 0.33 | 0.13 | 0.55 | 0.05 | 0.22 | 1.01 | 1.06 |
| ILA | 0.00 | 0.02 | 0.04 | 0.09 | 0.10 | 0.20 | 0.08 | 0.00 | 0.04 | 0.09 | 0.03 | 0.02 | 0.01 | 0.13 | 0.04 | 0.12 | 0.39 | 0.06 | 0.05 | 0.06 | 0.02 | 0.13 | 0.15 | 0.19 | 0.26 | 0.54 | 0.33 | 0.74 | 0.73 |
| TEa_rostral | 0.00 | 0.01 | 0.09 | 0.11 | 0.17 | 0.20 | 0.13 | 0.00 | 0.00 | 0.00 | 0.03 | 0.14 | 0.10 | 0.04 | 0.00 | 0.00 | 0.00 | 0.03 | 0.16 | 0.15 | 0.16 | 0.24 | 0.29 | 0.23 | 0.06 | 0.72 | 0.30 | 0.49 | 0.82 |
| ORBl | 0.00 | 0.00 | 0.00 | 0.00 | 0.00 | 0.00 | 0.00 | 0.00 | 0.00 | 0.00 | 0.00 | 0.00 | 0.00 | 0.00 | 0.00 | 0.00 | 0.01 | 0.16 | 0.11 | 0.01 | 0.02 | 1.00 | 0.61 | 0.11 | 0.14 | 0.00 | 0.00 | 0.30 | 1.86 |

## Supplementary Table 5 | Morphometric *p*-values.

Table of pairwise comparisons using the Wilcoxon rank sum test. Adjustments for *p*-value were made for multiple comparisons using the false discovery rate (fdr) method. *P*-values are listed for the following neuronal morphometric parameters: contraction, width, number of bifurcations, branch path length, fractal dimension, fragmentation, height, number of branches, and height/width ratio.

**Contraction**

| | PF/MPT - SC.m | RE - SC.m | LD - SC.m | PF/MPT - SC.cm | RE - SC.cm | LD - SC.cm | PF/MPT - SC.cl | RE - SC.cl |
|---|---|---|---|---|---|---|---|---|
| RE - SC.m | 0.0019 | - | - | - | - | - | - | - |
| LD - SC.m | 0.024 | 0.5253 | - | - | - | - | - | - |
| PF/MPT - SC.cm | 0.9578 | 0.0064 | 0.049 | - | - | - | - | - |
| RE - SC.cm | 0.1386 | 0.052 | 0.1172 | 0.2261 | - | - | - | - |
| LD - SC.cm | 0.1439 | 0.4747 | 0.2057 | 0.1412 | 0.5471 | - | - | - |
| PF/MPT - SC.cl | 0.2981 | 0.0019 | 0.0503 | 0.5471 | 0.0166 | 0.093 | - | - |
| RE - SC.cl | 0.4555 | 0.1386 | 0.4747 | 0.553 | 0.7076 | 0.7261 | 0.1439 | - |
| PF/MPT - SC.l | 0.4946 | 0.0064 | 0.052 | 0.7226 | 0.052 | 0.093 | 0.82 | 0.2981 |

**Width**

| | PF/MPT - SC.m | RE - SC.m | LD - SC.m | PF/MPT - SC.cm | RE - SC.cm | LD - SC.cm | PF/MPT - SC.cl | RE - SC.cl |
|---|---|---|---|---|---|---|---|---|
| RE - SC.m | 0.0058 | - | - | - | - | - | - | - |
| LD - SC.m | 0.2038 | 0.00068 | - | - | - | - | - | - |
| PF/MPT - SC.cm | 0.0844 | 0.2038 | 0.01633 | - | - | - | - | - |
| RE - SC.cm | 0.00057 | 0.03547 | 0.00057 | 0.01554 | - | - | - | - |
| LD - SC.cm | 0.73327 | 0.00738 | 0.03547 | 0.02743 | 0.00057 | - | - | - |
| PF/MPT - SC.cl | 0.00134 | 0.01633 | 0.00719 | 0.01518 | 0.35848 | 0.00719 | - | - |
| RE - SC.cl | 0.00599 | 0.00068 | 0.03547 | 0.01633 | 0.0058 | 0.03547 | 0.04028 | - |
| PF/MPT - SC.l | 0.00266 | 0.00057 | 0.02381 | 0.00917 | 0.00188 | 0.02381 | 0.00899 | 0.2038 |

**Number of Bifurcations**

| | PF/MPT - SC.m | RE - SC.m | LD - SC.m | PF/MPT - SC.cm | RE - SC.cm | LD - SC.cm | PF/MPT - SC.cl | RE - SC.cl |
|---|---|---|---|---|---|---|---|---|
| RE - SC.m | 0.0003 | - | - | - | - | - | - | - |
| LD - SC.m | 0.02569 | 0.10155 | - | - | - | - | - | - |
| PF/MPT - SC.cm | 0.01038 | 0.06615 | 0.05382 | - | - | - | - | - |
| RE - SC.cm | 0.00037 | 0.01038 | 0.02569 | 0.67932 | - | - | - | - |
| LD - SC.cm | 0.03429 | 0.02823 | 0.06615 | 0.14814 | 0.47941 | - | - | - |
| PF/MPT - SC.cl | 0.00514 | 0.8358 | 0.14988 | 0.27131 | 0.12966 | 0.10489 | - | - |
| RE - SC.cl | 0.03429 | 0.06615 | 0.06615 | 0.67771 | 0.8358 | 0.47941 | 0.17882 | - |
| PF/MPT - SC.l | 0.01546 | 0.9272 | 0.44167 | 0.54313 | 0.17882 | 0.17241 | 0.9185 | 0.48076 |

**Branch Path Length**

| | PF/MPT - SC.m | RE - SC.m | LD - SC.m | PF/MPT - SC.cm | RE - SC.cm | LD - SC.cm | PF/MPT - SC.cl | RE - SC.cl |
|---|---|---|---|---|---|---|---|---|
| RE - SC.m | 0.00000072 | - | - | - | - | - | - | - |
| LD - SC.m | 0.01028 | 0.23028 | - | - | - | - | - | - |
| PF/MPT - SC.cm | 0.11847 | 0.00061 | 0.04699 | - | - | - | - | - |
| RE - SC.cm | 0.00000072 | 0.28913 | 0.33678 | 0.00072 | - | - | - | - |
| LD - SC.cm | 0.01654 | 0.03506 | 0.18701 | 0.18701 | 0.06164 | - | - | - |
| PF/MPT - SC.cl | 0.01654 | 0.00148 | 0.04699 | 0.30984 | 0.00548 | 0.77508 | - | - |
| RE - SC.cl | 0.02877 | 0.51997 | 0.18701 | 0.33678 | 0.82103 | 0.77143 | 0.49678 | - |
| PF/MPT - SC.l | 0.03796 | 0.04699 | 0.18701 | 0.26623 | 0.13664 | 0.90476 | 0.82103 | 0.49678 |

**Fractal Dimension**

| | PF/MPT - SC.m | RE - SC.m | LD - SC.m | PF/MPT - SC.cm | RE - SC.cm | LD - SC.cm | PF/MPT - SC.cl | RE - SC.cl |
|---|---|---|---|---|---|---|---|---|
| RE - SC.m | 0.00531 | - | - | - | - | - | - | - |
| LD - SC.m | 0.06165 | 0.88571 | - | - | - | - | - | - |
| PF/MPT - SC.cm | 0.83907 | 0.01258 | 0.08571 | - | - | - | - | - |
| RE - SC.cm | 0.12321 | 0.0526 | 0.18775 | 0.15982 | - | - | - | - |
| LD - SC.cm | 0.18153 | 0.40418 | 0.42562 | 0.35604 | 0.76544 | - | - | - |
| PF/MPT - SC.cl | 0.08397 | 0.00012 | 0.02877 | 0.18775 | 0.00088 | 0.04476 | - | - |
| RE - SC.cl | 0.11424 | 0.42562 | 0.76544 | 0.18701 | 0.63901 | 0.88571 | 0.04476 | - |
| PF/MPT - SC.l | 0.14259 | 0.01258 | 0.04762 | 0.35532 | 0.01258 | 0.04762 | 0.43636 | 0.04762 |

**Fragmentation**

| | PF/MPT - SC.m | RE - SC.m | LD - SC.m | PF/MPT - SC.cm | RE - SC.cm | LD - SC.cm | PF/MPT - SC.cl | RE - SC.cl |
|---|---|---|---|---|---|---|---|---|
| RE - SC.m | 0.00000026 | - | - | - | - | - | - | - |
| LD - SC.m | 0.00621 | 0.06137 | - | - | - | - | - | - |
| PF/MPT - SC.cm | 0.8645 | 0.000025 | 0.01805 | - | - | - | - | - |
| RE - SC.cm | 0.00000026 | 0.00621 | 0.00629 | 0.000013 | - | - | - | - |
| LD - SC.cm | 0.00621 | 0.51997 | 0.15238 | 0.01805 | 0.1981 | - | - | - |
| PF/MPT - SC.cl | 0.0626 | 0.00000064 | 0.00629 | 0.02752 | 0.00000026 | 0.00629 | - | - |
| RE - SC.cl | 0.00621 | 0.64756 | 0.15238 | 0.01805 | 0.31541 | 0.93782 | 0.00629 | - |
| PF/MPT - SC.l | 0.95305 | 0.0001 | 0.02597 | 0.95305 | 0.00018 | 0.02597 | 0.29763 | 0.02597 |

**Height**

| | PF/MPT - SC.m | RE - SC.m | LD - SC.m | PF/MPT - SC.cm | RE - SC.cm | LD - SC.cm | PF/MPT - SC.cl | RE - SC.cl |
|---|---|---|---|---|---|---|---|---|
| RE - SC.m | 0.00000026 | - | - | - | - | - | - | - |
| LD - SC.m | 0.0048 | 0.00057 | - | - | - | - | - | - |
| PF/MPT - SC.cm | 0.00327 | 0.00057 | 0.02017 | - | - | - | - | - |
| RE - SC.cm | 0.0000001 | 2.7E-12 | 0.00046 | 0.0265 | - | - | - | - |
| LD - SC.cm | 0.0048 | 0.03979 | 0.04472 | 0.05331 | 0.06077 | - | - | - |
| PF/MPT - SC.cl | 0.00019 | 0.00103 | 0.00629 | 0.30614 | 0.12993 | 0.12993 | - | - |
| RE - SC.cl | 0.0048 | 0.00057 | 0.04472 | 0.5042 | 0.0048 | 0.04472 | 0.51788 | - |
| PF/MPT - SC.l | 0.0024 | 0.05331 | 0.04762 | 0.06679 | 0.33766 | 0.55556 | 0.12993 | 0.03008 |

**Number of Branches**

| | PF/MPT - SC.m | RE - SC.m | LD - SC.m | PF/MPT - SC.cm | RE - SC.cm | LD - SC.cm | PF/MPT - SC.cl | RE - SC.cl |
|---|---|---|---|---|---|---|---|---|
| RE - SC.m | 0.00022 | - | - | - | - | - | - | - |
| LD - SC.m | 0.01835 | 0.10961 | - | - | - | - | - | - |
| PF/MPT - SC.cm | 0.00951 | 0.05037 | 0.05037 | - | - | - | - | - |
| RE - SC.cm | 0.00022 | 0.00951 | 0.01835 | 0.66578 | - | - | - | - |
| LD - SC.cm | 0.01835 | 0.01835 | 0.06367 | 0.26852 | 0.64114 | - | - | - |
| PF/MPT - SC.cl | 0.00323 | 0.74031 | 0.12302 | 0.18623 | 0.12793 | 0.06367 | - | - |
| RE - SC.cl | 0.01835 | 0.05037 | 0.06367 | 1 | 0.95915 | 0.4047 | 0.10961 | - |
| PF/MPT - SC.l | 0.0156 | 1 | 0.48076 | 0.23764 | 0.10961 | 0.10961 | 0.95915 | 0.41544 |

**Height/Width Ratio**

| | PF/MPT - SC.m | RE - SC.m | LD - SC.m | PF/MPT - SC.cm | RE - SC.cm | LD - SC.cm | PF/MPT - SC.cl | RE - SC.cl |
|---|---|---|---|---|---|---|---|---|
| RE - SC.m | 0.00000039 | - | - | - | - | - | - | - |
| LD - SC.m | 0.00527 | 0.04482 | - | - | - | - | - | - |
| PF/MPT - SC.cm | 0.00527 | 0.00016 | 0.01797 | - | - | - | - | - |
| RE - SC.cm | 0.00000021 | 0.00527 | 0.0058 | 0.00246 | - | - | - | - |
| LD - SC.cm | 0.00527 | 0.00527 | 0.04114 | 0.12857 | 0.04482 | - | - | - |
| PF/MPT - SC.cl | 0.00016 | 0.01797 | 0.01185 | 0.01518 | 0.84875 | 0.09375 | - | - |
| RE - SC.cl | 0.00527 | 0.09805 | 0.04114 | 0.02981 | 0.93215 | 0.12857 | 0.84875 | - |
| PF/MPT - SC.l | 0.00343 | 0.07907 | 0.79654 | 0.00974 | 0.00016 | 0.02597 | 0.0045 | 0.02597 |
