# Supplementary material for: Organization of the inputs and outputs of the mouse superior colliculus
Source: Nat Commun. 2021 Jun 28;12:4004. doi: 10.1038/s41467-021-24241-2 (PMC8239028; doi:10.1038/s41467-021-24241-2)
Supplement: Supplementary file 2 — Reporting Summary [file 41467_2021_24241_MOESM2_ESM.pdf]

nature research

Corresponding author(s): Hong-Wei Dong

Last updated by author(s): YYYY-MM-DD

Reporting Summary

Nature Research wishes to improve the reproducibility of the work that we publish. This form provides structure for consistency and transparency in reporting. For further information on Nature Research policies, see our Editorial Policies and the Editorial Policy Checklist.

Statistics

For all statistical analyses, confirm that the following items are present in the figure legend, table legend, main text, or Methods section.

n/a	Confirmed	
☐	☒	The exact sample size (*n*) for each experimental group/condition, given as a discrete number and unit of measurement
☐	☒	A statement on whether measurements were taken from distinct samples or whether the same sample was measured repeatedly
☐	☒	The statistical test(s) used AND whether they are one- or two-sided<br>*Only common tests should be described solely by name; describe more complex techniques in the Methods section.*
☒	☐	A description of all covariates tested
☒	☐	A description of any assumptions or corrections, such as tests of normality and adjustment for multiple comparisons
☒	☐	A full description of the statistical parameters including central tendency (e.g. means) or other basic estimates (e.g. regression coefficient) AND variation (e.g. standard deviation) or associated estimates of uncertainty (e.g. confidence intervals)
☐	☒	For null hypothesis testing, the test statistic (e.g. F, t, r) with confidence intervals, effect sizes, degrees of freedom and P value noted<br>*Give P values as exact values whenever suitable.*
☒	☐	For Bayesian analysis, information on the choice of priors and Markov chain Monte Carlo settings
☒	☐	For hierarchical and complex designs, identification of the appropriate level for tests and full reporting of outcomes
☒	☐	Estimates of effect sizes (e.g. Cohen's d, Pearson's r), indicating how they were calculated

Our web collection on statistics for biologists contains articles on many of the points above.

Software and code

Policy information about availability of computer code

Data collection	No software was used to collect data.
Data analysis	All data used in this study are available from the corresponding author upon reasonable request. Anatomical tracer image data is available through our iConnectome viewer as part of the Mouse Connectome Project at USC (http://www.mouseconnectome.org). All reconstructions are being made freely available in the Dong archive of www.NeuroMorpho.Org. We used various software to navigate images and data including, FIJI/ImageJ (v1.53a), neuTube (v1.0z), Adobe Photoshop (v21.2.2), Aivia (v8.8.1, DRVision), and RStudio (v1.1.463).<br><br>All custom code that we developed for data analysis is available at https://github.com/lhgarciaa/sc_project and https://github.com/lhgarciaa/sc_analysis. Code used for other data analyses are freely accessible. For the Louvain algorithm implementation, the Brain Connectivity Toolbox (BCT) was employed, available at https://sites.google.com/site/bctnet/. For matrix visualization, the grid_communities algorithm for matrix visualizations are available at https://github.com/aestrivex/bctpy, and https://sites.google.com/site/bctnet/. Geometric processing of neuron models was performed using the Quantitative Imaging Toolkit (QIT), available here http://cabeen.io/qitwiki. We also computed persistence diagrams using NeuronTools (https://github.com/Nevermore520/NeuronTools; RID:SCR_017450) and then computed inter-neuron distances using the Wasserstein metric (https://bitbucket.org/grey_narn/geom_matching/src).<br>In addition, we have used several open-source Python packages: scipy (v0.17.0), matplotlib (v1.5.1), ipython (v2.4.1), ipython-genutils (v0.2.0), jupyter (v1.0.0), jupyter-client (v5.2.3), jupyter-console (v5.2.0), jupyter-core (v4.4.0), pandas (v0.23.3), sympy (v1.2), nose (v1.3.7), pydot (v1.2.3), bctpy (v0.5.0), pyflakes (v1.1.0), pandas (v0.23.3), cycler (v0.9.0), plotly (v3.0.0).

For manuscripts utilizing custom algorithms or software that are central to the research but not yet described in published literature, software must be made available to editors and reviewers. We strongly encourage code deposition in a community repository (e.g. GitHub). See the Nature Research guidelines for submitting code & software for further information.

Data

Policy information about availability of data

All manuscripts must include a data availability statement. This statement should provide the following information, where applicable:

- Accession codes, unique identifiers, or web links for publicly available datasets
- A list of figures that have associated raw data
- A description of any restrictions on data availability

Anatomical tracer image data is available through our iConnectome viewer as part of the Mouse Connectome Project at USC (http://www.mouseconnectome.org). Supplementary Table 2 includes a complete list of raw data cases associated with each figure. Additional data that support the findings of this study that are not readily accessible online are available from the corresponding author upon reasonable request.

Field-specific reporting

Please select the one below that is the best fit for your research. If you are not sure, read the appropriate sections before making your selection.

[x] Life sciences [ ] Behavioural & social sciences [ ] Ecological, evolutionary & environmental sciences

For a reference copy of the document with all sections, see nature.com/documents/nr-reporting-summary-flat.pdf

Life sciences study design

All studies must disclose on these points even when the disclosure is negative.

Sample size	No sample size calculation was performed. Systematic anatomical tracer injections that were made into identical locations of a given cortical area in two different mice resulted in identical projection patterns SC. This sample size is similar to those we used in our earlier studies (Zingg et al, 2014; Hintiryan et al, 2016). Several other injection cases were repeated as further controls and resulted in identical labeling patterns. In total, we have listed 89 total mice that contain 152 different tracers that we believe sufficiently describe the anatomical network connectivity of the cortico-tectal pathways and other connections of the superior colliculus.
Data exclusions	No analyzed data was excluded.
Replication	Each experiment was replicated at least twice, and further cross-validated by multiple experiments using different tracer combinations. Each replication attempt was successful and produced consistent results. Experiments were also cross-referenced to other similar cases in our iConnectome online database. In many cases, we have both anterograde and retrograde data to describe any given anatomical pathway and the combination of the two tracer types provides cross-validation. Anatomical connection of anterograde tracing results are cross-validated by retrograde labeling injections at the anterograde fiber terminal fields and vice versa. For example, if area A projected to area B, this same axonal projection can be labeled by an anterograde injection in area A or a retrograde injection in area B.
Randomization	No method of randomization was used in any of the experiments. Our study does not involve multiple groups that receive different treatments, and we do not make statistical comparisons.
Blinding	The investigators were not blinded as the injection site location is apparent when analyzing the distribution of anatomical labeling throughout the brain.

Reporting for specific materials, systems and methods

We require information from authors about some types of materials, experimental systems and methods used in many studies. Here, indicate whether each material, system or method listed is relevant to your study. If you are not sure if a list item applies to your research, read the appropriate section before selecting a response.

Materials & experimental systems

n/a	Involved in the study	
[ ]	[x]	Antibodies
[x]	[ ]	Eukaryotic cell lines
[x]	[ ]	Palaeontology and archaeology
[ ]	[x]	Animals and other organisms
[x]	[ ]	Human research participants
[x]	[ ]	Clinical data
[x]	[ ]	Dual use research of concern

Methods

n/a	Involved in the study	
[x]	[ ]	ChIP-seq
[x]	[ ]	Flow cytometry
[x]	[ ]	MRI-based neuroimaging

Antibodies

Antibodies used	A rabbit anti-PHAL primary antibody (Vector Laboratories Cat# AS-2300, RRID:AB_2313686) and a donkey anti-rabbit IgG

	secondary antibody conjugated to either AlexaFluor 488 (Thermo Fisher Scientific Cat# A-31571, RRID:AB_162542), or Cy3 (Jackson ImmunoResearch Labs Cat# 715-165-151, RRID:AB_2315777) were used to localize PHAL tracer within brain tissue sections. A mouse anti-Cre Recombinase Antibody, clone 2D8 (Millipore Sigma Cat# MAB3120) and a donkey anti-mouse IgG conjugated with Alexa Fluor 647 secondary antibody (Life Technologies Cat# A-31571), (Life Technologies Cat #A-31571) was used to localize retrograde Cre labeling within brain tissues.
Validation	Rabbit anti-PHAL antibody (Vector Laboratories Cat# AS-2300, RRID:AB_2313686) has been used and validated in our previous studies (Biag et al, 2012; Hintiryan et al, 2016). Mouse-anti-Cre Recombinase antibody (clone 2D8, Millipore Sigma Cat# MAB3120, RRID:AB_2085748) has been used and validated in previous studies (Schwarz et al, 2015; Hintiryan et al, 2019).

Animals and other organisms

Policy information about studies involving animals; ARRIVE guidelines recommended for reporting animal research

Laboratory animals	All tracer experimental data was generated using 2-6 month-old male C57BL/6 mice (Jackson Laboratories).
Wild animals	This study did not involve wild animals.
Field-collected samples	This study did not involve field-collected samples.
Ethics oversight	All experiments were performed according to the regulatory standards set by the National Institutes of Health Guide for the Care and Use of Laboratory Animals and by the institutional guidelines described by the USC Institutional Animal Care and Use Committee.

Note that full information on the approval of the study protocol must also be provided in the manuscript.
